# Evaluation of Novel Spiro-pyrrolopyridazine Derivatives as Anticancer Compounds: In Vitro Selective Cytotoxicity, Induction of Apoptosis, EGFR Inhibitory Activity, and Molecular Docking Analysis

**DOI:** 10.1021/acsomega.4c00794

**Published:** 2024-05-20

**Authors:** Harika Atmaca, Suleyman Ilhan, Çisil Çamli Pulat, Buse Aysen Dundar, Metin Zora

**Affiliations:** † Department of Biology, Faculty of Engineering and Natural Sciences, Manisa Celal Bayar University, Manisa 45140, Turkey; ‡ Applied Science Research Center, Manisa Celal Bayar University, Manisa 45140, Turkey; § Department of Chemistry, Middle East Technical University, Ankara 06800, Turkey

## Abstract

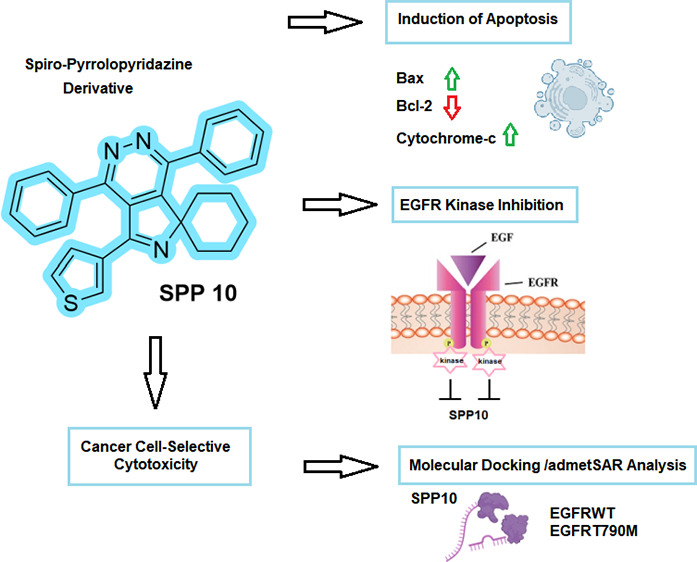

Cancer, characterized by uncontrolled cell proliferation, remains a global health challenge. Despite advancements in cancer treatment, drug resistance and adverse effects on normal cells remain challenging. The epidermal growth factor receptor (EGFR), a transmembrane tyrosine kinase protein, is crucial in controlling cell proliferation and is implicated in various cancers. Here, the cytotoxic and apoptotic potential of 21 newly synthesized spiro-pyrrolopyridazine (SPP) derivatives was investigated on breast (MCF-7), lung (H69AR), and prostate (PC-3) cancer cells. XTT assay was used for cytotoxicity assessment. Flow cytometry and western blot (WB) analyses were conducted for apoptosis detection. Additionally, the EGFR inhibitory potential of these derivatives was evaluated via a homogeneous time-resolved fluorescence (HTRF) assay, and WB and molecular docking studies were conducted to analyze the binding affinities of SPP10 with EGFR. SPPs, especially SPP10, exhibit significant cytotoxicity across MCF-7, H69AR, and PC-3 cancer cells with IC_50_ values of 2.31 ± 0.3, 3.16 ± 0.8, and 4.2 ± 0.2 μM, respectively. Notably, SPP10 demonstrates selective cytotoxicity against cancer cells with a low impact on nontumorigenic cells (IC_50_ value: 26.8 ± 0.4 μM). Flow cytometric analysis demonstrated the potent induction of apoptotic cell death by SPP10 in all of the tested cancer cells. Western blot analysis revealed the involvement of key apoptotic proteins, with SPP10 notably inhibiting antiapoptotic Bcl-2 while inducing pro-apoptotic Bax and cytochrome c. SPP10 exhibited significant EGFR kinase inhibitory activity, surpassing the efficacy of the reference drug erlotinib. Molecular docking studies support these findings, revealing strong binding affinities of SPP10 with both wild-type and mutated EGFR. The study underscores the significance of heterocyclic compounds, particularly spiro-class heterocyclic molecules, in advancing cancer research. Overall, SPP10 emerges as a promising candidate for further investigations in cancer treatment, combining potent cytotoxicity, apoptotic induction, and targeted EGFR inhibition.

## Introduction

Cancer refers to a wide variety of illnesses characterized by uncontrolled cell proliferation. Different types of cancers share traits such as abnormal cell proliferation, tissue invasion, and a propensity to spread through blood vessels and lymphatic systems.^1^ Escape from apoptosis, known as programmed cell death, is another characteristic feature of cancer cells.^2^ As the COVID-19 epidemic causes delays in the diagnosis and treatment, restriction of health systems, including the suspension of screening programs, and epidemic-related disruptions, there is a short-term decrease in the number of cancer cases. This decline is followed by an increase in late-stage cancer diagnoses and, in certain contexts, an increase in cancer-related deaths. According to 2024 cancer statistics data, breast, lung, and prostate cancers are the most diagnosed cancer types in the world.^3^ Although numerous drugs and diagnostic techniques have emerged to treat certain types of cancers, intrinsic and acquired drug resistance and the destructive effects of these drugs on normal cells limit the use of these new drugs. Therefore, there is an urgent need to discover novel anticancer agents that do not harm healthy cells and trigger apoptotic cell death.

The transmembrane tyrosine kinase protein known as the epidermal growth factor receptor (EGFR) is crucial for regulating cellular processes such as proliferation, metastasis, and apoptosis within human epithelial cells. Serving as a receptor for various members of the EGF family, this functionality has been substantiated by an array of studies.^4^ Amplification of the EGFR gene has been associated with several malignancies, including but not limited to head and neck cancers, breast cancer, prostate cancer, esophageal cancer, and lung cancer.^5^ Oncogenic drivers, such as in-frame deletions of exon 19 and the L858R mutation, are identified as mutations within the EGFR kinase adenosine triphosphate (ATP) binding domain. Additionally, the T790M mutation, often referred to as the secondary “guardian” mutation, augments ATP binding affinity and is prevalent in various cancer types. Consequently, EGFR has been acknowledged as a valuable and promising therapeutic target for cancer treatment. Exploration into inhibiting the activity of mutant EGFR ATP-binding domains has led to the development of several FDA-approved EGFR tyrosine kinase inhibitors (TKIs). Remarkably, the administration of TKIs in cancer patients has yielded substantial responses and contributed to extended survival rates, as indicated by research findings.^6^

Heterocyclic molecules hold significant importance in the realm of organic chemistry, given their pivotal roles across a broad spectrum of applications.^7^ More than 85% of biologically active pharmaceutical substances include heterocyclic units.^8^ This observation demonstrates the crucial role heterocyclic molecules play in medication chemistry. Among them, spiro-class heterocyclic molecules exhibit distinctive character, particularly within the realm of biologically active compounds. Notably, it is the combination of the spiro unit within these molecules and the fused systems that impart them with critical properties.^9^ Consequently, spiro and fused systems have consistently been focal points of interest among organic chemists.

**Scheme 1:**
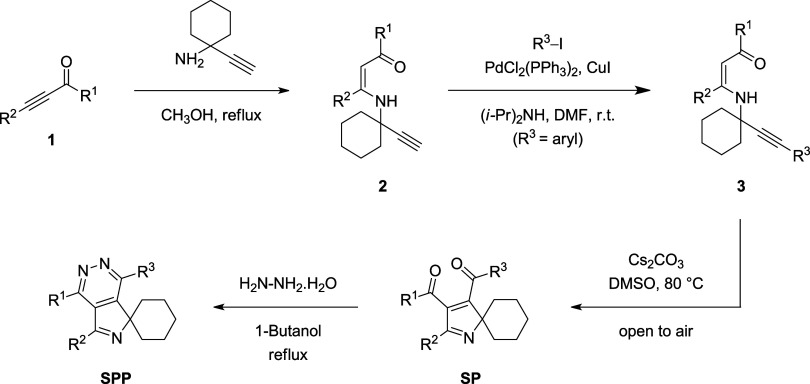
Synthesis of Spiro-pyrrolopyridazines (SPPs)

Recently, we have synthesized 21 new spiro-pyrrolopyridazine derivatives (SPPs) from α,β-alkynic ketones **1** via intermediacy of cyclohexane-embedded *N*-propargylic β-enaminones **2** and **3** and spiro-2*H*-pyrroles (SPs) (for details, see Scheme 1, Table 1, and the Supporting Information).^10^ Notably, β-enaminones are also valuable building blocks and pharmacophores in drug development.^11^ Moreover, in recent years, N-heterocyclic derivatives have been exposed as potential EGFR inhibitors and specifically target the mutated tyrosine kinase domain of EGFR.^12^ In this study, leveraging the distinctive characteristics of spiro-class heterocyclic molecules, we have aimed to assess the apoptotic and antiproliferative activities of these spiro-pyrrolopyridazine derivatives (SPPs). By focusing on EGFR, a pivotal protein in cancer progression, our research aims to contribute insights into the development of targeted cancer therapies, addressing the limitations associated with current treatments including drug resistance and adverse effects on normal cells. By conducting thorough assessments, our goal is to determine the effectiveness of these SPP derivatives as promising contenders for the progression of strategies in cancer treatment.

**Table 1:**
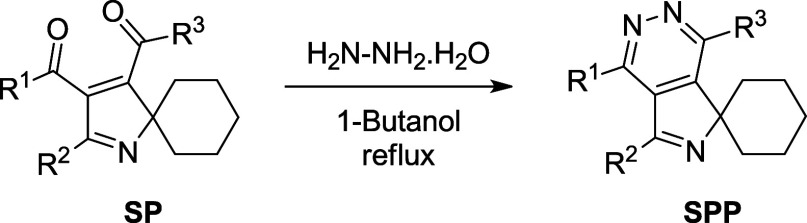

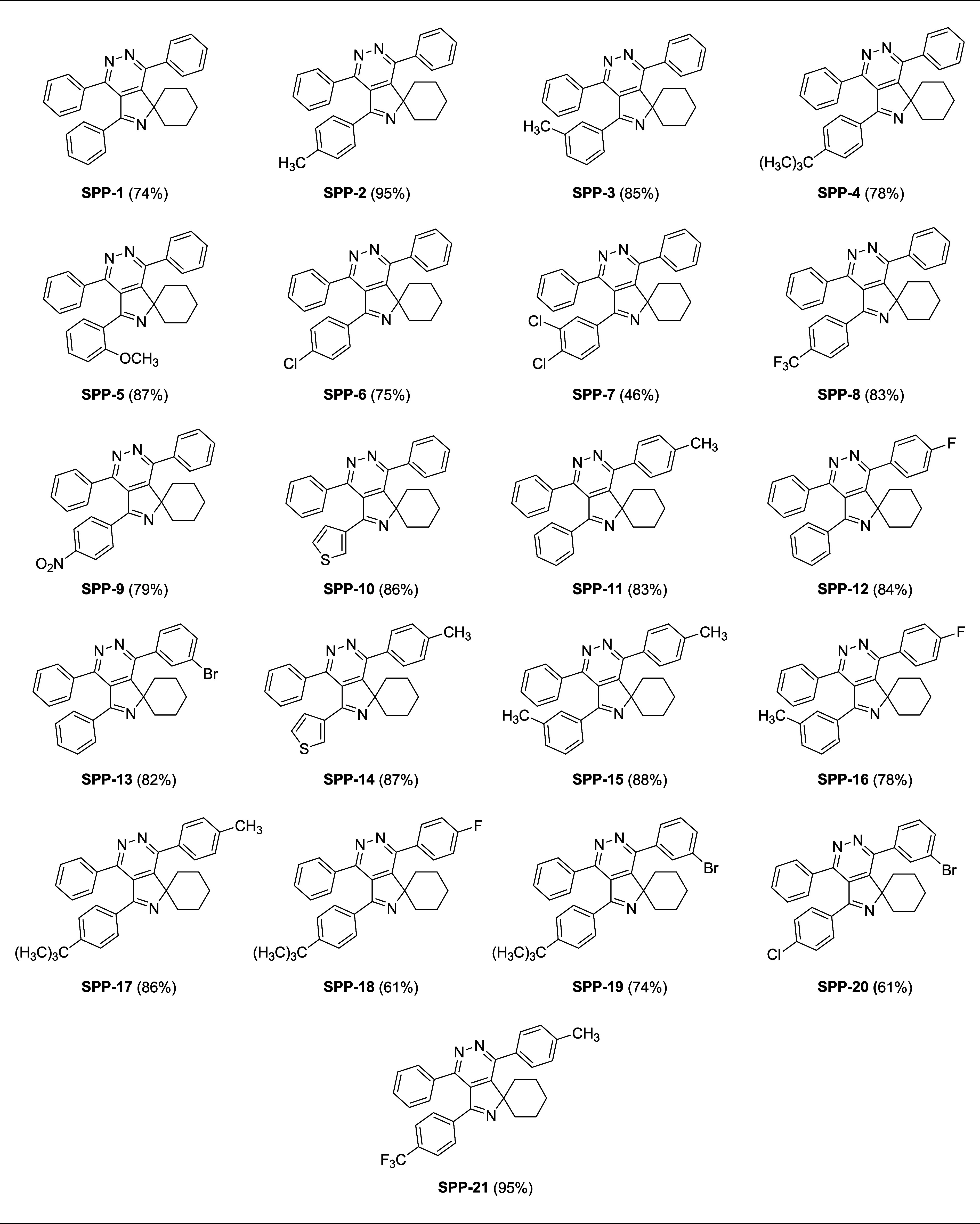
Scope of the Synthesis of Spiro-pyrrolopyridazines (SPPs)^a^

a Yields are of isolated products.

## Materials and Methods

### Cell Culture and XTT Assay

Human breast cancer (MCF-7), human lung cancer (H69AR), and human prostate cancer (PC-3) human nontumorigenic HEK-293 cells were obtained from the Health Protection Agency (UK) and Interlab Cell Line Collection (ICLC, Italy). Cells were maintained in RPMI medium (Sigma) containing 10% heat-inactivated fetal bovine serum, 1% penicillin, and 1% l-glutamine in a 37° humidified CO_2_ incubator.

XTT (2,3-bis(2-methoxy-4-nitro-5-sulfophenyl)-2*H*-tetrazolium-5-carboxanilide) cell viability test was used to determine the cytotoxic activities of SPPs, which were dissolved in dimethyl sulfoxide (DMSO). Cells were seeded in 96-well plates at a density of 10^4^/well and treated with increasing concentrations of SPP (1–250 μM) for 24, 48, and 72 h. After the incubation period, 100 μL of XTT was added to all experimental wells, and 96-well plates were incubated at 37 °C for 4 h. After the color change was observed, the absorbance values of the wells were recorded at 570 nm in the microplate reader (Tecan). The IC_50_ values for all synthesized compounds, representing the concentration needed to inhibit 50% of cancer cell proliferation, were determined using Biosoft CalcuSyn 2.1 software.^13^ The following formula was used to calculate selectivity indexes (SIs), which represent the cytotoxic selectivity of SPPs against cancer cells and normal cells (HEK-293):


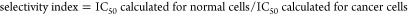


A value greater than or equal to 2 is considered an outstanding selectivity index (SI) in the literature.^14^

### Flow Cytometric Detection of Apoptosis

Annexin V, a protein exhibiting a strong affinity for phosphatidylserine (PS), a phospholipid typically localized in the inner layer of the plasma membrane, plays a significant role in apoptosis detection. During apoptosis, PS translocates from the inner to outer regions of the plasma membrane, making it a valuable marker for apoptosis identification. Annexin V specifically attaches to PS present on the surface of apoptotic cells. To differentiate between apoptotic and viable cells with intact membranes, a secondary dye such as phosphate iodide (PI) is employed. By assessing fluorescence patterns, viable cells (annexin V- and PI-) and apoptotic cells (annexin V+ and PI+) can be distinguished, providing insights into their respective percentages. We used FITC Annexin V Apoptosis Detection Kit I (BD Pharmingen).^15^ Briefly, cells were seeded at a density of 10^6^ cells per well in a six-well plate and treated with the most effective concentrations of SPPs for 72 h. Subsequently, cells were washed with cold PBS, resuspended in 1 mL of 1× Binding Buffer, and stained with 5 μL of Annexin V FITC and 5 μL of PI. After vortexing, the solution was incubated at room temperature (25 °C) for 15 min in the dark. At the end of the incubation, 400 μL of 1× Binding Buffer was added to each sample, and apoptosis was assessed using flow cytometry (BD Accuri C6 Flow Cytometer).

### Western Blot Analysis

For the extraction of total proteins, the M-PER Mammalian Protein Extraction Reagent from Thermo Fisher was utilized. Specifically, cells were treated with 250 μL of the extraction reagent and incubated at room temperature for 10 min with gentle shaking at 300 rpm. After this step, the cell suspension underwent centrifugation at 14,000*g* for 10 min, and the resulting supernatant was collected. Determination of the protein concentration was carried out using the Bradford method. Following this, protein separation was accomplished through SDS polyacrylamide gel electrophoresis, followed by transfer to nitrocellulose membranes. Post transfer, the membranes underwent blocking with 5% nonfat dry milk for 1 h and were subjected to three washes with Tris-buffered saline (TBS). Incubation with primary antibodies (Bcl-2: Anti-Bcl-2 antibody [ab196495, Abcam]; Bax: Anti-Bax antibody [ab216494, Abcam]; cytochrome c: Anticytochrome c antibody [ab4051]; EGFR: Anti-EGFR antibody [ab32077]) occurred overnight at 4 °C. β-Actin served as the loading control. After the washing steps, the membranes were exposed to secondary antibodies for 1 h at room temperature (1:1000 dilution). Visualization of protein bands was performed using the Kodak Gel Logic 1500 imaging system, and quantification was carried out using ImageJ software.^16^

### EGFR Kinase Inhibitory Assay

The inhibitory effects of the target compounds on various EGFR kinases were evaluated through a well-established homogeneous time-resolved fluorescence (HTRF) method-based assay, utilizing the HTRF KinEASE-TK kit (cat #62TK0PEC, Cisbio).^17^ SPP10 underwent screening at progressively diluted concentrations in the presence of 1% dimethyl sulfoxide (DMSO), preceded by a 5 min preincubation of the kinase and compounds. Initiation of all reactions occurred by adding ATP and TK-substrate-biotin, followed by a 60 min incubation at room temperature and subsequent quenching with a stop buffer containing 62.5 nM Strep-XL665 and TK antibody (Ab)-Cryptate. Following a 1 h plate incubation, readings were taken on a microplate reader using standard HTRF settings. IC_50_ values were determined using GraphPad Prism 5.0 software. Each reaction was carried out in duplicate, and a minimum of three independent determinations were performed. The data were analyzed using GraphPad Prism.

### Molecular Docking and admetSAR Analysis

For SPP10, molecular docking studies were carried out against the ATP binding sites of the EGFR tyrosine kinase wild type (EGFRWT) and the EGFR tyrosine mutant (EGFRT790M) via Autodock Vina 4.2.5.1 software. The X-ray crystallographic structures of the EGFR proteins (EGFRWT PDB ID: 4HJO; EGFRT790M PDB ID: 3W2O) were obtained from the protein databank RCSB (https://www.rcsb.org/) in PDB format.

The Protein Preparation Wizard was employed to prepare the proteins, including assigning preprocessed bond orders, adding hydrogens, treating metals, and deleting water molecules. Energy minimization was conducted with an RMSD of 0.30 Å. The 3D diagrams of the ligands were created using Maestro 8.5, a component of Schrödinger’s suite. Open Babel software was utilized to generate the 3D structures of the synthesized compounds. The prework orientation aimed to achieve a grid box parameter with the lowest RMSD value below 2 Å. The grid center EGFRWT was set as *X* = 21.41, *Y* = 3.62, and *Z* = 21.94 with dimensions of the grid box of 60 Å × 60 Å × 60 Å. Following calibration and optimization, the same grid box size and other parameters were consistently applied for EGFRT790M. The complete setup was executed to generate diverse docked conformations. Discovery Studio software was employed to visualize the secondary structures of the molecules.

The AdmetSAR 2.0 online tool (http://lmmd.ecust.edu.cn/admetsar2) was utilized to predict the absorption, distribution, metabolism, excretion, and toxicity characteristics of SPP10. This platform supplies data on various physicochemical properties, including molecular weight (MW), Log *P*_o/w_*c* (octanol–water partition coefficient), log *S* (solubility), log *K*_p_ (skin permeation), hydrogen bond acceptor (Hy-A), hydrogen bond donor (Hy-D), total polar surface area, and molar refractivity (M.ref). These parameters offer insights into the ADMET properties of any drug or organic molecule. When developing a molecule as a potential drug candidate, adherence to the Lipinski rule of five (Ro5) and other criteria is imperative. According to Ro5, ADME parameters reflect a compound’s accessibility within the body. Specifically, a molecular weight ≤500, hydrogen bond acceptor ≤10, hydrogen bond donor ≤5, Log *P* ≤ 5, and molar refractivity ≤140, satisfying the rule of five, and falling within the Log *P*_o/w_ range of −2 to 6.5, polar surface area range of 7 to 200, log *S* range above −4, and a drug score value above 0.5 are considered acceptable for the synthesized compounds.^18^

### Statistical Analysis

We utilized GraphPad Prism 5.0 software to perform statistical analysis, employing a one-way ANOVA test, followed by Tukey’s post-ANOVA test for multiple comparisons with a significance level set at *p* < 0.05. The results are presented as the mean ± SD.

## Results and Discussion

### Chemistry

As we reported previously,^10,19^ spiro-pyrrolopyridazine (SPP) derivatives investigated in this study were synthesized from α,β-alkynic ketones **1** in four steps as depicted in Scheme 1. The conjugate addition of 1-ethynylcyclohexylamine to α,β-alkynic ketones **1** in refluxing methanol yielded cyclohexane-embedded *N*-propargylic β-enaminones **2**. Then, Sonogashira cross-coupling of β-enaminones **2** with aryl iodides generated internal alkyne-tethered *N*-propargylic β-enaminones **3**.^20^ Subsequently, the reaction of β-enaminones **3** with a base produced spiro-2*H*-pyrrole (SP) derivatives via nucleophilic cyclization followed by benzylic C–H oxidation.^10,19^ Finally, the condensation reaction of spiro-2*H*-pyrroles (SPs) with hydrazine monohydrate in refluxing 1-butanol afforded spiro-pyrrolopyridazine (SPP) derivatives with a broad substrate scope and functional group tolerance (Scheme 1).^10^ By employing this strategy, 21 new derivatives of spiro-pyrrolopyridazines (SPPs) were synthesized, the structures and yields of which are given in Table 1 (for further details, see the Supporting Information).^10^

### SPPs Induced Cytotoxicity in Cancer Cells

Heterocyclic compounds play a crucial role in organic chemistry, owing to their essential functions across diverse fields.^10^ Especially, nitrogen-containing molecules have huge importance in organic chemistry. Among these, pyridazines attract great attention since they are found in many pharmacologically active compounds and natural products.^21^ Furthermore, according to the literature, pyridazine-containing molecules may be potential anticancer agents to target EGFR tyrosine kinase.^22^ Pyridazines are acknowledged as privileged structures in medicinal chemistry, primarily owing to their capacity to function as isosteric substitutes for phenyl and heteroaromatic groups.^23^ Additionally, although less studied, spiro compounds are also known to show very promising biological activities, such as anticancer agents. The fused systems and the spiro unit within these molecules contribute noteworthy properties. In our previous study, we synthesized novel fused SPP derivatives with different electron-withdrawing and electron-donating groups that are likely to have cytotoxic and apoptotic activities.^10^ In this study, we first investigated the possible cytotoxic effects of SPPs (1–21) on a panel of cancer cells for 24, 48, and 72 h. All of the SPPs tested showed strong cytotoxic effects on breast, lung, and prostate cancer cells in a time- and concentration-dependent manner (data not shown). DMSO was used as a solvent control and showed no cytotoxic effects at any concentration. The highest cytotoxic effect was observed after 72 h, which is the longest treatment; therefore, IC_50_ values were calculated for this period. As shown in Table 2, IC_50_ values of SPPs on human cancer cells were between 2.31 and 140 μM. While MCF-7 breast cancer cells were the most sensitive cancer cell group to SPPs, the most resistant cell group was lung cancer cells. SPP10 was the most effective SPP on MCF-7 breast cancer cells with an IC_50_ value of 2.31 ± 0.3 μM, while the least cytotoxic SPP was SPP6 with an IC_50_ value of 48.17 ± 1.4 μM. In H69AR lung cancer cells, SPP12 was the most effective one with an IC_50_ value of 19.18 ± 0.4 μM and SPP20 was the least cytotoxic SPP with an IC_50_ value of 140.0 ± 2.4 μM. SPP15 was the most cytotoxic SPP on PC-3 cells with an IC_50_ value of 2.8 ± 0.4 μM, while SPP13 was the least cytotoxic SPP on PC-3 cells (IC_50_ = 89.7 ± 2.4 μM). Following MTT assays and IC_50_ calculations, it was observed that SPP3 and SPP10 exhibited the highest cytotoxic effect across all tested cancer cell lines, similar to the results for the reference drugs erlotinib (EB) and cisplatin (CP).

**Table 2:** IC_50_ Values (μM) of SPPs (1–21) on Human Cancer Cells and Nontumorigenic HEK-293 Cells^a^

**SPP**	**HEK-293 (nontumorigenic)**	**MCF-7 (breast cancer)**	**H69AR (lung cancer)**	**PC-3 (prostate cancer)**
SPP1	16.5 ± 1.2	21.3 ± 0.4	27.1 ± 0.8	35.4 ± 1.6
SPP2	37.7 ± 0.2	24.6 ± 0.3	18.7 ± 1.4	44.2 ± 2.8
SPP3	7.5 ± 1.0	10.4 ± 1.5	9.8 ± 0.7	8.3 ± 0.6
SPP4	55.3 ± 3.6	67.1 ± 0.2	92.0 ± 3.2	85.5 ± 1.2
SPP5	45.4 ± 2.0	40.9 ± 1.8	62.7 ± 0.7	70.6 ± 2.4
SPP6	77.8 ± 1.7	48.17 ± 1.4	137.0 ± 1.6	35.6 ± 0.4
SPP7	55.8 ± 0.6	69.2 ± 1.2	72.2 ± 3.4	21.1 ± 2.1
SPP8	38.2 ± 1.3	62.2 ± 3.5	71.9 ± 1.8	35.5 ± 0.7
SPP9	47.5 ± 2.6	57.4 ± 2.8	62.1 ± 1.2	89.2 ± 2.5
SPP10^*a*^	26.8 ± 0.4	2.31 ± 0.3	3.16 ± 0.8	4.2 ± 0.2
SPP11	87.7 ± 1.6	95.2 ± 2.4	75.7 ± 3.5	80.5 ± 2.3
SPP12	40.8 ± 2.2	12.6 ± 2.6	19.18 ± 0.4	24.2 ± 0.8
SPP13	52.7 ± 1.4	66.4 ± 1.2	70.8 ± 1.3	89.7 ± 2.4
SPP14	12.4 ± 0.2	41.5 ± 0.8	55.3 ± 1.6	61.4 ± 0.5
SPP15	24.1 ± 1.8	29.7 ± 2.2	35.2 ± 3.2	2.8 ± 0.4
SPP16	64.4 ± 2.3	58.3 ± 0.4	63.9 ± 2.8	76.3 ± 0.2
SPP17	74.6 ± 0.7	61.9 ± 2.6	63.3 ± 1.4	88.2 ± 3.5
SPP18	58.5 ± 1.3	65.8 ± 1.4	77.4 ± 1.7	59.6 ± 1.4
SPP19	33.4 ± 1.6	38.4 ± 0.7	34.9 ± 2.1	61.0 ± 2.2
SPP20	18.3 ± 2.1	47.6 ± 2.0	140.0 ± 2.4	24.2 ± 0.8
SPP21	47.1 ± 0.4	54.7 ± 2.9	21.8 ± 0.6	42.7 ± 1.6
erlotinib	14.7 ± 2.8	19.4 ± 2.0	21.5 ± 1.8	18.9 ± 3.2
cisplatin	16.4 ± 0.4	16.8 ± 2.4	18.2 ± 0.8	14.9 ± 4.1

a SPP10, which has a specific cytotoxic effect against all tested cancer cells.

### Selective Cytotoxic Effect of SPP10

To determine the cytotoxic selectivity of the most effective SPPs against cancer cells, the selectivity index (SI) was calculated for SPP3 and SPP10. Of these, SPP3 was not evaluated as a cancer-specific compound because it showed a high cytotoxic effect (7.5 ± 1.0 μM) in nontumorigenic HEK-293 cells and SI values were 0.72, 0.76, and 0.90 for MCF-7, H69AR, and PC-3 cells, respectively. However, the cytotoxic activity of SPP10 was relatively low on nontumorigenic cells with the IC_50_ value of 26.8 ± 0.4 μM and calculated SI values were 11.6, 8.48, and 6.38 for MCF-7, H69AR, and PC-3 cells, respectively. According to these results, SPP10 was considered selectively cytotoxic to cancer cells. The presence of thienyl and phenyl groups on the pyridazine ring in SPP10 may contribute to its cytotoxicity. Thiophenes are known to exhibit excellent antibacterial, anti-inflammatory, antiviral, and anticancer properties. Ghorab et al. found that thiophene-containing compounds show a promising antibreast cancer activity, even higher than doxorubicin.^24^ de Oliveira and co-workers displayed that thiophene derivatives exhibit antiproliferative and antitumor activity on different cancer cell lines, in which an increase in the activity by the presence of an aromatic ring in addition to the thienyl group was also suggested.^25^ In another study, Othman et al. synthesized thiophene hybrid compounds and studied them on four human cancer cell lines (HepG2, HeLa, MCF-7, and HCT-116) for in vitro cytotoxic activity.^26^ It was found that two of the hybrids showed selective activity against HeLa human cervical cancer cells and MCF-7 breast cancer cells. Bachollet et al. synthesized 3,6-disubstituted 1,2-pyridazine derivatives and they reported that these derivatives possess excellent antiproliferative activity toward human cancer cell lines from several cancer types such as colon and lung cancers.^27^ George et al. investigated the cytotoxic activities of some pyrazoline derivatives bearing phenyl pyridazine against lung, colon, breast, and liver cancer cell lines. It was shown that among 21 derivatives, compound **8k** showed promising cytotoxic activity against all cancer cells as compared to the reference drug doxorubicin.^28^ In another study, compound **5a**, which carries a phenyl group from the synthesized indolyl pyrrole derivatives, has been shown to have stronger cytotoxicity than the drug doxorubicin.^29^ Given that a selectivity index (SI) value equal to or greater than 2 is deemed exceptionally high, additional experiments were conducted with SPP10, specifically focusing on instances in which the SI value was ≥2.

### Induction of Apoptotic Cell Death with SPP10

**Figure 1:**
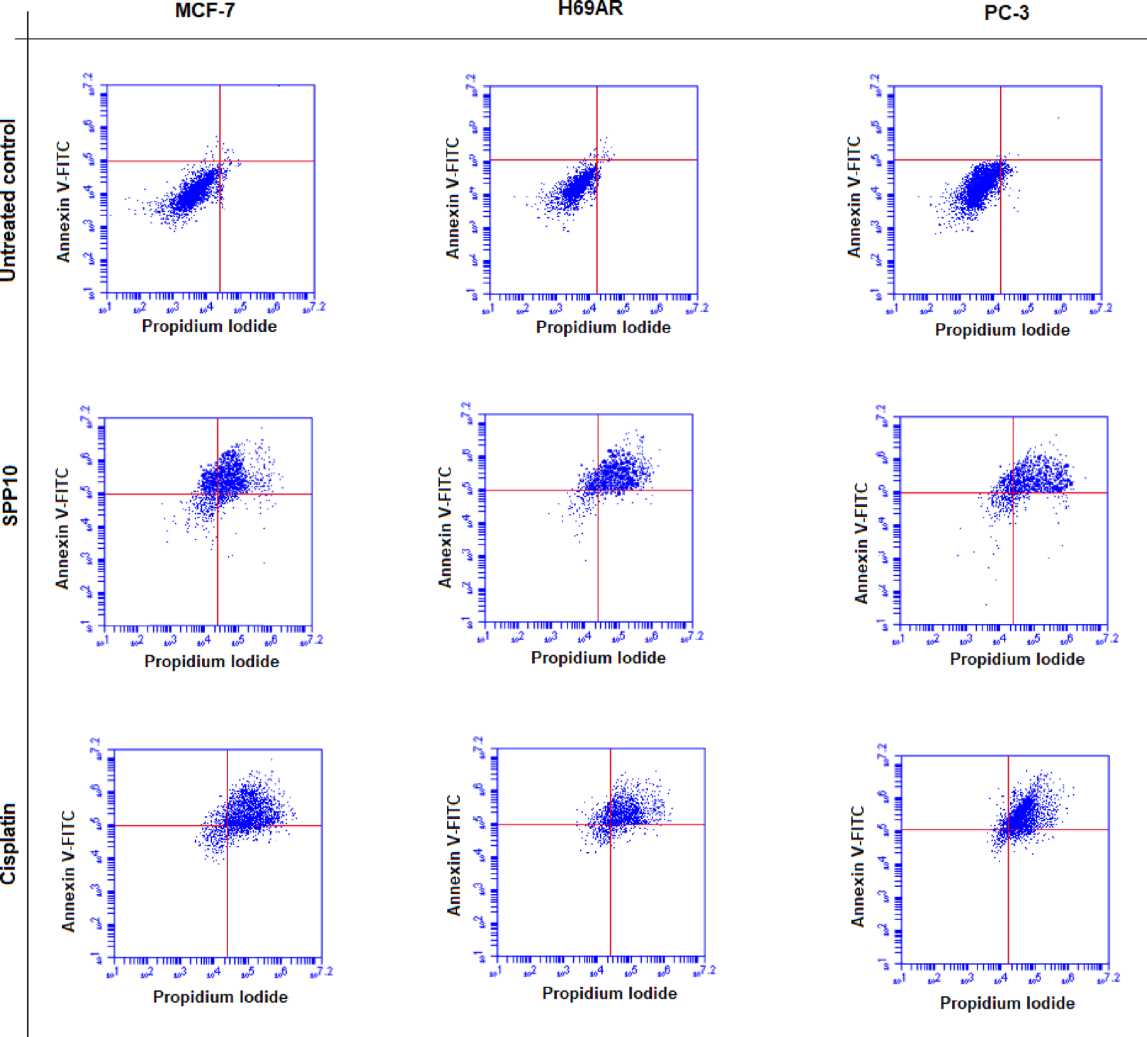
Percentages of apoptotic cells detected in MCF-7, H69AR, and PC-3 cancer cells after SPP10 and reference drug cisplatin treatment by flow cytometry analysis at 72 h.

One chemotherapeutic strategy for treating cancer has been to target apoptosis. Distinctive features linked to apoptotic cell death encompass nuclear fragmentation and cellular reduction. The apoptotic process involves two primary pathways, extrinsic and intrinsic, embedded within mitochondrial pathways.^30^ The assessment of apoptosis is commonly conducted in conjunction with the suppression of cellular growth as part of the biological response to treatment by using diverse chemotherapeutic agents. Therefore, after determining the IC_50_ values of SPP10, induction of apoptosis in all cancer cells was investigated via flow cytometry. Cancer cells were treated with the IC_50_ values of SPP10 and analyzed. In MCF-7 breast cancer cells, the percentage of early apoptotic cells was 28.7%, and the percentage of late apoptotic cells was 59.3% (Figure 1). In H69AR lung cancer cells, the percentages of early and late apoptotic cells were 25.6 and 61.2%, respectively (Figure 1). The percentage of early apoptotic cells in PC-3 prostate cancer cells was 21.8%, and the percentage of late apoptotic cells was 64.9% (Figure 1). These results showed that SPP10 strongly induces apoptotic cell death in all tested cancer types.

### Analysis of Proteins Involved in the Induction of Apoptosis

**Figure 2:**
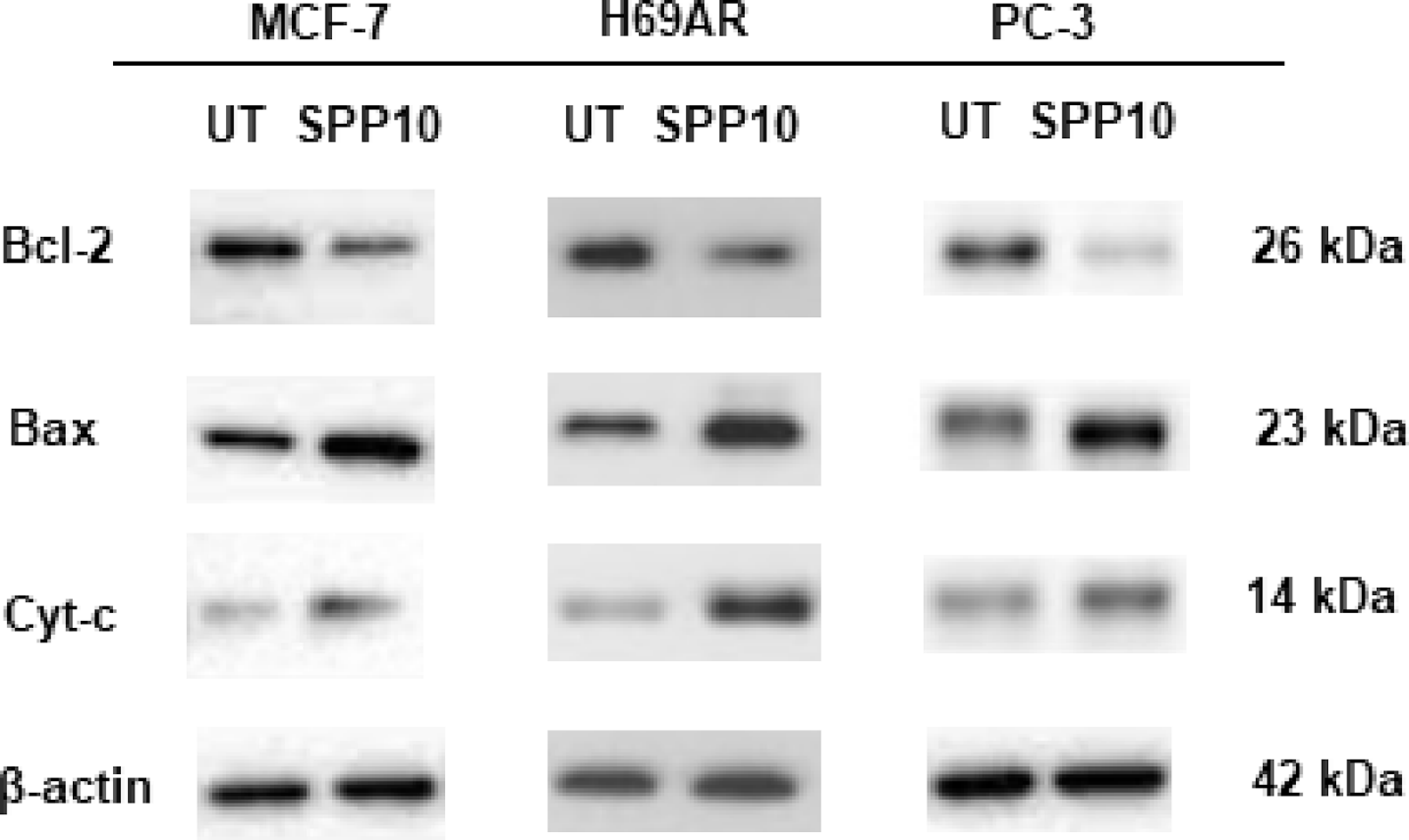
Bcl-2, Bax, and cytochrome c protein levels after treatment with the IC_50_ values of SPP10 in MCF-7, H69AR, and PC-3 cancer cells at 72 h (Cyt-c: cytochrome c; UT: untreated control).

To verify the induction of apoptotic cell death in cancer cells, proteins involved in apoptosis were analyzed via western blot analysis. Changes in the mitochondria constitute a significant pathway that oversees the regulation of Bcl family proteins and caspase-independent apoptosis. Bcl-2 and Bax, both representative proteins of the Bcl family with opposing roles in apoptosis, assume crucial functions in governing mitochondrial membrane permeability, mitochondrial function, and the release of cytochrome.^31,32^ As shown in Figure 2, after treatment with SPP10 for 72 h, the levels of antiapoptotic Bcl-2 protein were inhibited by 2.3-, 2.6-, and 3.2-fold in MCF-7, H69AR, and PC-3 cells, respectively. Levels of pro-apoptotic protein Bax were induced by 3.4-, 4.1- and 5.2- fold after SPP10 treatment in MCF-7, H69AR, and PC-3 cells, respectively (Figure 2). Cytochrome c levels were also induced by 3.8-, 3.6- and 4.4- fold in MCF-7, H69AR, and PC-3 cells, respectively (Figure 2). These results showed that SPP10 is a potent inducer of apoptosis in all cancer cell lines and causes significant changes in proteins vital for the apoptotic process.

### EGFR Kinase Inhibitory Effect of SPP10

**Figure 3:**
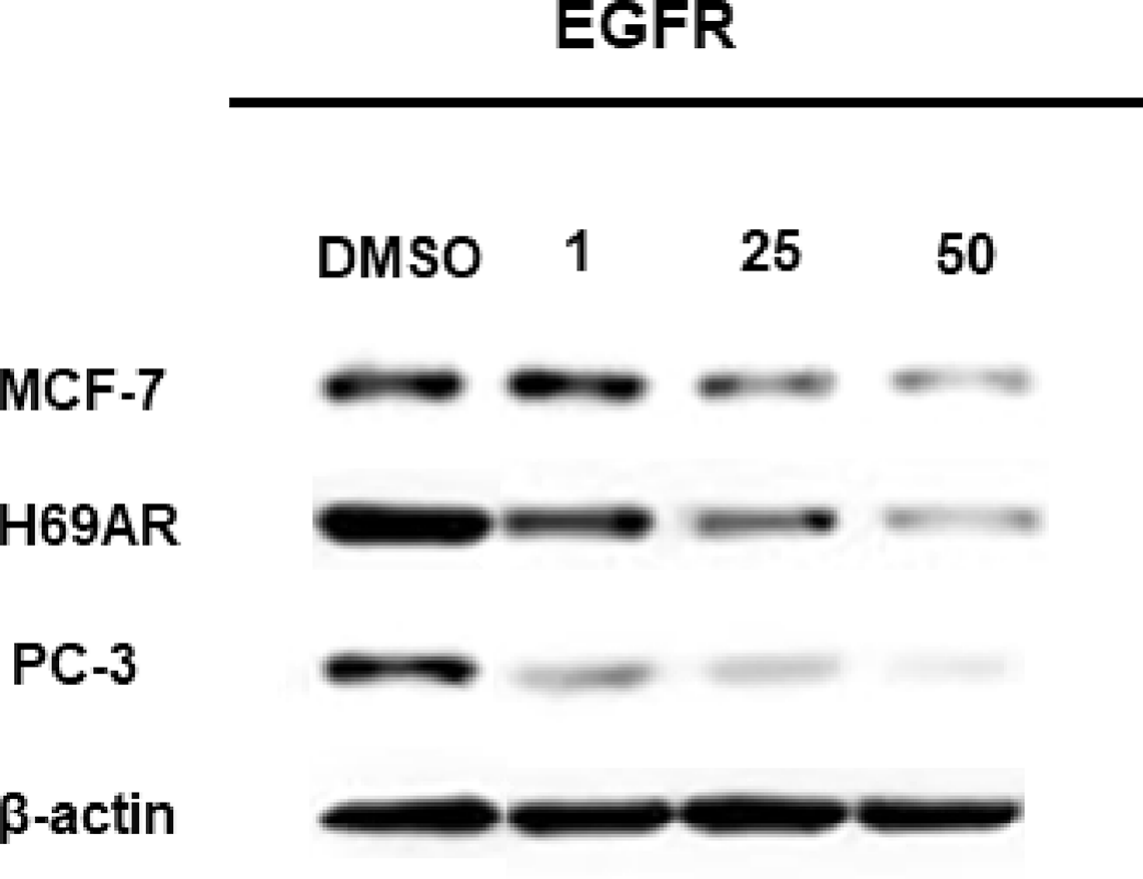
Concentration-dependent inhibition of EGFR protein by SPP10 in MCF-7, H69AR, and PC-3 cancer cells at 72 h.

Pyridazines with dipole moments are known to have a significant capacity to interact with cellular targets.^33^ Therefore, in this study, in addition to the cytotoxic and apoptotic effects of the synthesized SPPs, their interactions with epidermal growth factor receptor (EGFR), an important therapeutic target for cancer cells, were also investigated via a homogeneous time-resolved fluorescence (HTRF) assay. The findings demonstrated that SPP10 displayed EGFR kinase inhibitor activity with IC_50_ values varying from 0.20 to 0.42 μM in cancer cells. SPP10 exhibited activity similar to that of EB with IC_50_ values of 0.40 ± 0.12 and 0.42 ± 0.8 μM in MCF-7 and PC-3 cells, respectively. SPP10 exerted greater potency with an IC_50_ value of 0.20 μM against EGFR compared to EB in H69AR cells (IC_50_ = 0.39 ± 0.1 μM). SPP10 exhibited similar EGFRT790M kinase inhibitory activity to EB with IC_50_ values of 0.37 ± 0.2, 0.25 ± 0.7, and 0.39 ± 0.2 μM in MCF-7, H69AR, and PC-3 cells, respectively. As shown in Figure 3, western blot analysis confirmed the concentration-dependent inhibition of EGFRWT by SPP10 at 72 h in all of the tested cancer cell lines.

### Docking SPP10 with EGFR Tyrosine Kinases

**Figure 4:**
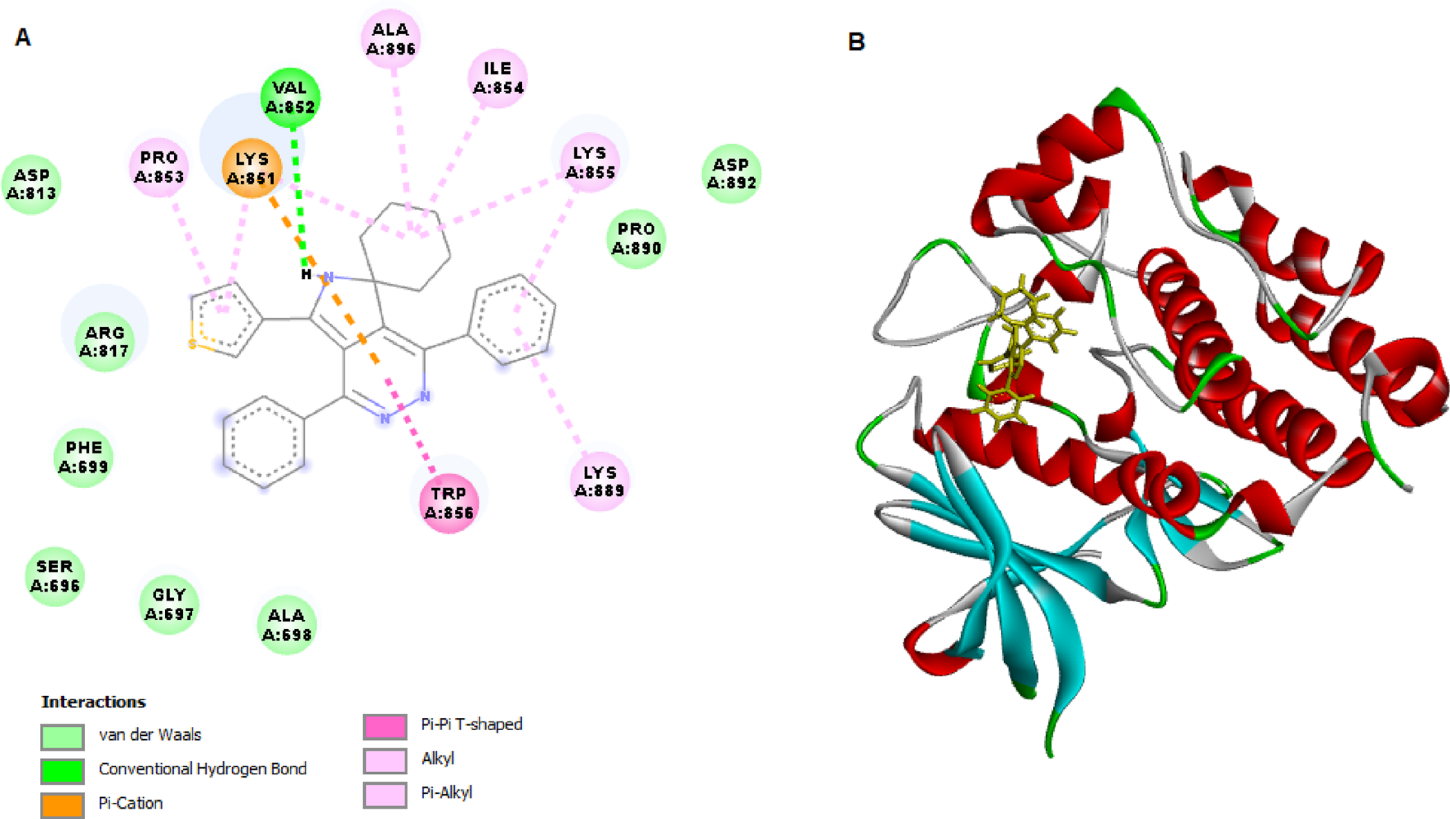
2D (A) and 3D (B) molecular interactions of SPP10 with EGFRWT.

**Figure 5:**
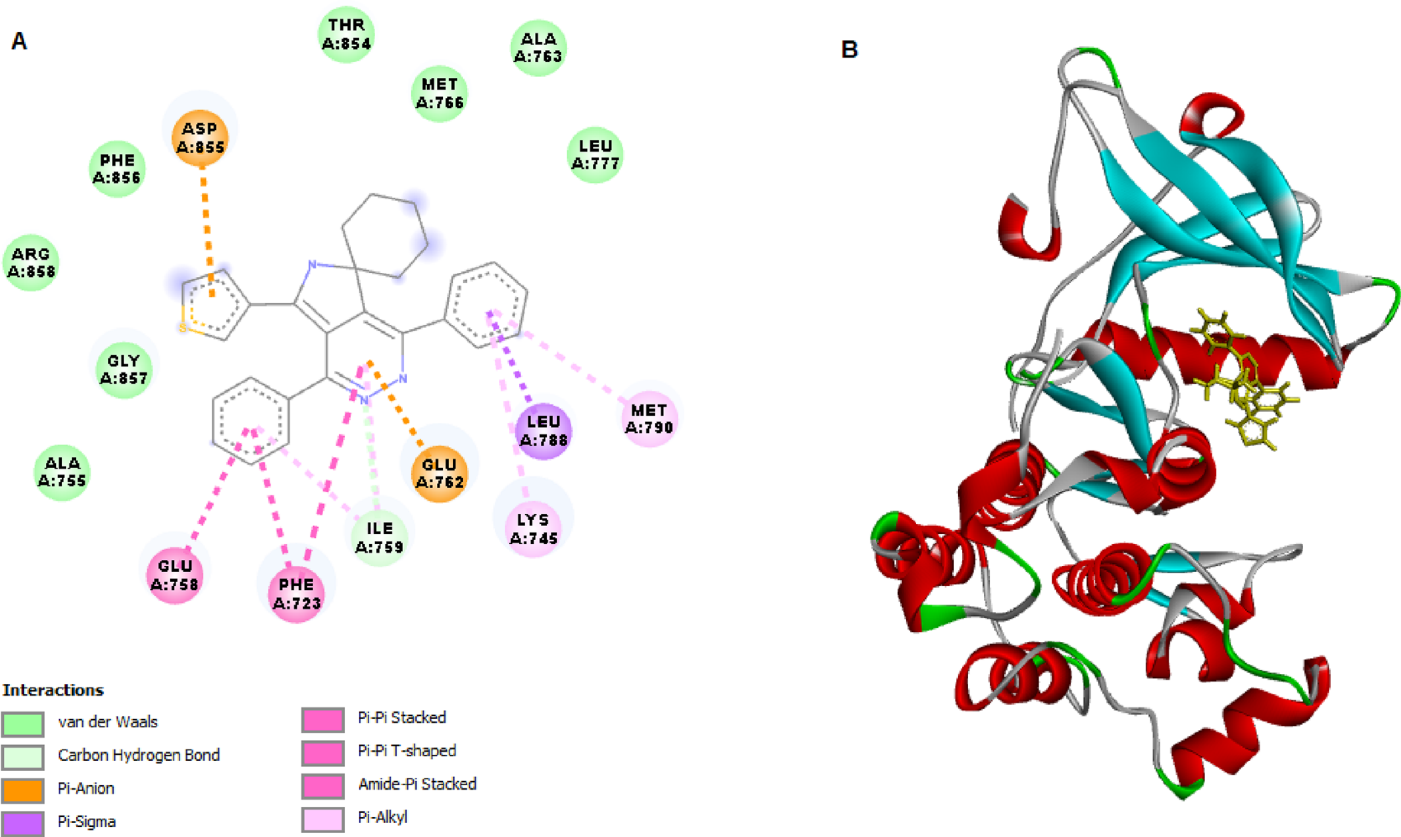
2D (A) and 3D (B) molecular interactions of SPP10 with EGFRT790M.

For in silico molecular docking, analysis was conducted for SPP10 against EGFRWT and EGFRT790M (Figures 4 and 5). The validation of SPP10 ligands was conducted based on their binding affinities with the respective receptor targets. Table 3 illustrates the docking scores (kcal/mol), hydrogen bond interactions, bond lengths, and amino acids participating in these interactions.

**Table 3:** Molecular Docking Analysis of SPP10 with EGFRWT and EGFRT790M

		**EGFRWT**			**EGFRT790M**	
	**docking score** (kcal/mol)	**H bond interaction**	**other interaction**	**docking score** (kcal/mol)	**H bond interaction**	**other interaction**
SPP10	–152.468	Lys851, Arg817	Ala 698, Asp831, Arg817, Pro853	–134.866		Glu758, Phe723, Asp855, Glu762, Asp855, Glu762, Met790, Lys745
EB	–179.941	Ala698, Phe699, Arg817, Asn818, Tyr867	Phe699, Leu838, Ala835, Ala 840.	**-**150.700	Lys745	Met790, Met766, Leu844, Leu718, Leu792

EGFR is frequently modified in human cancer through overexpression, amplification, and mutation, making it one of the most commonly affected genes.^34^ Specifically inhibiting EGFR activity hinders signal transduction pathways that regulate tumor cell growth, proliferation, and resistance to apoptosis. Clinical treatments for various malignancies often involve small-molecule tyrosine kinase inhibitors and monoclonal antibodies, widely recognized as common agents targeting EGFR.

The binding energy of SPP10 was similar to that of the original EGFR ligand EB and was in line with the EGFR kinase inhibitory assay results. SPP10 exhibited a strong binding energy of −152.468 kcal/mol, indicating robust interactions with EGFRWT. SPP10 interacted with the binding site of EGFRWT via two hydrogen bonds with Lys851 and Arg817 and formed pi–donor hydrogen bonds with EGFRWT via Ala698 (Figure 4). Steric interactions were formed with Asp831, Arg817, and Pro853 by interacting with EGFRWT by SPP10. Molecular docking analysis with EGFRT790M was also conducted to find the interaction level of SPP10 with mutant EGFR tyrosine kinases. SPP10 showed a strong binding affinity (−134.866 kcal/mol) with mutant EGFRT790M. SPP10 did not form a hydrogen bond but instead formed some pi-shaped linkages via Glu758 and Phe723. SPP10 also established pi–anion linkages with Asp855 and Glu762, pi–sigma linkages with Leu788, and pi–alkyl linkages with Met790 and Lys745 (Figure 5).

**Figure 6:**
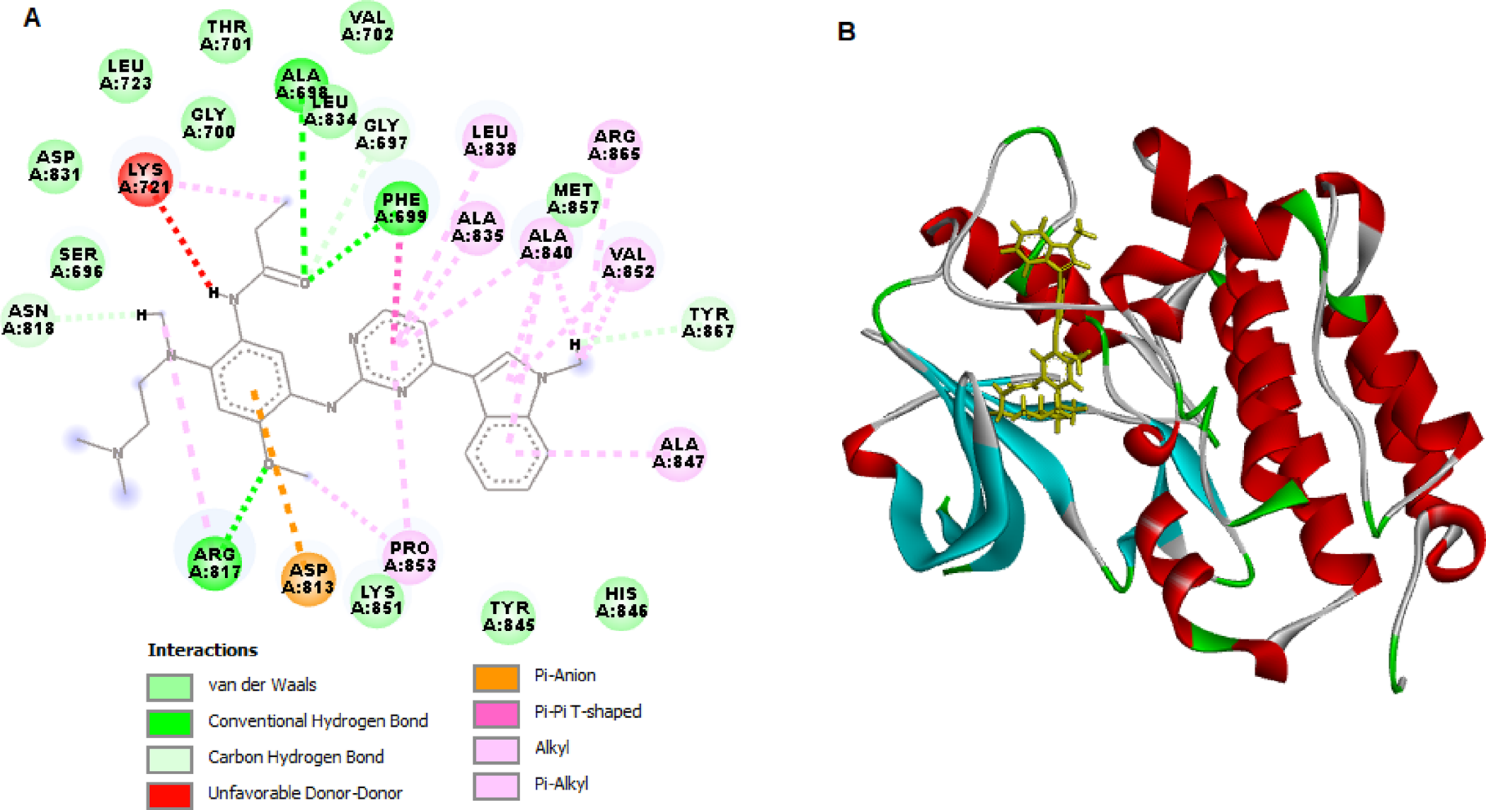
2D (A) and 3D (B) molecular interactions of EB with EGFRWT.

**Figure 7:**
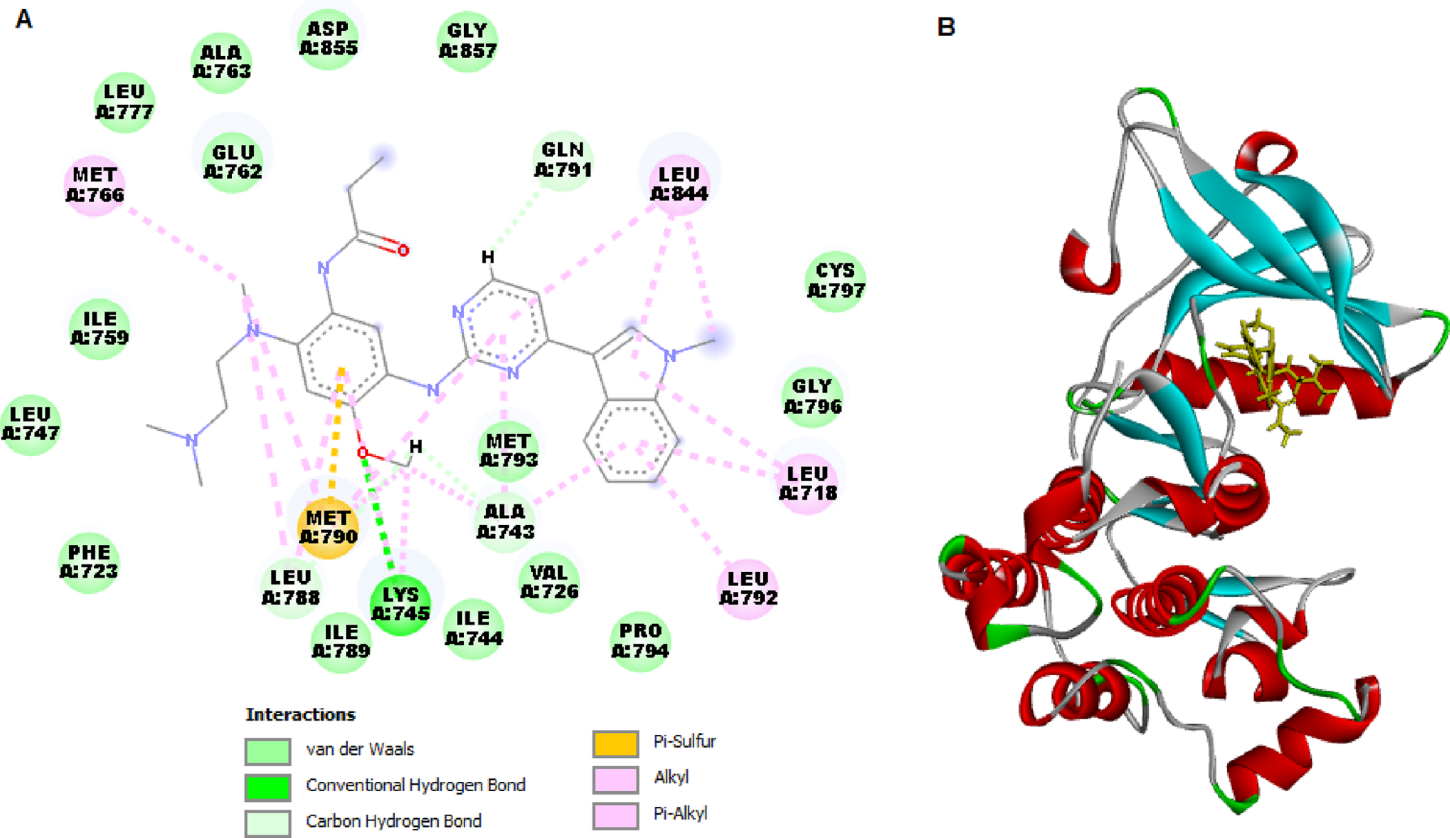
2D (A) and 3D (B) molecular interactions of EB with EGFRT790M.

The results of the docking protocol were validated by redocking the cocrystallized ligand (EB) into the active sites of EGFRWT. The root-mean-square deviation (RMSD) of EB was 1.2, confirming the validity of the docking process. The binding energy of EB with EGFRWT was −179.941 kcal/mol. EB interacts with the binding site of EGFRWT via hydrogen bonds with Ala698, Phe699, Arg817, Asn818, and Tyr867. Pi-shaped linkages with the pyridazine ring of EB were established via Phe699, and pi–alkyl linkages were formed via Leu838, Ala835, and Ala 840 (Figure 6). Interaction of EB was also strong with a binding energy of −150.700 kcal/mol. EB interacts with the binding site of EGFRT790M via Lys745. Pi–sulfur linkage with EB was established via Met790. Alkyl linkages were formed via Met766 and Leu792, and pi–alkyl linkages were formed via Leu844 and Leu718 (Figure 7).

### ADMET Analysis of SPP10

The ADMETlab 2.0 online tool assesses the physicochemical properties of potential drug candidates based on Lipinski’s five rules. According to ADME predictions, drug candidates should adhere to Ro5, with 0 to 1 violation. In our analysis, the molecular weight of SPP10 was 421.160, and the numbers of hydrogen bond acceptors and donors were 3 and 0, respectively, which complied with Ro5. The logarithm of the *n*-octanol/water partition coefficient (Log *P*_o/w_) indicates lipophilicity, crucial for transport mechanisms such as membrane permeability. The Log *P*_o/w_ value of SPP10 is 4.08, suggesting moderate permeability. The total polar surface area (TPSA) reflects the sum of polar atoms, primarily oxygen and nitrogen, with higher TPSA values indicating nonpermeability. SPP10 had a TPSA value of 38.140, suggesting potential permeability. TPSA was used to calculate the percentage of absorption (%ABS), which was 99% for SPP10, indicating good cellular membrane permeability. These results indicated that SPP10 satisfied Ro5: MW ≤ 500 Da, Log *P* < 5, nHBD ≤ 5, nHBA ≤ 10, and TPSA < 140 Å. In addition, the value of Log *S* for SPP10 is −6.446, which indicates moderate solubility in water. The human intestinal absorption value was 0.003, and 20 and 30% bioavailability values were more than 20 and 30%, respectively, indicating good bioavailability. admetSAR analysis revealed favorable lipophilicity, low fraction unbound, and appropriate dispersion, indicating high bioavailability for SPP10. Pyridazines are known to improve the physicochemical properties of drug molecules by increasing their water solubility due to their ability to act as hydrogen bond acceptors.^30^ The rat oral toxicity value of SPP10 was 0.108, indicating low acute oral toxicity.

## Conclusions

The findings presented in this study shed light on the potential of SPP derivatives, with SPP10 standing out as a particularly promising candidate. The demonstrated potency of SPP10 in inducing cytotoxicity across breast, lung, and prostate cancer cells, coupled with its selective impact on cancer cells, underscores its potential as an effective anticancer agent. The ability of SPP10 to induce apoptotic cell death, as evidenced by alterations in key apoptotic proteins, adds to its appeal. Moreover, the compound exhibits notable EGFR kinase inhibitory activity, highlighting its potential as a targeted therapy for cancer. The comprehensive in vitro assessments, including cytotoxicity, apoptosis induction, and molecular interactions, position SPP10 as a significant contender for further investigations in the realm of cancer treatment. The study not only contributes valuable insights into the development of novel anticancer agents but also emphasizes the importance of heterocyclic compounds, particularly spiro-class heterocyclic molecules, in advancing cancer research and therapeutic strategies.

## Supporting Information Available

Experimental procedures, spectroscopic data, and copies of ^1^H and ^13^C NMR spectra for all spiro-pyrrolopyridazine (SPP) derivatives (PDF)
