# Supplementary material for: Evaluation of Novel Spiro-pyrrolopyridazine Derivatives as Anticancer Compounds: In Vitro Selective Cytotoxicity, Induction of Apoptosis, EGFR Inhibitory Activity, and Molecular Docking Analysis
Source: ACS Omega. 2024 May 20;9(22):23713–23. doi: 10.1021/acsomega.4c00794 (PMC11154717; doi:10.1021/acsomega.4c00794)
Supplement: Supplementary file 1 — ao4c00794_si_001.pdf [file ao4c00794_si_001.pdf]

SUPPORTING INFORMATION

for

Evaluation of Novel Spiro-Pyrrolopyridazine Derivatives as Anti-Cancer Compounds: In Vitro Selective Cytotoxicity, Induction of Apoptosis, EGFR Inhibitory Activity and Molecular Docking Analysis

Harika Atmaca,[1] Suleyman Ilhan,[1] Çisil Çamlı Pulat,[2] Buse Aysen Dundar,[3] Metin Zora[3,*]

[1]*Department of Biology, Faculty of Engineering and Natural Sciences, Manisa Celal Bayar University, 45140 Manisa, Turkey*

[2]*Applied Science Research Center, Manisa Celal Bayar University, 45140 Manisa, Turkey*

[3]*Department of Chemistry, Middle East Technical University, 06800 Ankara, Turkey*

Table of Contents

Experimental Section S2

General information S2

Scheme S1. Synthesis of spiro-pyrrolopyridazines (SPP) S3

Table S1. Scope of the synthesis of spiro-pyrrolopyridazines (SPP) S4

General Procedure for the synthesis of spiro-pyrrolopyridazines (SPP) S5

Copies of ^{1}H and ^{13}C NMR Spectra S16-S36

References S37

[*]Corresponding author. E-mail: zora@metu.edu.tr

Experimental Section

General information. ^{1}H and ^{13}C NMR spectra were recorded on a Bruker Spectrospin Avance DPX400 spectrometer at 400 and 100 MHz, respectively. Chemical shifts are given in parts per million (ppm) relative to tetramethylsilane (TMS). Coupling constants (*J*) are given in hertz (Hz), and spin multiplicities as follows: s (singlet), d (doublet), t (triplet), q (quartet), m (multiplet). DEPT ^{13}C NMR information is depicted in parentheses as C, CH, CH_2 and CH_3. IR data were obtained on a Bruker Alpha spectrophotometer by using attenuated total reflection (ATR). Band positions are recorded in reciprocal centimeters (cm^{-1}). High-resolution mass spectra (HRMS) were obtained on a Agilent 6224 TOF LC/MS spectrometer by using electrospray ionization (ESI) with Micro-Tof; *m/z* values are reported (for each measurement, the mass scale was recalibrated with sodium formate clusters, and samples were dissolved and measured in CH_3OH or CH_3CN. Melting points were determined with a Stuart SMP50 apparatus. Flash chromatography was performed using thick-walled glass columns and 'flash grade' silica gel (230–400 mesh). TLC was accomplished by using commercially prepared 0.25 mm silica gel and visualization was effected with a short-wavelength UV lamp (254 nm). The relative proportions of solvents in chromatography solvent mixtures refer to the volume/volume ratio. All commercially available reagents were used directly without purification unless otherwise stated. All solvents used in reactions and chromatography were distilled and/or dried properly for purity. The inert atmosphere was created by a slight positive pressure (ca. 0.1 psi) of argon. All glassware was oven-dried prior to use.

Scheme S1. Synthesis of spiro-pyrrolopyridazines (SPP).

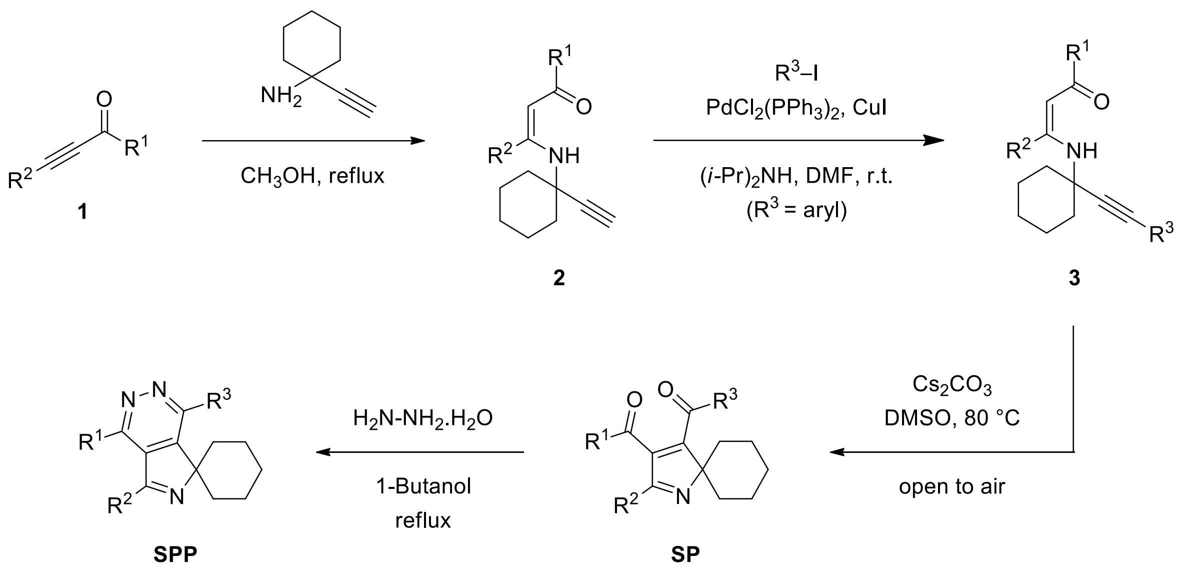

As shown in Scheme S1, the synthesis of spiro-pyrrolopyridazines (**SPP**) were accomplished from α,β-alkynic ketones **1** in four steps as we reported previously.[1,2] The reaction of α,β-alkynic ketones **1** with 1-ethynylcyclohexylamine in refluxing methanol yielded cyclohexane-embedded *N*-propargylic β-enaminones **2**. Then Sonogashira cross coupling of β-enaminones **2** with aryl iodides produced internal alkyne-tethered *N*-propargylic β-enaminones **3**.[3] Subsequently, the reaction of β-enaminones **3** with base produced spiro-2*H*-pyrrole (**SP**) derivatives via nucleophilic cyclization followed by benzylic C–H oxidation. At last, the condensation reaction of spiro-2*H*-pyrroles (**SP**) with hydrazine monohydrate in refluxing 1-butanol afforded spiro-pyrrolopyridazine (**SPP**) derivatives with broad substrate scope and functional group tolerance (Scheme S1).[1] By utilizing this strategy, 21 new derivatives of spiro-pyrrolopyridazines (**SPP**) were prepared as depicted in Table S1.[1]

Table S1. Scope of the synthesis of spiro-pyrrolopyridazines (SPP).[a]

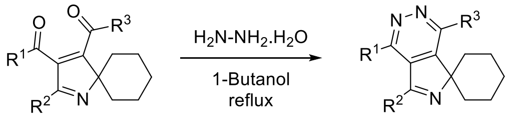

SP **SPP**

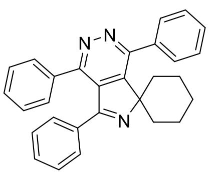

SPP-1 (74%, 2.5 h, 49.2 mg)

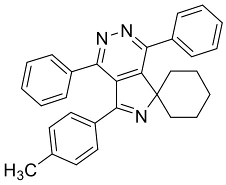

SPP-2 (95%, 4.0 h, 53.0 mg)

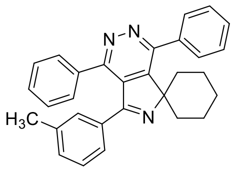

SPP-3 (85%, 3.0 h, 69.0 mg)

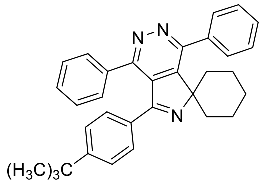

SPP-4 (78%, 2.5 h, 44.0 mg)

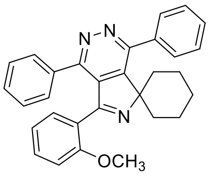

SPP-5 (87%, 2.5 h, 26.2 mg)

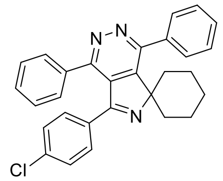

SPP-6 (75%, 3.0 h, 50.5 mg)

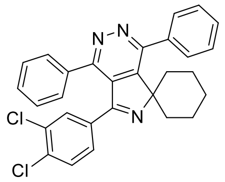

SPP-7 (46%, 2.0 h, 20.6 mg)

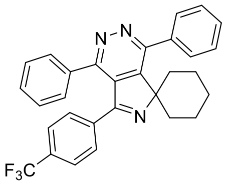

SPP-8 (83%, 3.0 h, 56.4 mg)

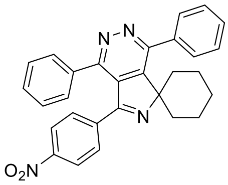

SPP-9 (79%, 3.0 h, 21.7 mg)

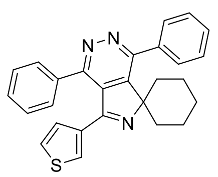

SPP-10 (86%, 3.5 h, 58.1 mg)

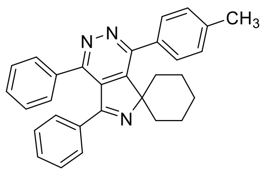

SPP-11 (83%, 2.0 h, 32.2 mg)

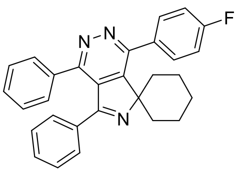

SPP-12 (84%, 3.0 h, 69.0 mg)

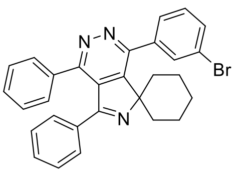

SPP-13 (82%, 4.0 h, 68.5 mg)

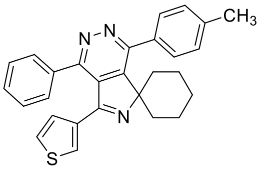

SPP-14 (87%, 4.0 h, 29.0 mg)

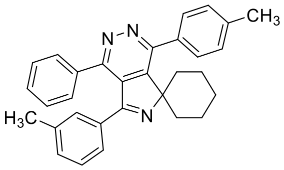

SPP-15 (88%, 4.0 h, 86.0 mg)

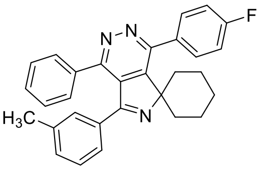

SPP-16 (78%, 6.0 h, 60.0 mg)

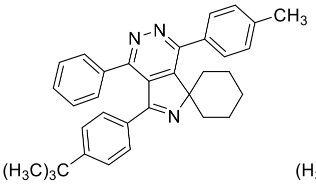

SPP-17 (86%, 3.5 h, 71.3 mg)

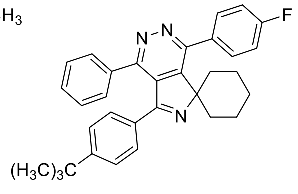

SPP-18 (61%, 4.0 h, 25.3 mg)

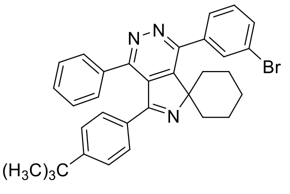

SPP-19 (74%, 2.0 h, 145.8 mg)

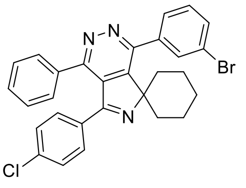

SPP-20 (61%, 5.0 h, 42.0 mg)

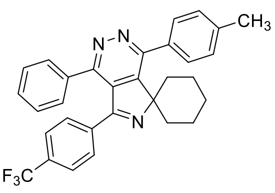

SPP-21 (95%, 5.0 h, 38.0 mg)

[a]Isolated yields, reaction times and amounts of the synthesized substances are given in parentheses.

General Procedure for the synthesis of spiro-pyrrolopyridazines (SPP) (Table S1). To a stirred solution of the corresponding spiro-2*H*-pyrrole (**SP**) (0.10 mmol) in 1-butanol (5 mL) was added $NH_2NH_2.H_2O$ (1.00 mmol), and the resulting mixture was heated to reflux, open to air, at 117 °C for 2.0–6.0 h as indicated in Table S1 (The progress of the reaction was monitored by routine TLC analysis for the disappearance of spiro-2*H*-pyrrole (**SP**) using hexane/ethyl acetate (4:1) as the eluent). After the reaction was over, 1-butanol was removed using a rotary evaporator, and ethyl acetate (30 mL) and distilled water (30 mL) were added. After the layers were separated, the aqueous phase was extracted with ethyl acetate (2 × 15 mL). The combined organic phases were dried over $MgSO_4$ and evaporated using a rotary evaporator to give a crude product, which was purified by flash chromatography on silica gel using hexane/ethyl acetate (9:1 followed by 4:1) as the eluent to afford the corresponding spiro-pyrrolopyridazine (**SPP**).

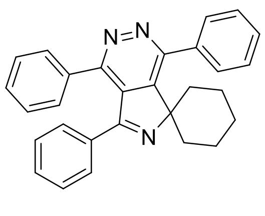

1',4',7'-Triphenylspiro[cyclohexane-1,5'-pyrrolo[3,4-*d*]pyridazine] (SPP-1). 1,3-Diphenyl-3-((1-(phenylethynyl)cyclohexyl)amino)prop-2-en-1-one (**SP-1**) (67.0 mg, 0.16 mmol) and $NH_2NH_2.H_2O$ (80.1 mg, 1.60 mmol) were employed to afford 49.2 mg (74%) of the indicated product as a white solid (R_f = 0.20 in 4:1 hexane/ethyl acetate; mp 194.7–196.0 °C. ^{1}H NMR (400 MHz, $CDCl_3$) δ 7.50–7.43 (m, 5H), 7.29–7.24 (m, 2H), 7.19–7.08 (m, 4H), 7.06–6.95 (m, 4H), 2.02–1.85 (m, 4H), 1.74 (d, *J* = 13.3 Hz, 1H), 1.57 (d, *J* = 12.5 Hz, 2H), 1.18 (d, *J* = 11.2 Hz, 2H), 1.07–0.95 (m, 1H); ^{13}C NMR (100 MHz, $CDCl_3$) δ 166.7 (C), 157.4 (C), 157.0 (C), 156.6 (C), 136.9 (C), 135.7 (C), 134.6 (C), 132.3 (C), 130.1 (CH), 129.6 (CH), 129.2 (CH), 129.1 (CH), 128.9 (CH), 128.32 (CH), 128.27 (CH), 127.78 (CH), 127.76 (CH), 80.4 (C), 34.2 (CH_2), 25.6 (CH_2), 23.4 (CH_2); IR (neat) 2933, 1584, 1445, 1381, 1282, 1091, 1078, 1023, 899, 852, 784, 761, 712, 695, 623 cm^{-1}; MS (ESI, *m/z*): 416.21 [M+H]$^+$; HRMS (ESI) calcd. for $C_{29}H_{26}N_3$: 416.2121 [M+H]$^+$, found: 416.2120.

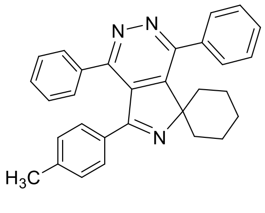

1',4'-Diphenyl-7'-(*p*-tolyl)spiro[cyclohexane-1,5'-pyrrolo[3,4-*d*]pyridazine] (SPP-2). (2-(*p*-Tolyl)-1-azaspiro[4.5]deca-1,3-diene-3,4-diyl)bis(phenylmethanone) (**SP-2**) (56.0 mg, 0.13 mmol) and $NH_2NH_2.H_2O$ (65.1 mg, 1.30 mmol) were employed to afford 53.0 mg (95%) of the indicated product as a light yellow solid (R_f = 0.22 in 4:1 hexane/ethyl acetate; mp 205.6–207.8 °C). ^{1}H NMR (400 MHz, $CDCl_3$) δ 7.62-7.54 (m, 5H), 7.41–7.37 (m, 2H), 7.32–7.27 (m, 1H), 7.17 (t, *J* = 7.5 Hz, 2H), 7.09 (d, *J* = 7.9 Hz, 2H), 6.91 (d, *J* = 7.6 Hz, 2H),

2.30 (s, 3H), 2.13–1.98 (m, 4H), 1.84 (d, *J* = 13.2 Hz, 1H), 1.67 (d, *J* = 12.6 Hz, 2H), 1.28 (d, *J* = 11.7 Hz, 2H), 1.11 (d, *J* = 12.9 Hz, 1H); ^{13}C NMR (100 MHz, $CDCl_3$) δ 166.6 (C), 157.3 (C), 157.0 (C), 156.6 (C), 139.1 (C), 136.9 (C), 135.8 (C), 132.4 (C), 131.7 (C), 130.2 (CH), 129.6 (CH), 129.1 (CH), 128.8 (CH), 128.4 (CH), 128.30 (CH), 128.25 (CH), 127.7 (CH), 80.2 (C), 34.2 (CH_2), 25.7 (CH_2), 23.4 (CH_2), 21.4 (CH_3); IR (neat) 2936, 1537, 1443, 1371, 1095, 1020, 897, 829, 816, 775, 761, 730, 699, 667, 624 cm^{-1}; MS (ESI, *m/z*): 430.23 $[M+H]^+$; HRMS (ESI) calcd. for $C_{30}H_{28}N_3$: 430.2278 $[M+H]^+$, found: 430.2271.

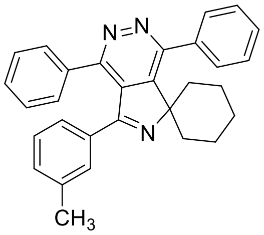

1',4'-Diphenyl-7'-(*m*-tolyl)spiro[cyclohexane-1,5'-pyrrolo[3,4-*d*]pyridazine] (SPP-3). (2-(*m*-Tolyl)-1-azaspiro[4.5]deca-1,3-diene-3,4-diyl)bis(phenylmethanone) (**SP-3**) (81.3 mg, 0.19 mmol) and $NH_2NH_2.H_2O$ (95.1 mg, 1.90 mmol) were employed to afford 69.0 mg (85%) of the indicated product as a light yellow solid (R_f = 0.14 in 4:1 hexane/ethyl acetate; mp 178.5–180.3 °C). ^{1}H NMR (400 MHz, $CDCl_3$) δ 7.64–7.52 (m, 5H), 7.39 (d, *J* = 8.1 Hz, 2H), 7.28 (dd, *J* = 8.1, 6.7 Hz, 1H), 7.20–6.99 (m, 5H), 6.91 (s, 1H), 2.10 (s, 3H), 2.05–1.96 (m, 3H), 1.84 (d, *J* = 12.2 Hz, 1H), 1.67 (d, *J* = 11.8 Hz, 2H), 1.29 (t, *J* = 10.2 Hz, 3H), 1.16–1.08 (m, 1H); ^{13}C NMR (100 MHz, $CDCl_3$) δ 166.7 (C), 157.3 (C), 156.9 (C), 156.6 (C), 137.4 (C), 136.9 (C), 135.8 (C), 134.3 (C), 132.4 (C), 130.0 (CH), 129.8 (CH), 129.6 (CH), 129.2 (CH), 129.1 (CH), 128.9 (CH), 128.3 (CH), 127.8 (CH), 127.6 (CH), 125.4 (CH), 80.3 (C), 34.2 (CH_2), 25.6 (CH_2), 23.3 (CH_2), 21.1 (CH_3); IR (neat) 2938, 1570, 1485, 1444, 1374, 1313, 1191, 1093, 1021, 939, 903, 853, 790, 777, 763, 724, 711, 694, 656, 623 cm^{-1}; MS (ESI, *m/z*): 430.23 $[M+H]^+$; HRMS (ESI) calcd. for $C_{30}H_{28}N_3$: 430.2278 $[M+H]^+$, found: 430.2278.

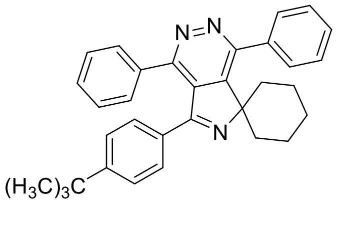

7'-(4-(*tert*-Butyl)phenyl)-1',4'-diphenylspiro[cyclohexane-1,5'-pyrrolo[3,4-*d*] pyridazine] (SPP-4). (2-(4-(*tert*-Butyl)phenyl)-1-azaspiro[4.5]deca-1,3-diene-3,4-diyl)bis (phenylmethanone) (**SP-4**) (58.3 mg, 0.12 mmol) and $NH_2NH_2.H_2O$ (60.1 mg, 1.20 mmol) were employed to afford 44.0 mg (78%) of the indicated product as a light yellow solid (R_f = 0.24 in 4:1 hexane/ethyl acetate; mp 238.9–240.1 °C). ^{1}H NMR (400 MHz, $CDCl_3$) δ 7.51–7.44 (m, 5H), 7.25–7.21 (m, 2H), 7.14 (t, *J* = 7.5 Hz, 1H), 7.05–6.95 (m, 6H), 2.02–1.88 (m, 4H), 1.74 (d, *J* = 13.1 Hz, 1H), 1.57 (d, *J* = 12.5 Hz, 2H), 1.23 (s, 2H), 1.18 (s, 9H), 1.00 (d, *J* = 12.9 Hz, 1H); ^{13}C NMR (100 MHz, $CDCl_3$) δ 166.8 (C), 157.4 (C), 156.9 (C), 156.7 (C), 152.2 (C), 136.9 (C), 135.6 (C), 132.6 (C), 131.7 (C), 130.1 (CH), 129.6 (CH), 129.2 (CH),

128.7 (CH), 128.3 (CH), 127.9 (CH), 127.7 (CH), 124.7 (CH), 80.4 (C), 34.7 (C), 34.2 (CH_2), 31.3 (CH_3), 25.7 (CH_2), 23.4 (CH_2); IR (neat) 2939, 1616, 1582, 1532, 1445, 1374, 1269, 1175, 1112, 1080, 1025, 989, 896, 839, 821, 764, 710, 694, 666, 622 cm^{-1}; MS (ESI, *m/z*): 472.27 $[M+H]^+$; HRMS (ESI) calcd. for $C_{33}H_{34}N_3$: 472.2747 $[M+H]^+$, found: 472.2748.

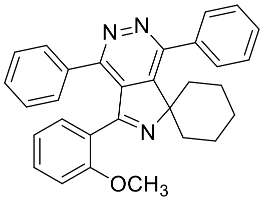

7'-(2-Methoxyphenyl)-1',4'-diphenylspiro[cyclohexane-1,5'-pyrrolo[3,4-*d*] pyridazine] (SPP-5). (2-(2-Methoxyphenyl)-1-azaspiro[4.5]deca-1,3-diene-3,4-diyl)bis (phenylmethanone) (**SP-5**) (30.5 mg, 0.07 mmol) and $NH_2NH_2.H_2O$ (34.0 mg, 0.68 mmol) were employed to afford 26.2 mg (87%) of the indicated product as a beige solid (R_f = 0.07 in 4:1 hexane/ethyl acetate; mp 217.5–218.7 °C). ^1H NMR (400 MHz, $CDCl_3$) δ 7.49 (tt, *J* = 5.0, 2.6 Hz, 5H), 7.42 (dd, *J* = 7.4, 1.7 Hz, 1H), 7.27 (d, *J* = 7.0 Hz, 2H), 7.18–7.09 (m, 2H), 7.01 (t, *J* = 7.5 Hz, 2H), 6.93 (t, *J* = 7.4 Hz, 1H), 6.20 (d, *J* = 8.3 Hz, 1H), 3.10 (s, 3H), 1.98 (d, *J* = 4.9 Hz, 3H), 1.84–1.69 (m, 2H), 1.58 (d, *J* = 39.8 Hz, 2H), 1.18 (t, *J* = 7.1 Hz, 3H); ^{13}C NMR (100 MHz, $CDCl_3$) δ 165.5 (C), 157.3 (C), 157.2 (C), 157.1 (C), 156.0 (C), 137.1 (C), 135.5 (C), 133.6 (C), 131.1 (C), 129.7 (CH), 129.6 (CH), 129.3 (CH), 129.1 (CH), 128.6 (CH), 128.3 (CH), 127.1 (CH),125.0 (CH), 120.6 (CH), 109.8 (CH), 80.3 (C), 60.5 (CH_3), 54.6 (CH_2), 25.7 (CH_2), 14.3 (CH_2); IR (neat) 2919, 1737, 1602, 1586, 1490, 1469, 1436, 1382, 1274, 1250, 1188, 1159, 1090, 1020, 991, 938, 890, 852, 763, 750, 706, 698, 667, 622 cm^{-1}; MS (ESI, *m/z*): 446.22 $[M+H]^+$; HRMS (ESI) calcd. for $C_{30}H_{28}N_3O$: 446.2227 $[M+H]^+$, found: 446.2226.

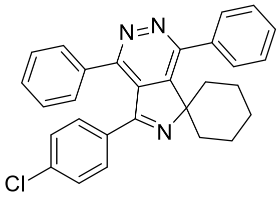

7'-(4-Chlorophenyl)-1',4'-diphenylspiro[cyclohexane-1,5'-pyrrolo[3,4-*d*]pyridazine] (SPP-6). (2-(4-Chlorophenyl)-1-azaspiro[4.5]deca-1,3-diene-3,4-diyl)bis(phenylmethanone) (**SP-6**) (70.0 mg, 0.15 mmol) and $NH_2NH_2.H_2O$ (75.1 mg, 1.50 mmol) were employed to afford 50.5 mg (75%) of the indicated product as a bright orange solid (R_f = 0.11 in 4:1 hexane/ethyl acetate; mp 253.4–256.1 °C). ^1H NMR (400 MHz, $CDCl_3$) δ 7.53–7.42 (m, 5H), 7.31–7.22 (m, 3H), 7.13 (t, *J* = 7.6 Hz, 2H), 7.02 (dd, *J* = 21.6, 8.6 Hz, 4H), 1.96–1.89 (m, 3H), 1.76 (d, *J* = 11.7 Hz, 1H), 1.59 (d, *J* = 10.5 Hz, 3H), 1.20–1.13 (m, 2H), 1.07–0.96 (m, 1H); ^{13}C NMR (100 MHz, $CDCl_3$) δ 165.7 (C), 157.5 (C), 157.0 (C), 156.4 (C), 136.8 (C), 135.6 (C), 135.3 (C), 133.1 (C), 132.1 (C), 130.2 (CH), 129.7 (CH), 129.6 (CH), 129.3 (CH), 129.2 (CH), 128.4 (CH), 127.99 (CH), 127.95 (CH), 80.7 (C), 34.2 (CH_2), 25.6 (CH_2), 23.4 (CH_2); IR (neat) 2935, 2849, 2028, 1990, 1968, 1607, 1540, 1489, 1444, 1380,

1108, 1089, 1015, 992, 899, 838, 757, 701, 620 cm^{-1}; MS (ESI, *m/z*): 450.17 $[M+H]^+$; HRMS (ESI) calcd. for $C_{29}H_{25}ClN_3$: 450.1732 $[M+H]^+$, found: 450.1736.

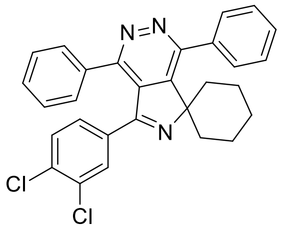

7'-(3,4-Dichlorophenyl)-1',4'-diphenylspiro[cyclohexane-1,5'-pyrrolo[3,4-*d*] pyridazine] (SPP-7). (2-(3,4-Dichlorophenyl)-1-azaspiro[4.5]deca-1,3-diene-3,4-diyl)bis (phenylmethanone) (**SP-7**) (45.0 mg, 0.09 mmol) and $NH_2NH_2.H_2O$ (46.1 mg, 0.92 mmol) were employed to afford 20.6 mg (46%) of the indicated product as a white solid (R_f = 0.15 in 4:1 hexane/ethyl acetate; mp 247.7–248.9 °C). ^{1}H NMR (400 MHz, $CDCl_3$) δ 7.64–7.58 (m, 3H), 7.57-7.53 (m, 2H), 7.39 (t, *J* = 7.9 Hz, 3H), 7.29 (dd, *J* = 12.3, 4.5 Hz, 3H), 7.18 (d, *J* = 8.3 Hz, 1H), 7.03 (dd, *J* = 8.2, 2.0 Hz, 1H), 2.08–1.99 (m, 4H), 1.86 (d, *J* = 12.6 Hz, 1H), 1.69 (d, *J* = 9.5 Hz, 2H), 1.28 (s, 2H), 1.13 (dt, *J* = 12.6, 6.2 Hz, 1H); ^{13}C NMR (100 MHz, $CDCl_3$) δ 164.7 (C), 157.6 (C), 157.0 (C), 156.3 (C), 136.7 (C), 135.6 (C), 134.4 (C), 133.6 (C), 132.3 (C), 131.9 (C), 130.6 (CH), 130.1 (CH), 129.7 (CH), 129.62 (CH), 129.58 (CH), 129.3 (CH), 128.4 (CH), 128.2 (CH), 127.7 (CH), 80.9 (C), 34.2 (CH_2), 25.6 (CH_2), 23.4 (CH_2); IR (neat) 2946, 1551, 1467, 1386, 1133, 1028, 1003, 942, 903, 833, 805, 758, 735, 696, 680, 662 cm^{-1}; MS (ESI, *m/z*): 484.13 $[M+H]^+$; HRMS (ESI) calcd. for $C_{29}H_{24}Cl_2N_3$: 484.1342 $[M+H]^+$, found: 484.1339.

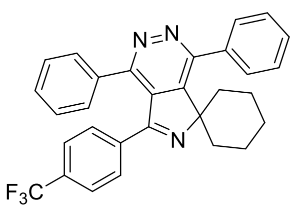

1',4'-Diphenyl-7'-(4-(trifluoromethyl)phenyl)spiro[cyclohexane-1,5'-pyrrolo[3,4-*d*] pyridazine] (SPP-8). (2-(4-(Trifluoromethyl)phenyl)-1-azaspiro[4.5]deca-1,3-diene-3,4-diyl)bis (phenylmethanone) (**SP-8**) (68.0 mg, 0.14 mmol) and $NH_2NH_2.H_2O$ (70.1 mg, 1.40 mmol) were employed to afford 56.4 mg (83%) of the indicated product as a light yellow solid (R_f = 0.08 in 4:1 hexane/ethyl acetate; mp 222.0–224.3 °C). ^{1}H NMR (400 MHz, $CDCl_3$) δ 7.65–7.55 (m, 5H), 7.33 (dt, *J* = 14.2, 8.1 Hz, 7H), 7.16 (dd, *J* = 10.6, 4.7 Hz, 2H), 2.12–1.98 (m, 4H), 1.86 (d, *J* = 12.5 Hz, 1H), 1.70 (d, *J* = 9.1 Hz, 2H), 1.30 (d, *J* = 9.3 Hz, 2H), 1.12 (d, *J* = 10.5 Hz, 1H); ^{13}C NMR (100 MHz, $CDCl_3$) δ 165.7 (C), 157.6 (C), 156.9 (C), 156.3 (C), 138.2 (C), 136.7 (C), 135.4 (C), 132.1 (C), 130.7 (q, ^2J = 35.6 Hz, C), 130.1 (CH), 129.6 (CH), 128.7 (CH), 128.4 (CH), 127.9 (CH), 124.7 (q, ^3J = 3.4 Hz, CH), 124.0 (q, ^1J = 207.0 Hz, CF_3) 122.6 (CH), 81.0 (C), 34.2 (CH_2), 25.6 (CH_2), 23.4 (CH_2) (Two CH peaks overlap on each other); IR (neat) 2940, 1594, 1540, 1440, 1409, 1375, 1326, 1162, 1124, 1108, 1065, 1020, 896, 843, 774, 758, 723, 701, 666, 605 cm^{-1}; MS (ESI, *m/z*): 484.20 $[M+H]^+$; HRMS (ESI) calcd. for $C_{30}H_{25}F_3N_3$: 484.1995 $[M+H]^+$, found: 484.1998.

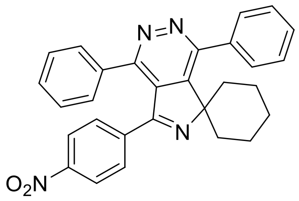

7'-(4-Nitrophenyl)-1',4'-diphenylspiro[cyclohexane-1,5'-pyrrolo[3,4-*d*]pyridazine] (SPP-9). (2-(4-Nitrophenyl)-1-azaspiro[4.5]deca-1,3-diene-3,4-diyl)bis(phenylmethanone) (**SP-9**) (28.0 mg, 0.06 mmol) and $NH_2NH_2.H_2O$ (30.0 mg, 0.60 mmol) were employed to afford 21.7 mg (79%) of the indicated product as a light orange solid (R_f = 0.10 in 4:1 hexane/ethyl acetate; mp 285.3–286.6 °C). ^{1}H NMR (400 MHz, $CDCl_3$) δ 7.88 (d, *J* = 8.8 Hz, 2H), 7.54–7.50 (m, 3H), 7.48–7.44 (m, 2H), 7.30–7.25 (m, 4H), 7.24–7.19 (m, 1H), 7.10 (t, *J* = 7.6 Hz, 2H), 2.01–1.87 (m, 5H), 1.77 (d, *J* = 12.6 Hz, 1H), 1.61 (d, *J* = 5.1 Hz, 2H), 1.20 (dd, *J* = 7.8, 4.3 Hz, 2H); ^{13}C NMR (100 MHz, $CDCl_3$) δ 165.1 (C), 157.7 (C), 157.0 (C), 156.1 (C), 148.1 (C), 140.9 (C), 136.6 (C), 135.5 (C), 131.8 (C), 130.1 (CH), 129.6 (CH), 129.43 (CH), 129.40 (CH), 128.5 (CH), 128.1 (CH), 122.9 (CH), 81.4 (C), 34.2 (CH_2), 25.6 (CH_2), 23.4 (CH_2) (Two CH peaks overlap on each other); IR (neat) 2937, 1582, 1519, 1444, 1381, 1350, 1105, 1015, 992, 899, 865, 847, 760, 721, 703 cm^{-1}; MS (ESI, *m/z*): 461.20 $[M+H]^+$; HRMS (ESI) calcd. for $C_{29}H_{25}N_4O_2$: 461.1972 $[M+H]^+$, found: 461.1965.

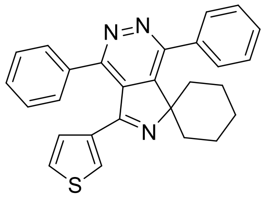

1',4'-Diphenyl-7'-(thiophen-3-yl)spiro[cyclohexane-1,5'-pyrrolo[3,4-*d*]pyridazine] (SPP-10). (2-(Thiophen-3-yl)-1-azaspiro[4.5]deca-1,3-diene-3,4-diyl)bis(phenylmethanone) (**SP-10**) (66.1 mg, 0.15 mmol) and $NH_2NH_2.H_2O$ (80.1 mg, 1.60 mmol) were employed to afford 58.1 mg (86%) of the indicated product as a white solid (R_f = 0.17 in 4:1 hexane/ethyl acetate; mp 185.5–188.0 °C). ^{1}H NMR (400 MHz, $CDCl_3$) δ 7.60-7.57 (m, 3H), 7.54 (dd, *J* = 6.7, 3.0 Hz, 2H), 7.48 (d, *J* = 7.1 Hz, 2H), 7.39 (t, *J* = 7.4 Hz, 1H), 7.29 (t, *J* = 7.5 Hz, 2H), 7.09–7.02 (m, 2H), 6.92 (d, *J* = 4.6 Hz, 1H), 2.08-1.97 (m, 4H), 1.84 (d, *J* = 12.8 Hz, 1H), 1.66 (d, *J* = 12.3 Hz, 2H), 1.27 (d, *J* = 10.7 Hz, 2H), 1.14–1.07 (m, 1H); ^{13}C NMR (100 MHz, $CDCl_3$) δ 161.9 (C), 157.3 (C), 156.8 (C), 156.4 (C), 136.9 (C), 136.0 (C), 135.7 (C), 132.4 (C), 130.1 (CH), 129.6 (CH), 129.2 (CH), 128.3 (CH), 128.1 (CH), 127.7 (CH), 126.9 (CH), 124.9 (CH), 80.2 (C), 34.3 (CH_2), 25.7 (CH_2), 23.4 (CH_2) (Two CH peaks overlap on each other); IR (neat) 2933, 1586, 1447, 1376, 1025, 945, 905, 856, 797, 761, 693, 661, 625 cm^{-1}; MS (ESI, *m/z*): 422.17 $[M+H]^+$; HRMS (ESI) calcd. for $C_{27}H_{24}N_3S$: 422.1685 $[M+H]^+$, found: 422.1685.

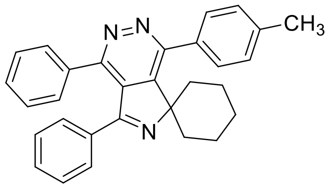

1',7'-Diphenyl-4'-(*p*-tolyl)spiro[cyclohexane-1,5'-pyrrolo[3,4-*d*]pyridazine] (SPP-11). (3-Benzoyl-2-phenyl-1-azaspiro[4.5]deca-1,3-dien-4-yl)(*p*-tolyl)methanone (**SP-11**) (40.0 mg, 0.09 mmol) and $NH_2NH_2.H_2O$ (45.1 mg, 0.90 mmol) were employed to afford 32.2 mg (83%) of the indicated product as a light yellow solid (R_f = 0.16 in 4:1 hexane/ethyl acetate; mp 249.7–251.3 °C). ^{1}H NMR (400 MHz, $CDCl_3$) δ 7.45 (d, *J* = 7.8 Hz, 2H), 7.41–7.35 (m, 4H), 7.26 (dd, *J* = 12.5, 5.4 Hz, 2H), 7.22–7.14 (m, 4H), 7.11 (dd, *J* = 13.9, 6.3 Hz, 2H), 2.52 (s, 3H), 2.15–2.00 (m, 4H), 1.85 (d, *J* = 12.9 Hz, 1H), 1.69 (d, *J* = 10.1 Hz, 2H), 1.29 (d, *J* = 6.6 Hz, 2H), 1.16 (dd, *J* = 18.2, 9.3 Hz, 1H); ^{13}C NMR (100 MHz, $CDCl_3$) δ 166.8 (C), 157.5 (C), 157.1 (C), 156.4 (C), 139.0 (C), 135.8 (C), 134.7 (C), 134.0 (C), 132.3 (C), 130.1 (CH), 129.5 (CH), 129.1 (CH), 129.0 (CH), 128.9 (CH), 128.3 (CH), 127.8 (CH), 80.5 (C), 34.2 (CH_2), 25.7(CH_2), 23.4 (CH_2), 21.6 (CH_3) (Two CH peaks overlap on each other); IR (neat) 2931, 1566, 1542, 1491, 1445, 1373, 1096, 1021, 992, 898, 830, 819, 764, 727, 710, 694, 664, 634 cm^{-1}; MS (ESI, m/z): 430.23 $[M+H]^+$; HRMS (ESI) calcd. for $C_{30}H_{28}N_3$: 430.2278 $[M+H]^+$, found: 430.2278.

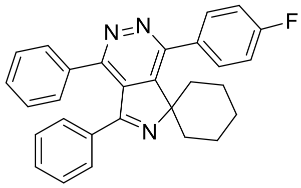

4'-(4-Fluorophenyl)-1',7'-diphenylspiro[cyclohexane-1,5'-pyrrolo[3,4-*d*]pyridazine] (SPP-12). (3-Benzoyl-2-phenyl-1-azaspiro[4.5]deca-1,3-dien-4-yl)(4-fluorophenyl)methanone (**SP-12**) (84.1 mg, 0.19 mmol) and $NH_2NH_2.H_2O$ (95.1 mg, 1.92 mmol) were employed to afford 69.0 mg (84%) of the indicated product as a light orange solid (R_f = 0.17 in 4:1 hexane/ethyl acetate; mp 195.5–195.9 °C). ^{1}H NMR (400 MHz, $CDCl_3$) δ 7.47 (dd, *J* = 8.4, 5.4 Hz, 2H), 7.28 (d, *J* = 7.2 Hz, 2H), 7.24–7.15 (m, 4H), 7.12–6.99 (m, 6H), 1.99 (dd, *J* = 17.2, 8.8 Hz, 2H), 1.89 (td, *J* = 12.9, 3.2 Hz, 2H), 1.79 (d, *J* = 12.9 Hz, 1H), 1.61 (d, *J* = 12.8 Hz, 2H), 1.21 (d, *J* = 12.8 Hz, 2H), 1.06 (d, *J* = 13.4 Hz, 1H); ^{13}C NMR (100 MHz, $CDCl_3$) δ 166.7 (C), 163.3 (d, ^1J = 248.8 Hz, C), 157.2 (C), 156.7 (C), 156.5 (C), 135.5 (C), 134.4 (C), 132.9 (d, ^4J = 3.34 Hz, C), 132.4 (C), 131.6 (CH),131.5 (CH), 130.1 (CH), 129.1 (d, ^3J = 15.1 Hz, CH), 128.2 (CH), 127.80 (CH), 127.76 (CH), 115.5 (d, ^2J = 21.7, CH), 80.4 (C), 34.2 (CH_2), 25.6 (CH_2), 23.3 (CH_2); IR (neat) 2927, 1737,1604, 1567, 1505, 1445, 1378, 1219, 1156, 1095, 1043, 1014, 993, 900, 846, 824, 812, 763, 730, 708, 696, 663, 632 cm^{-1}; MS (ESI, *m/z*): 434.20 $[M+H]^+$; HRMS (ESI) calcd. for $C_{29}H_{25}FN_3$: 434.2027 $[M+H]^+$, found: 434.2028.

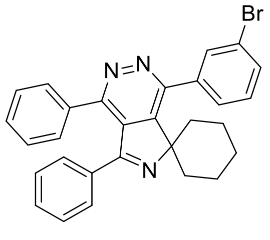

4'-(3-Bromophenyl)-1',7'-diphenylspiro[cyclohexane-1,5'-pyrrolo[3,4-*d*]pyridazine] (SPP-13). (3-Benzoyl-2-phenyl-1-azaspiro[4.5]deca-1,3-dien-4-yl)(3-bromophenyl)methanone (**SP-13**) (82.3 mg, 0.17 mmol) and $NH_2NH_2.H_2O$ (85.1 mg, 1.70 mmol) were employed to afford 68.5 mg (82%) of the indicated product as a yellow solid (R_f = 0.16 in 4:1 hexane/ethyl acetate; mp 184.4–186.7 °C). ^{1}H NMR (400 MHz, $CDCl_3$) δ 7.74 (dd, *J* = 6.7, 1.5 Hz, 2H), 7.53–7.45 (m, 2H), 7.39–7.35 (m, 2H), 7.26 (dd, *J* = 15.0, 7.5 Hz, 2H), 7.21–7.07 (m, 6H), 2.15–2.05 (m, 2H), 1.98 (td, *J* = 12.9, 3.4 Hz, 2H), 1.88 (d, *J* = 12.9 Hz, 1H), 1.72 (d, *J* = 12.9 Hz, 2H), 1.32 (d, *J* = 12.4 Hz, 2H), 1.19–1.12 (m, 1H); ^{13}C NMR (100 MHz, $CDCl_3$) δ 166.7 (C), 157.0 (C), 156.9 (C), 155.8 (C), 138.8 (C), 135.4 (C), 134.4 (C), 132.7 (C), 132.4 (C), 132.3 (CH), 130.1 (CH), 129.9 (CH), 129.2 (CH), 129.1 (CH), 128.30 (CH), 128.26 (CH), 127.83 (CH), 127.78 (CH), 122.5 (CH), 80.4 (C), 34.4 (CH_2), 25.7 (CH_2), 23.3 (CH_2); IR (neat) 2928, 1737, 1562, 1444, 1371, 1237, 1042, 996, 905, 848, 793, 764, 745, 713, 696, 679, 651 cm^{-1}; MS (ESI, *m/z*): 494.12 $[M+H]^+$; HRMS (ESI) calcd. for $C_{29}H_{25}BrN_3$: 494.1226 $[M+H]^+$, found: 494.1227.

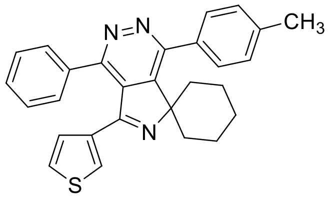

1'-Phenyl-7'-(thiophen-3-yl)-4'-(*p*-tolyl)spiro[cyclohexane-1,5'-pyrrolo[3,4-*d*] pyridazine] (SPP-14). (3-Benzoyl-2-(thiophen-3-yl)-1-azaspiro[4.5]deca-1,3-dien-4-yl)(*p*-tolyl) methanone (**SP-14**) (34.0 mg, 0.08 mmol) and $NH_2NH_2.H_2O$ (40.0 mg, 0.80 mmol) were employed to afford 29.0 mg (87%) of the indicated product as a light orange solid (R_f = 0.20 in 4:1 hexane/ethyl acetate; mp 202.7–203.6 °C). ^{1}H NMR (400 MHz, $CDCl_3$) δ 7.49-7.42 (m, 5H), 7.40-7.37 (m, 2H), 7.28 (t, *J* = 7.5 Hz, 2H), 7.09-7.03 (m, 2H), 6.92 (dd, *J* = 4.9, 1.0 Hz, 1H), 2.52 (s, 3H), 2.11–1.99 (m, 5H), 1.85 (d, *J* = 12.7 Hz, 1H), 1.68 (d, *J* = 10.3 Hz, 2H), 1.26 (d, *J* = 10.8, 2H); ^{13}C NMR (100 MHz, $CDCl_3$) δ 162.0 (C), 157.4 (C), 156.9 (C), 156.3 (C), 139.0 (C), 136.0 (C), 135.7 (C), 134.0 (C), 132.4 (C), 130.1 (CH), 129.5 (CH), 129.2 (CH), 129.0 (CH), 128.1 (CH), 127.7 (CH), 126.9 (CH), 124.9 (CH), 80.2 (C), 34.2 (CH_2), 25.7(CH_2), 23.4 (CH_2), 21.6 (CH_3); IR (neat) 2919, 2847, 1572, 1503, 1443, 1379, 1276, 1072, 1021, 945, 904, 865, 825, 799, 766, 722, 699, 637, 622 cm^{-1}; MS (ESI, *m/z*): 436.18 $[M+H]^+$; HRMS (ESI) calcd. for $C_{28}H_{26}N_3S$: 436.1842 $[M+H]^+$, found: 436.1845.

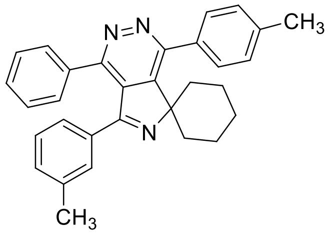

1'-Phenyl-7'-(*m*-tolyl)-4'-(*p*-tolyl)spiro[cyclohexane-1,5'-pyrrolo[3,4-*d*]pyridazine] (SPP-15). (3-Benzoyl-2-(*m*-tolyl)-1-azaspiro[4.5]deca-1,3-dien-4-yl)(*p*-tolyl)methanone (**SP-15**) (97.2 mg, 0.22 mmol) and $NH_2NH_2.H_2O$ (110.1 mg, 2.20 mmol) were employed to afford 86.0 mg (88%) of the indicated product as a yellow solid (R_f = 0.22 in 4:1 hexane/ethyl acetate; mp 231.0–233.6 °C). ^{1}H NMR (400 MHz, $CDCl_3$) δ 7.45 (s, 1H), 7.39 (d, *J* = 8.2 Hz, 4H), 7.28 (d, *J* = 7.4 Hz, 1H), 7.17 (t, *J* = 7.5 Hz, 3H), 7.10 (d, *J* = 3.3 Hz, 1H), 7.05 (s, 2H), 6.91 (s, 1H), 2.53 (s, 3H), 2.11 (d, *J* = 2.7 Hz, 3H), 2.07 (s, 4H), 1.85 (d, *J* = 12.7 Hz, 1H), 1.69 (d, *J* = 9.3 Hz, 2H), 1.28 (dd, *J* = 7.1, 3.8 Hz, 3H); ^{13}C NMR (100 MHz, $CDCl_3$) δ 166.8 (C), 157.5 (C), 157.0 (C), 156.5 (C), 138.9 (C), 137.4 (C), 135.9 (C), 134.4 (C), 134.0 (C), 132.3 (C), 130.0 (CH), 129.8 (CH), 129.5 (CH), 129.2 (CH), 129.0 (CH), 128.8 (CH), 127.8 (CH), 127.7 (CH), 125.4 (CH), 80.4 (C), 34.2 (CH_2), 25.7 (CH_2), 23.4 (CH_2), 21.5 (CH_3), 21.1 (CH_3); IR (neat) 2950, 2912,1571, 1508, 1433, 1372, 1189, 1097, 1040, 1000, 940, 904, 851, 818, 790, 768, 729, 718, 695, 664, 637, 602 cm^{-1}; MS (ESI, *m/z*): 444.24 $[M+H]^+$; HRMS (ESI) calcd. for $C_{31}H_{30}N_3$: 444.2434 $[M+H]^+$, found: 444.2440.

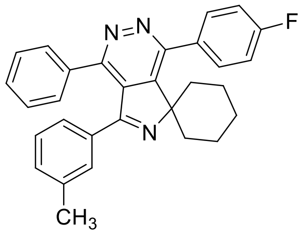

4'-(4-Fluorophenyl)-1'-phenyl-7'-(*m*-tolyl)spiro[cyclohexane-1,5'-pyrrolo[3,4-*d*] pyridazine] (SPP-16). (3-Benzoyl-2-(*m*-tolyl)-1-azaspiro[4.5]deca-1,3-dien-4-yl)(4-fluorophenyl)methanone (**SP-16**) (78.0 mg, 0.17 mmol) and $NH_2NH_2.H_2O$ (85.1 mg, 1.70 mmol) were employed to afford 60.0 mg (78%) of the indicated product as a light yellow solid (R_f = 0.16 in 4:1 hexane/ethyl acetate; mp 186.5–186.8 °C). ^{1}H NMR (400 MHz, $CDCl_3$) δ 7.55 (t, *J* = 6.9 Hz, 2H), 7.41–7.36 (m, 2H), 7.30 (dd, *J* = 11.8, 5.1 Hz, 3H), 7.17 (t, *J* = 7.5 Hz, 2H), 7.08 (dd, *J* = 16.3, 2.1 Hz, 3H), 6.90 (s, 1H), 2.11 (s, 3H), 2.06 (s, 1H), 1.97 (t, *J* = 12.9 Hz, 2H), 1.87 (d, *J* = 12.5 Hz, 1H), 1.69 (d, *J* = 12.2 Hz, 3H), 1.29 (d, *J* = 12.1 Hz, 2H), 1.21–1.10 (m, 1H); ^{13}C NMR (100 MHz, $CDCl_3$) δ 166.8 (C), 163.4 (d, ^1J = 248.7 Hz, C), 157.2 (C), 156.8 (C), 156.5 (C), 137.5 (C), 135.7 (C), 134.3 (C), 133.2 (d, ^4J = 3.3 Hz, C), 132.5 (C), 131.6 (d, ^3J = 8.3 Hz, CH), 130.1 (CH), 129.9 (CH), 129.2 (CH), 129.0 (CH), 127.8 (CH), 127.7 (CH), 125.4 (CH), 115.5 (d, ^2J = 21.7 Hz, CH), 80.3 (C), 34.3 (CH_2), 25.7 (CH_2), 23.3 (CH_2), 21.1 (CH_3); IR (neat) 2919, 2853, 1605, 1574, 1503, 1445, 1376, 1221, 1157, 1097, 1023, 940, 904, 851, 842, 822, 804, 764, 733, 717, 693, 663, 601 cm^{-1}; MS (ESI, *m/z*): 448.22 $[M+H]^+$; HRMS (ESI) calcd. for $C_{30}H_{27}FN_3$: 448.2184 $[M+H]^+$, found: 448.2184.

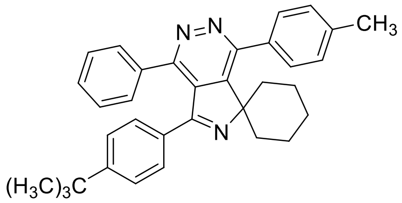

7'-(4-(*tert*-Butyl)phenyl)-1'-phenyl-4'-(*p*-tolyl)spiro[cyclohexane-1,5'-pyrrolo[3,4-*d*] pyridazine] (SPP-17). (3-Benzoyl-2-(4-(*tert*-butyl)phenyl)-1-azaspiro[4.5]deca-1,3-dien-4-yl)(*p*-tolyl)methanone (**SP-17**) (84.0 mg, 0.17 mmol) and $NH_2NH_2.H_2O$ (85.1 mg, 1.70 mmol) were employed to afford 71.3 mg (86%) of the indicated product as a light yellow solid (R_f = 0.22 in 4:1 hexane/ethyl acetate; mp 250.4–252.7 °C). ^{1}H NMR (400 MHz, $CDCl_3$) δ 7.45 (d, *J* = 7.9 Hz, 2H), 7.39 (d, *J* = 7.8 Hz, 2H), 7.33 (d, *J* = 7.1 Hz, 2H), 7.24 (t, *J* = 7.2 Hz, 1H), 7.14–7.06 (m, 6H), 2.52 (s, 3H), 2.14–1.98 (m, 4H), 1.85 (d, *J* = 13.0 Hz, 1H), 1.68 (d, *J* = 11.6 Hz, 2H), 1.34 (d, *J* = 3.2 Hz, 1H), 1.29 (d, *J* = 2.2 Hz, 9H), 1.14 (d, *J* = 12.4 Hz, 1H); ^{13}C NMR (100 MHz, $CDCl_3$) δ 166.7 (C), 157.5 (C), 156.9 (C), 156.5 (C), 152.1 (C), 138.9 (C), 135.7 (C), 134.0 (C), 132.5 (C), 131.7 (C), 130.1 (CH), 129.5 (CH), 129.0 (CH), 128.6 (CH), 127.8 (CH), 127.6 (CH), 124.7 (CH), 80.4 (C), 34.6 (C), 34.1 (CH_2), 31.3 (CH_3), 25.7 (CH_2), 23.4 (CH_2), 21.6 (CH_3); IR (neat) 2922, 2853, 1739, 1611, 1584, 1505, 1445, 1406, 1379, 1267, 1112, 1041, 1017, 992, 934, 900, 840, 823, 769, 728, 714, 703, 662, 635, 601 cm^{-1}; MS (ESI, *m/z*): 486.29 $[M+H]^+$; HRMS (ESI) calcd. for $C_{34}H_{36}N_3$: 486.2904 $[M+H]^+$, found: 486.2905.

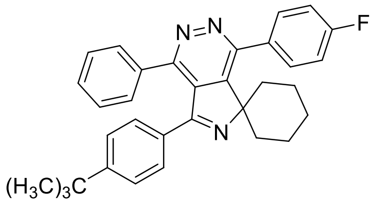

7'-(4-(*tert*-Butyl)phenyl)-4'-(4-fluorophenyl)-1'-phenylspiro[cyclohexane-1,5'-pyrrolo[3,4-*d*]pyridazine] (SPP-18). (3-Benzoyl-2-(4-(*tert*-butyl)phenyl)-1-azaspiro[4.5]deca-1,3-dien-4-yl)(4-fluorophenyl)methanone (**SP-18**) (42.0 mg, 0.09 mmol) and $NH_2NH_2.H_2O$ (42.6 mg, 0.85 mmol) were employed to afford 25.3 mg (61%) of the indicated product as a yellow solid (R_f = 0.16 in 4:1 hexane/ethyl acetate; mp 202.1–205.2 °C). ^{1}H NMR (400 MHz, $CDCl_3$) δ 7.55 (dd, *J* = 8.6, 5.3 Hz, 2H), 7.34–7.28 (m, 4H), 7.24 (d, *J* = 7.4 Hz, 1H), 7.15–7.06 (m, 6H), 2.08 (dd, *J* = 16.2, 9.8 Hz, 2H), 1.97 (td, *J* = 12.9, 3.4 Hz, 2H), 1.87 (d, *J* = 12.5 Hz, 1H), 1.69 (d, *J* = 12.7 Hz, 2H), 1.29 (s, 2H), 1.28 (s, 9H), 1.18–1.09 (m, 1H); ^{13}C NMR (100 MHz, $CDCl_3$) δ 166.8 (C), 163.4 (d, ^1J = 248.6 Hz, C), 157.1 (C), 156.9 (C), 156.5 (C), 152.4 (C), 135.6 (C), 133.1 (d, ^4J = 3.4 Hz, C), 132.7 (C), 131.6 (d, ^3J = 8.2 Hz, CH), 130.1 (CH), 128.8 (CH), 127.9 (CH), 127.7 (CH), 124.8 (CH), 115.5 (d, ^2J = 21.8 Hz, CH), 80.4 (C), 34.7 (C), 34.3 (CH_2), 31.3 (CH_3), 25.7 (CH_2), 23.4 (CH_2) (One C peak overlaps with one CH peak); IR (neat) 2937, 1589, 1504, 1445, 1379, 1268, 1221, 1156, 1114, 1016, 994, 900, 844, 765, 727, 697, 676, 660 cm^{-1}; MS (ESI, *m/z*): 490.27 $[M+H]^+$; HRMS (ESI) calcd. for $C_{33}H_{33}FN_3$: 490.2653 $[M+H]^+$, found: 490.2654.

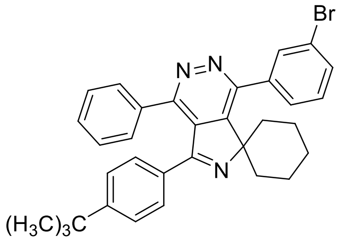

4'-(3-Bromophenyl)-7'-(4-(*tert*-butyl)phenyl)-1'-phenylspiro[cyclohexane-1,5'-pyrrolo[3,4-*d*]pyridazine] (SPP-19). (3-Benzoyl-2-(4-(*tert*-butyl)phenyl)-1-azaspiro[4.5]deca-1,3-dien-4-yl)(3-bromophenyl)methanone (**SP-19**) (198.2 mg, 0.36 mmol) and $NH_2NH_2.H_2O$ (180.2 mg, 3.60 mmol) were employed to afford 145.8 mg (74%) of the indicated product as a bright yellow solid (R_f = 0.37 in 4:1 hexane/ethyl acetate; mp 227.8–228.9 °C). ^{1}H NMR (400 MHz, $CDCl_3$) δ 7.64–7.59 (m, 2H), 7.41–7.32 (m, 2H), 7.23–7.20 (m, 2H), 7.13 (t, *J* = 7.5 Hz, 1H), 7.03–6.95 (m, 6H), 2.03–1.93 (m, 2H), 1.86 (td, *J* = 12.9, 3.4 Hz, 2H), 1.76 (d, *J* = 13.1 Hz, 1H), 1.59 (d, *J* = 12.9j Hz, 2H), 1.21 (s, 3H), 1.17 (s, 9H); ^{13}C NMR (100 MHz, $CDCl_3$) δ 166.6 (C), 156.9 (C), 156.8 (C), 155.8 (C), 152.3 (C), 138.9 (C), 135.4 (C), 132.7 (C), 132.2 (C), 131.5 (C), 130.1 (CH), 129.8 (CH), 128.7 (CH), 128.3 (CH), 127.8 (CH), 127.7 (CH), 124.7 (CH), 122.4 (CH), 80.3 (C), 34.6 (C), 34.4 (CH_2), 31.2 (CH_3), 25.7 (CH_2), 23.3 (CH_2) (Two CH peaks overlap on each other); IR (neat) 2943, 1610, 1584, 1560, 1461, 1445, 1409, 1371, 1267, 1113, 1023, 997, 907, 889, 837, 795, 767, 745, 718, 695, 650 cm^{-1}; MS (ESI, *m/z*): 550.19 [M+H]$^+$; HRMS (ESI) calcd. for $C_{33}H_{33}BrN_3$: 550.1852 [M+H]$^+$, found: 550.1851.

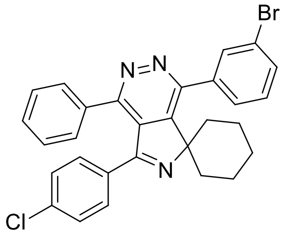

4'-(3-Bromophenyl)-7'-(4-chlorophenyl)-1'-phenylspiro[cyclohexane-1,5'-pyrrolo [3,4-*d*]pyridazine] (SPP-20). (3-Benzoyl-2-(4-chlorophenyl)-1-azaspiro[4.5]deca-1,3-dien-4-yl)(3-bromophenyl)methanone (**SP-20**) (70.5 mg, 0.13 mmol) and $NH_2NH_2.H_2O$ (65.1 mg, 1.30 mmol) were employed to afford 42.0 mg (61%) of the indicated product as a beige solid (R_f = 0.17 in 4:1 hexane/ethyl acetate; mp 219.2–221.0 °C). ^{1}H NMR (400 MHz, $CDCl_3$) δ 7.73 (dd, *J* = 5.4, 1.8 Hz, 2H), 7.48 (d, *J* = 8.0 Hz, 2H), 7.40–7.33 (m, 3H), 7.22 (t, *J* = 7.7 Hz, 2H), 7.10 (dd, *J* = 21.0, 8.6 Hz, 4H), 2.11–1.93 (m, 4H), 1.88 (d, *J* = 12.7 Hz, 1H), 1.72 (d, *J* = 12.4 Hz, 2H), 1.30 (d, *J* = 12.2 Hz, 2H), 1.19 (dd, *J* = 17.7, 8.2 Hz, 1H); ^{13}C NMR (100 MHz, $CDCl_3$) δ 165.6 (C), 157.0 (C), 156.7 (C), 155.9 (C), 138.7 (C), 135.4 (C), 132.9 (C), 132.7 (C), 132.4 (C), 132.2 (C), 130.2 (CH), 129.9 (CH), 129.7 (CH), 129.3 (CH), 128.3 (CH), 128.02 (CH), 127.96 (CH), 122.5 (CH), 80.6 (C), 34.3 (CH_2), 25.7 (CH_2), 23.4 (CH_2) (Two CH peaks overlap on each other); IR (neat) 2933, 1609, 1490, 1445, 1398, 1374, 1090, 1017, 998, 907, 884, 839, 789, 768, 730, 701 cm^{-1}; MS (ESI, *m/z*): 528.08 [M+H]$^+$; HRMS (ESI) calcd. for $C_{29}H_{24}BrClN_3$: 528.0837 [M+H]$^+$, found: 528.0838.

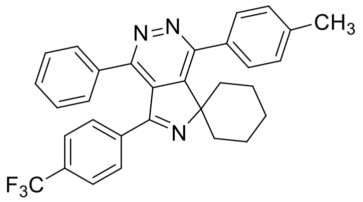

1'-Phenyl-4'-(*p*-tolyl)-7'-(4-(trifluoromethyl)phenyl)spiro[cyclohexane-1,5'-pyrrolo [3,4-*d*]pyridazine] (SPP-21). (3-Benzoyl-2-(4-(trifluoromethyl)phenyl)-1-azaspiro[4.5]deca-1,3-dien-4-yl)(*p*-tolyl)methanone (**SP-21**) (40.0 mg, 0.08 mmol) and $NH_2NH_2.H_2O$ (40.0 mg, 0.80 mmol) were employed to afford 38.0 mg (95%) of the indicated product as a light yellow solid (R_f = 0.14 in 4:1 hexane/ethyl acetate; mp 244.5–246.7 °C). ^{1}H NMR (400 MHz, $CDCl_3$) δ 7.43 (dd, *J* = 19.2, 8.0 Hz, 4H), 7.39–7.27 (m, 7H), 7.16 (t, *J* = 7.6 Hz, 2H), 2.53 (s, 3H), 2.07 (s, 4H), 1.87 (d, *J* = 12.2 Hz, 1H), 1.71 (s, 2H), 1.29 (d, *J* = 7.1 Hz, 2H), 1.17 (s, 1H); ^{13}C NMR (100 MHz, $CDCl_3$) δ 165.8 (C), 157.7 (C), 157.0 (C), 156.2 (C), 139.2 (C), 138.2 (C), 135.5 (C), 133.8 (C), 132.0 (C), 131.1 (q, ^2J = 32.5 Hz, C), 130.1 (CH), 129.5 (CH), 129.2 (CH), 129.1 (CH), 128.7 (CH), 128.0 (CH), 124.7 (q, ^3J = 3.64 Hz, CH), 124.0 (q, ^1J = 278.5 Hz, CF_3), 81.1 (C), 34.2 (CH_2), 25.6 (CH_2), 23.4 (CH_2), 21.6 (CH_3); IR (neat) 2934, 1595, 1445, 1407, 1382, 1326, 1161, 1122, 1064, 1021, 993, 901, 858, 820, 766, 727, 701, 661 cm^{-1}; MS (ESI, *m/z*): 498.22 $[M+H]^+$; HRMS (ESI) calcd. for $C_{31}H_{27}F_3N_3$: 498.2152 $[M+H]^+$, found: 498.2152.

Copies of ¹H and ¹³C NMR Spectra

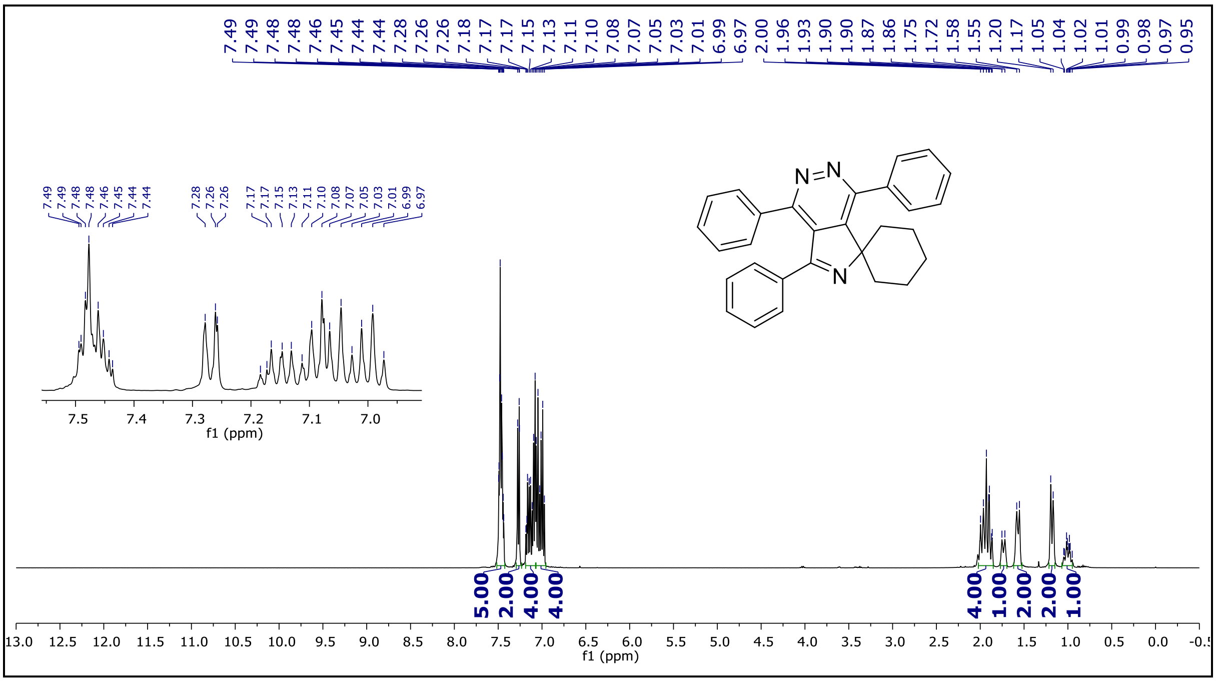

Figure S1. ¹H NMR spectrum of compound **SPP-1**.

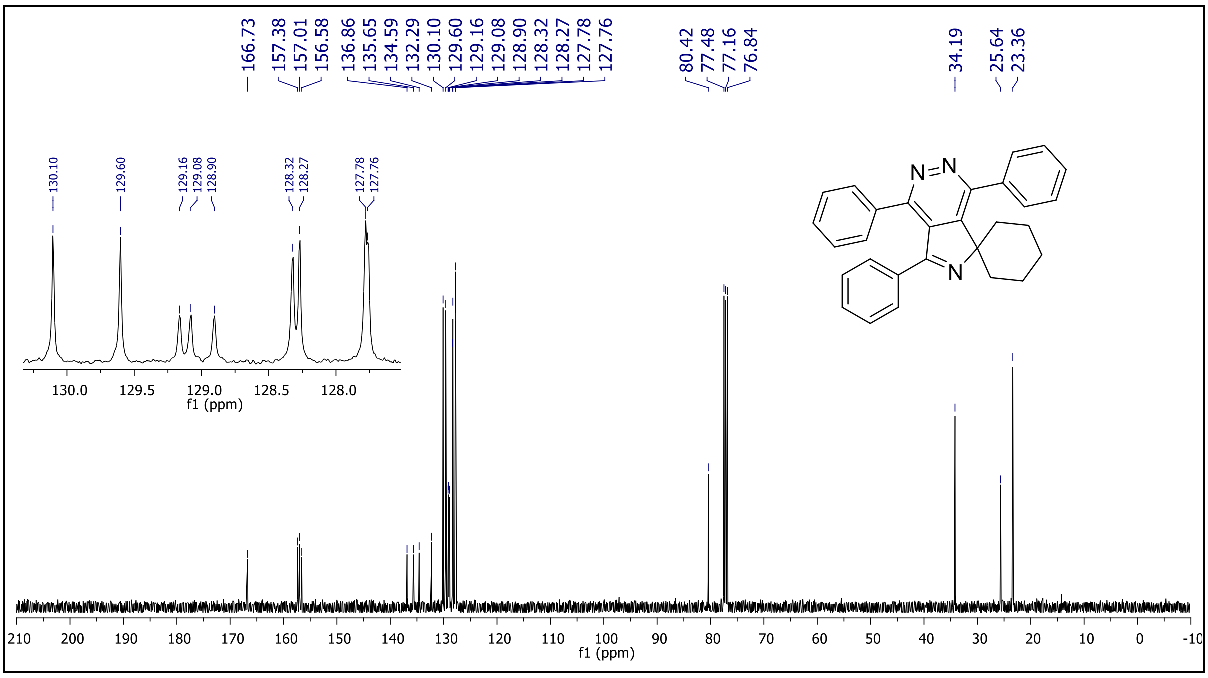

Figure S2. ¹³C NMR spectrum of compound **SPP-1**.

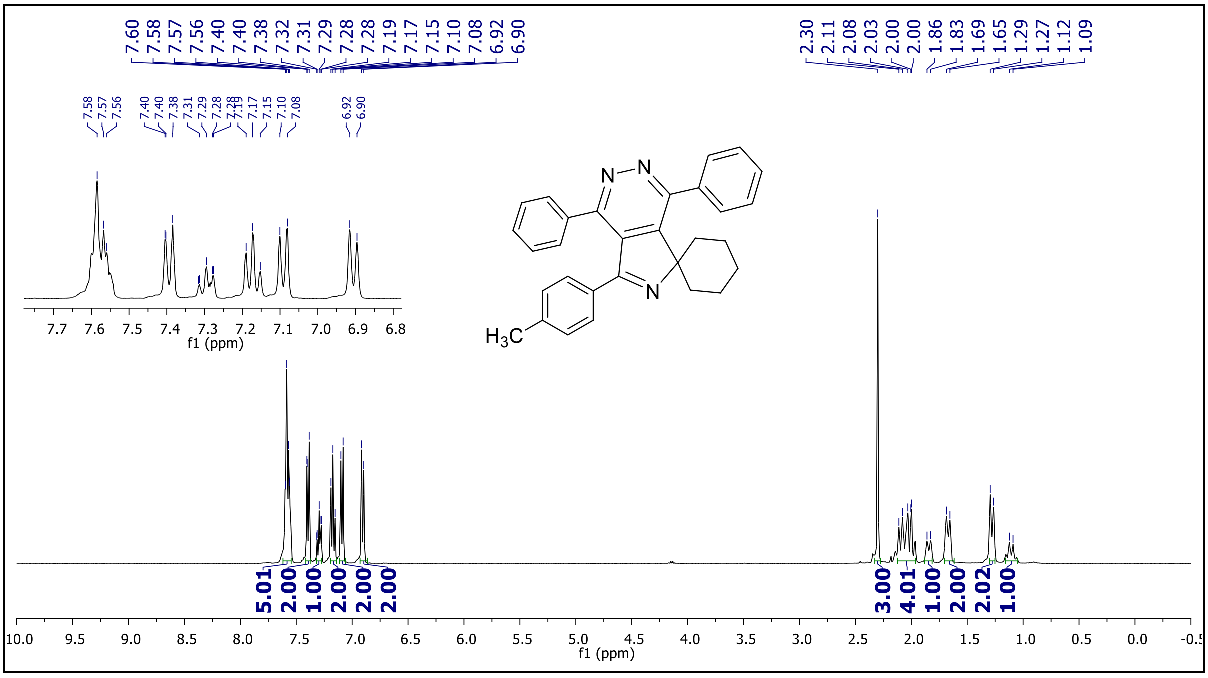

Figure S3. ^{1}H NMR spectrum of compound **SPP-2**.

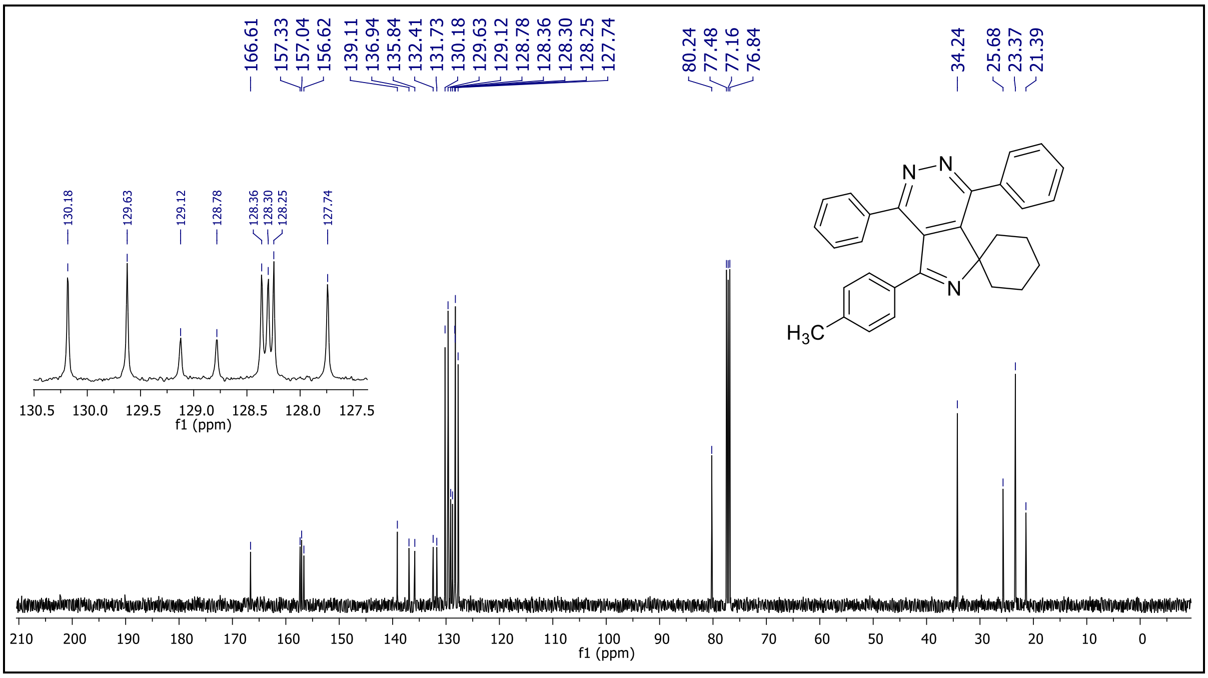

Figure S4. ^{13}C NMR spectrum of compound **SPP-2**.

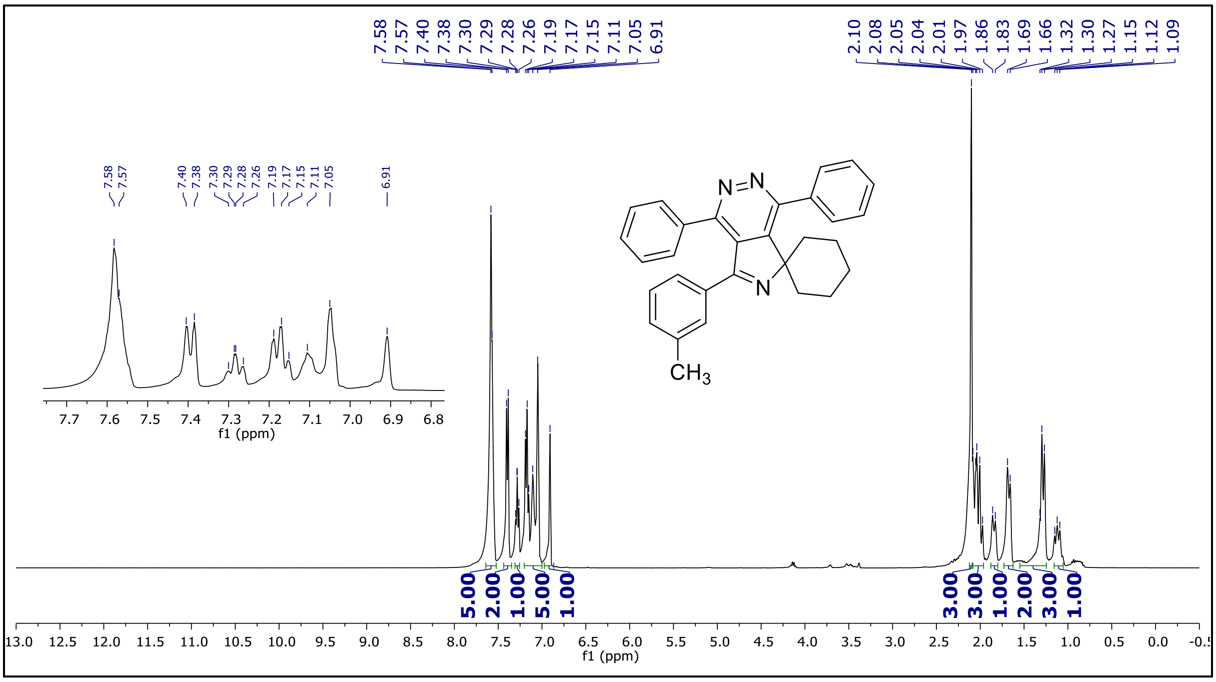

Figure S5. ^{1}H NMR spectrum of compound **SPP-3**.

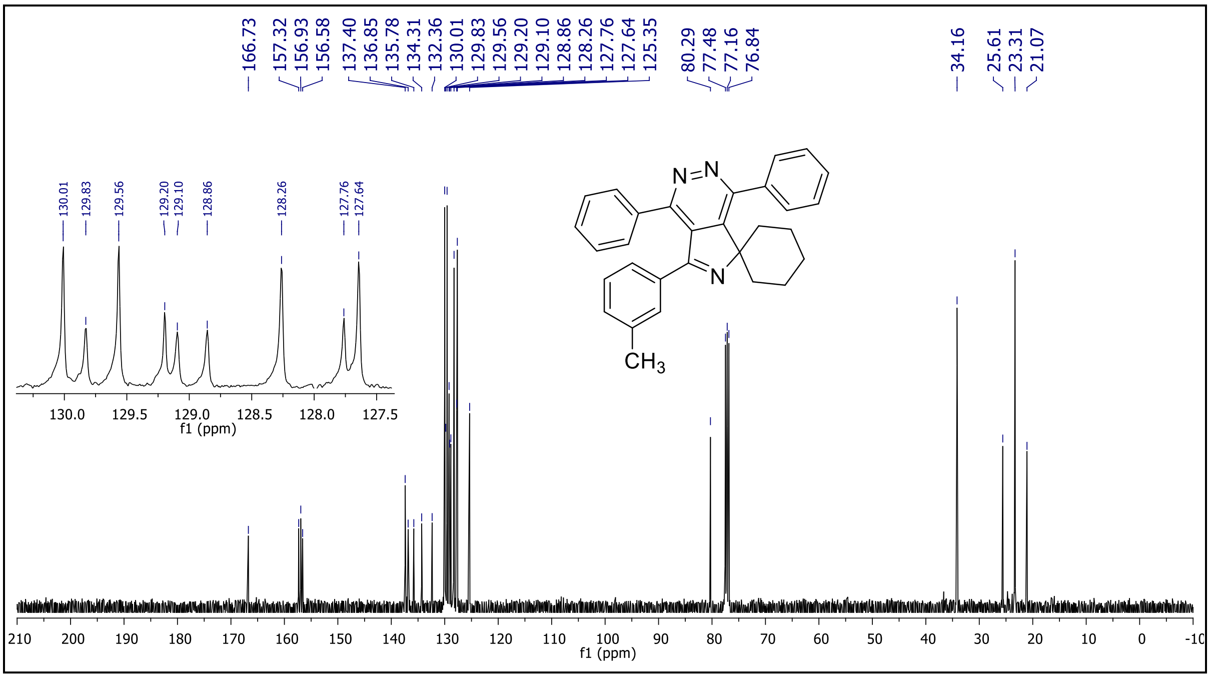

Figure S6. ^{13}C NMR spectrum of compound **SPP-3**.

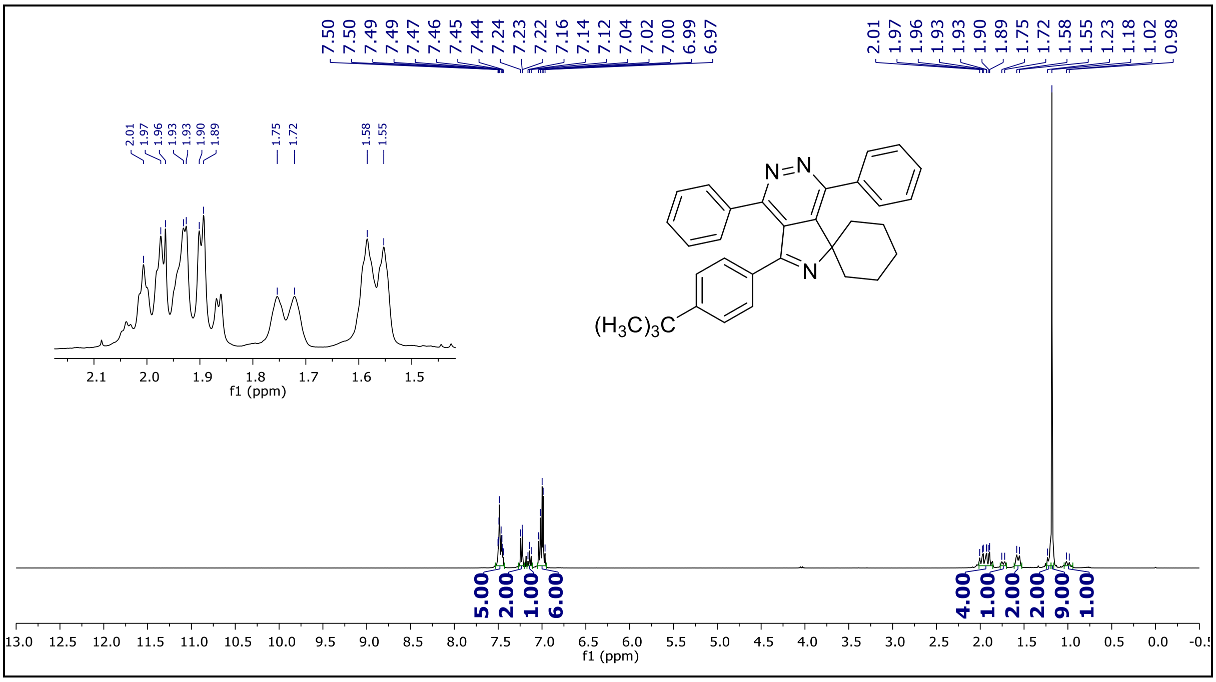

Figure S7. ^{1}H NMR spectrum of compound **SPP-4**.

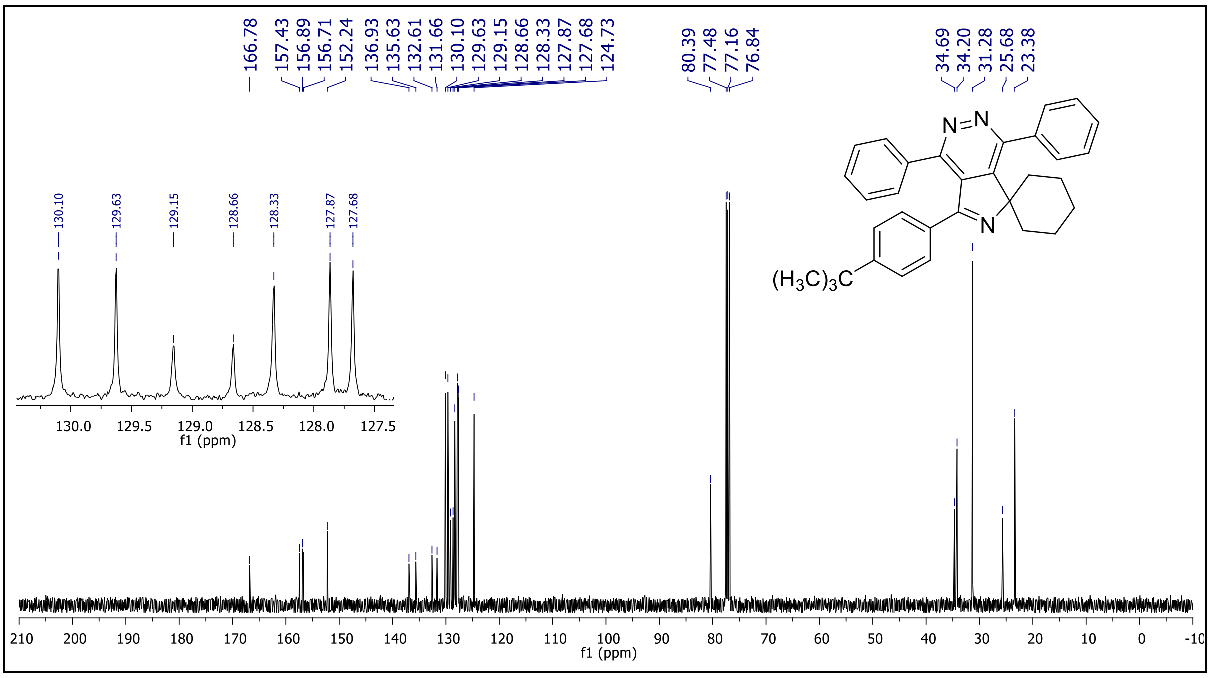

Figure S8. ^{13}C NMR spectrum of compound **SPP-4**.

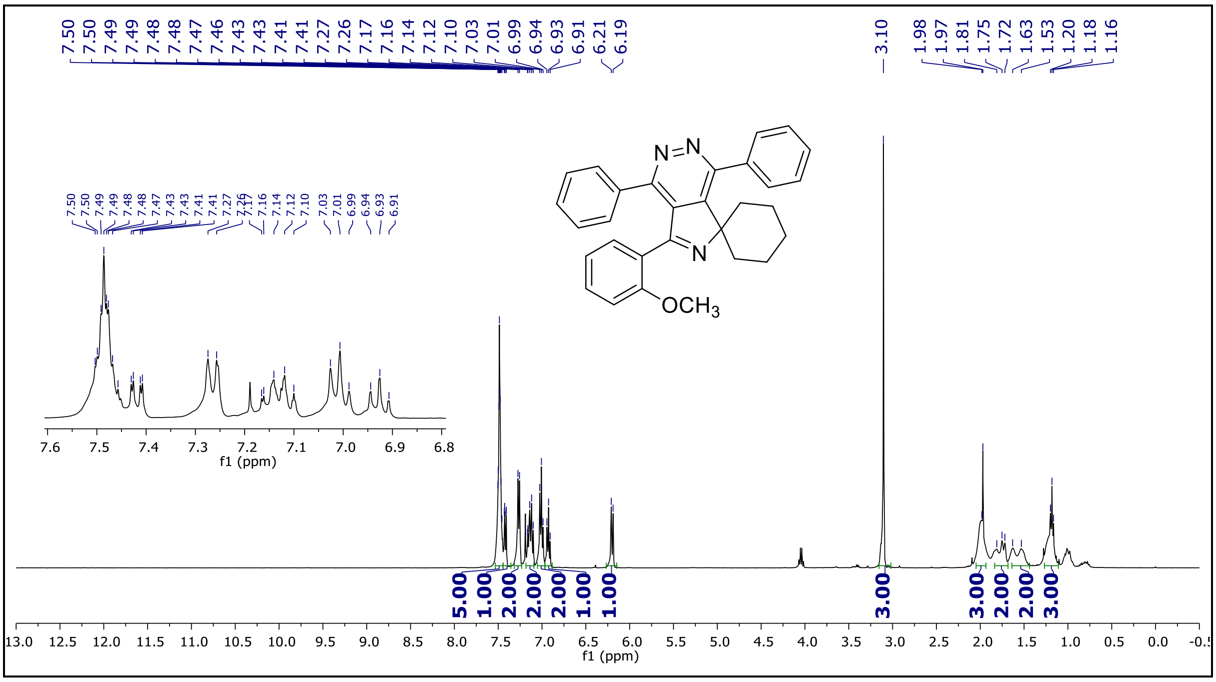

Figure S9. ^{1}H NMR spectrum of compound **SPP-5**.

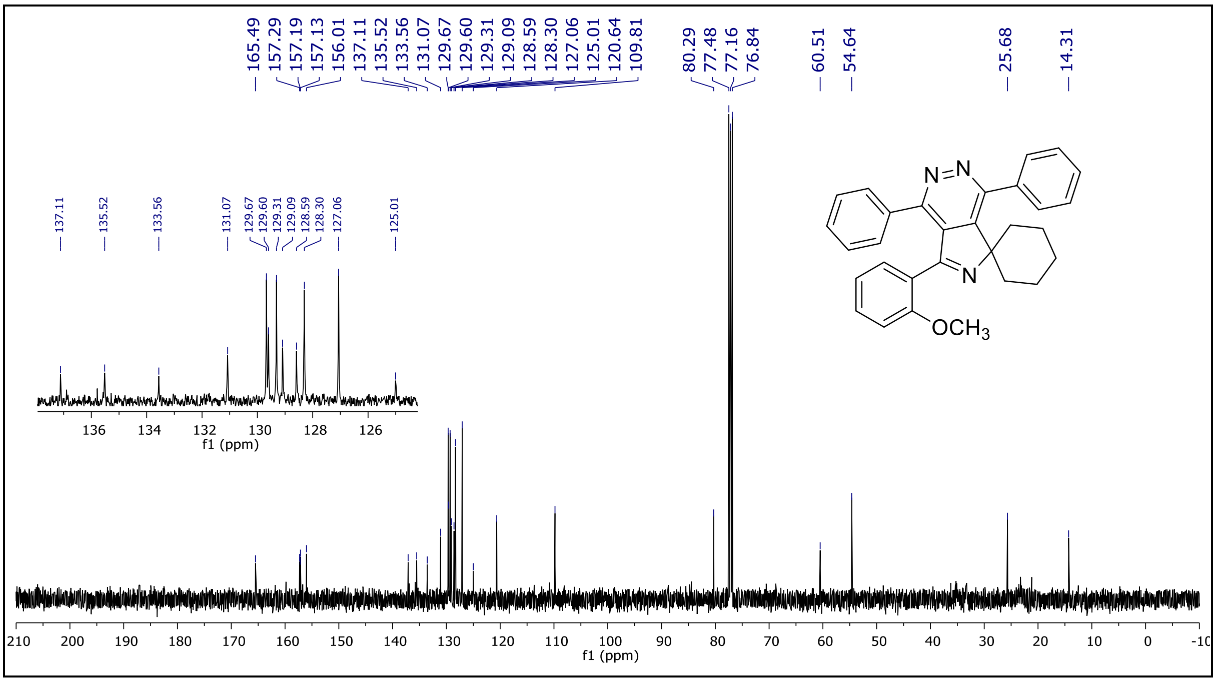

Figure S10. ^{13}C NMR spectrum of compound **SPP-5**.

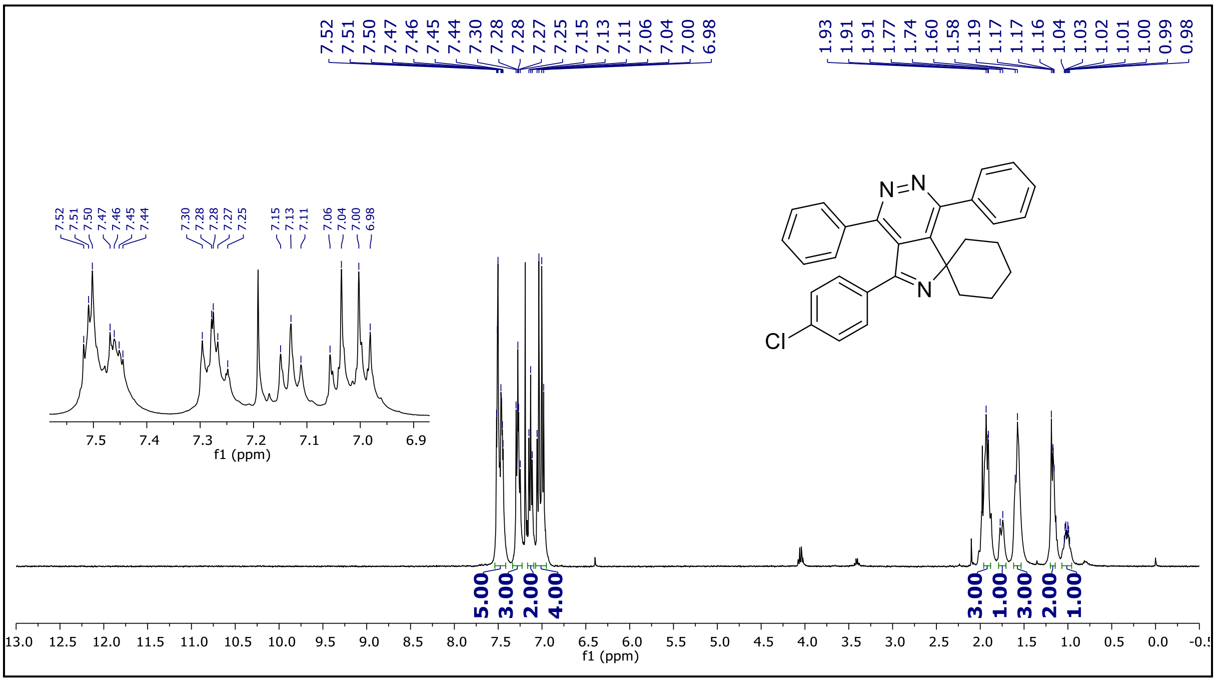

Figure S11. ^{1}H NMR spectrum of compound **SPP-6**.

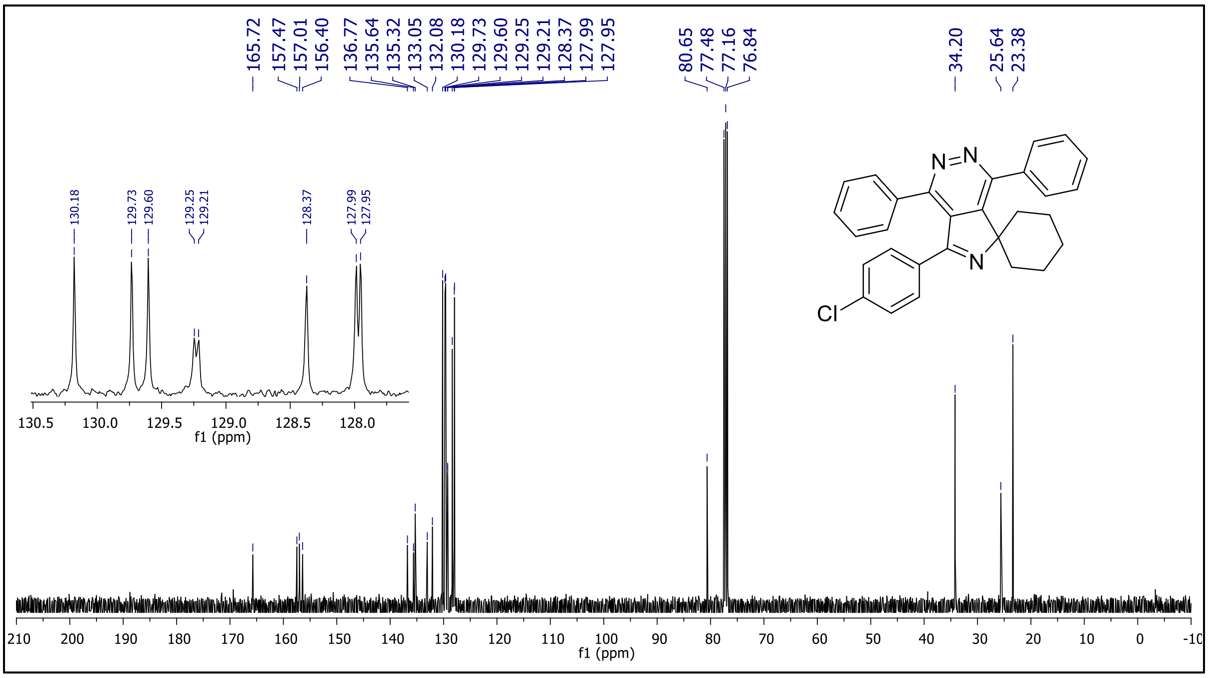

Figure S12. ^{13}C NMR spectrum of compound **SPP-6**.

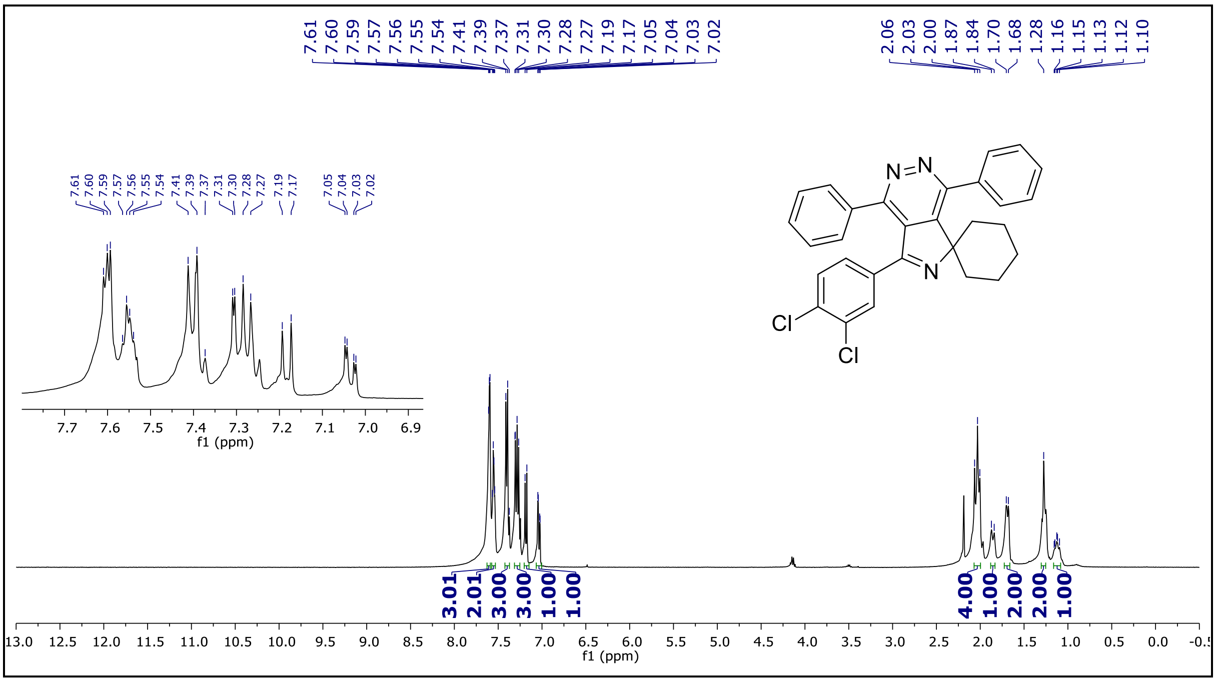

Figure S13. ^{1}H NMR spectrum of compound **SPP-7**.

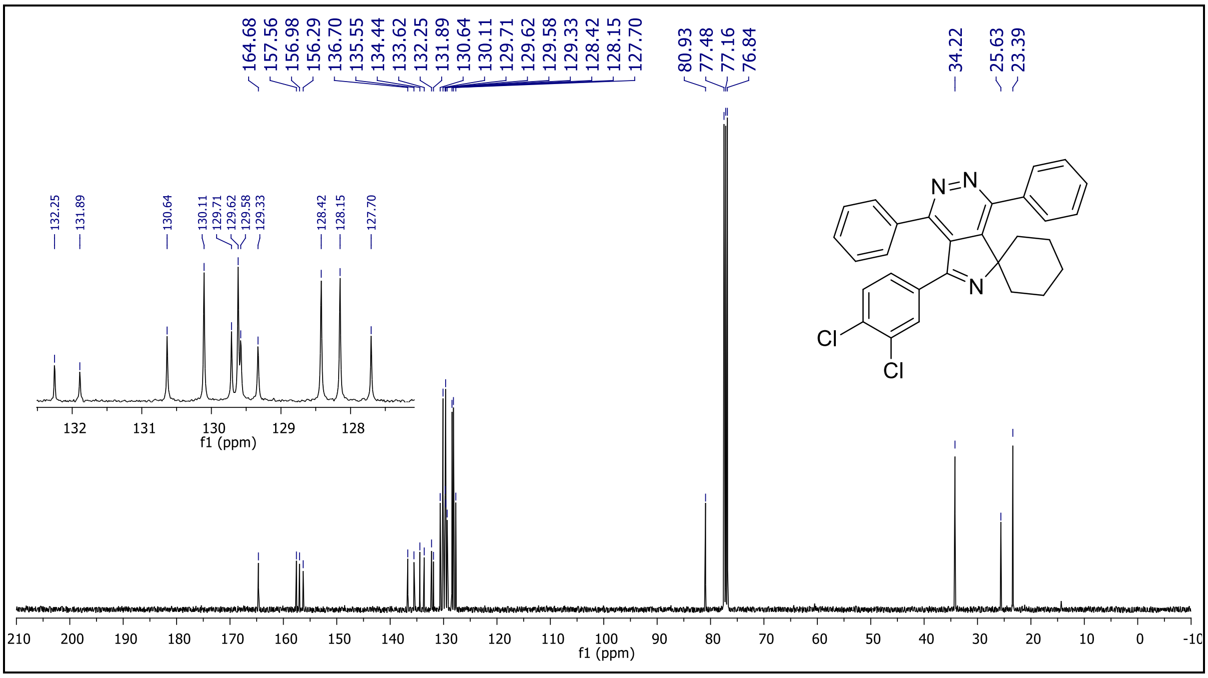

Figure S14. ^{13}C NMR spectrum of compound **SPP-7**.

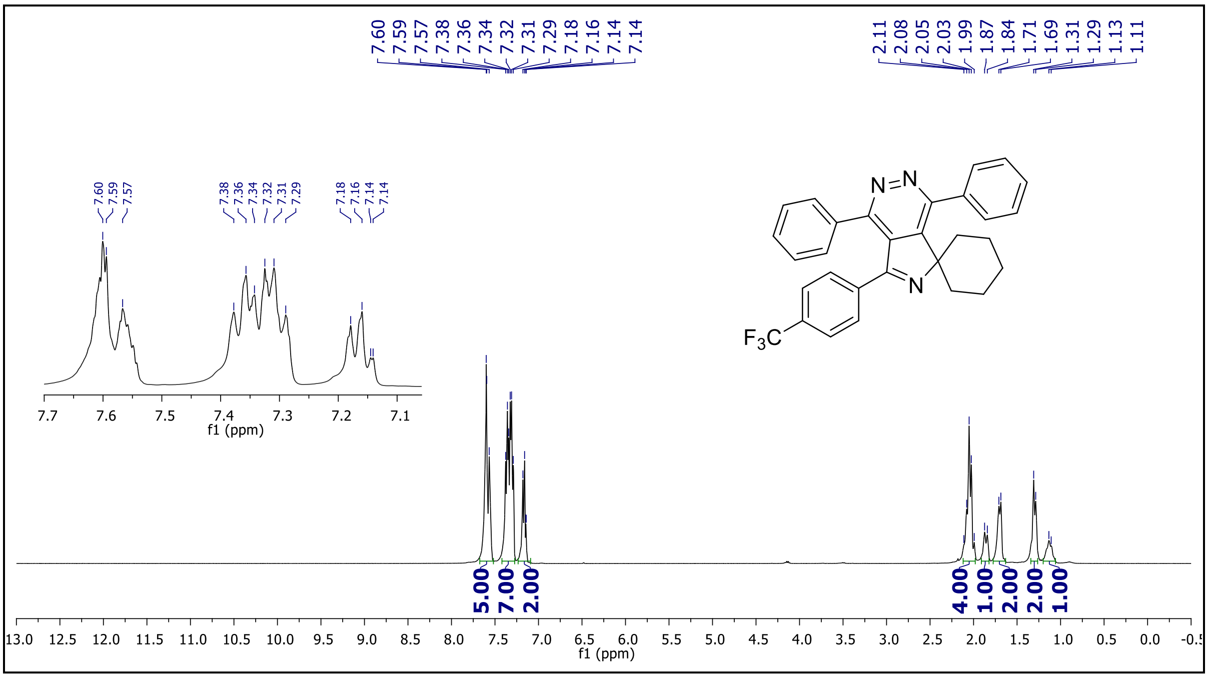

Figure S15. ^{1}H NMR spectrum of compound **SPP-8**.

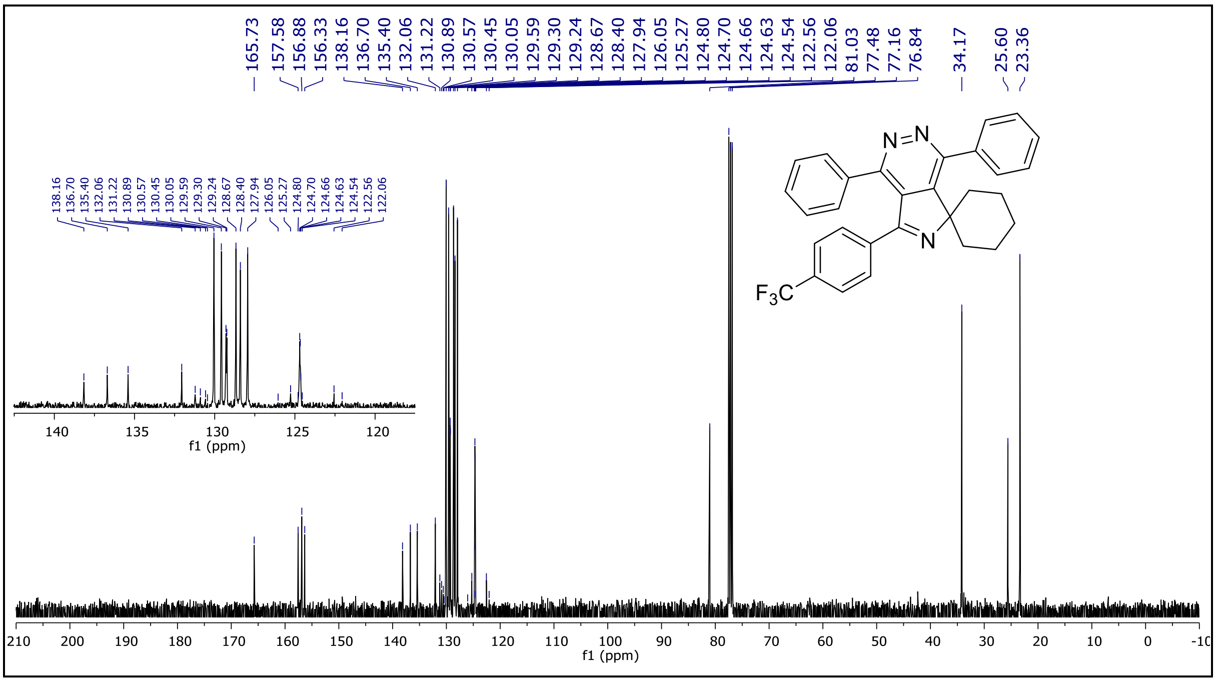

Figure S16. ^{13}C NMR spectrum of compound **SPP-8**.

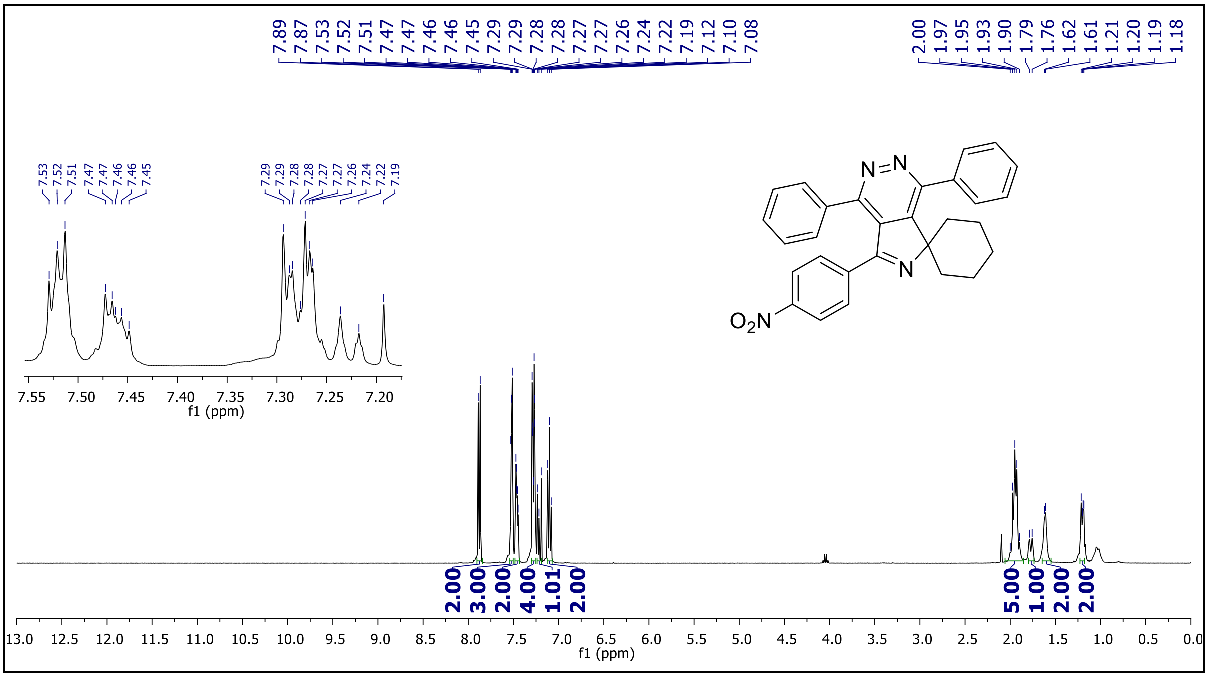

Figure S17. ^{1}H NMR spectrum of compound **SPP-9**.

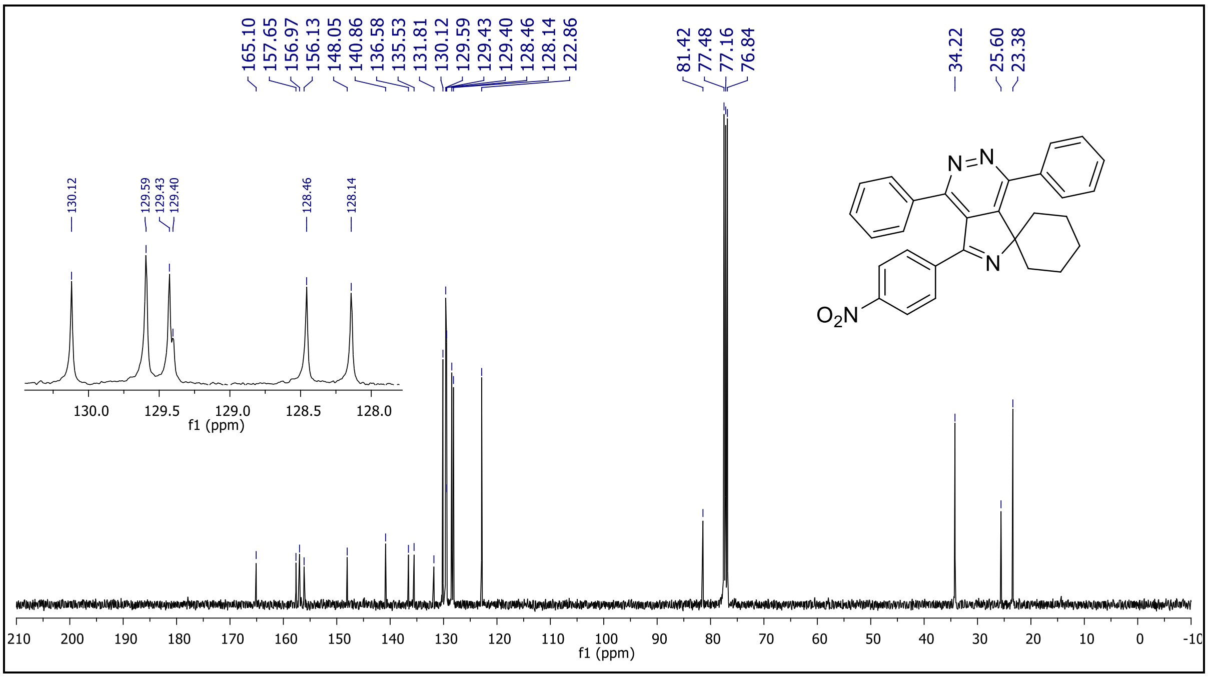

Figure S18. ^{13}C NMR spectrum of compound **SPP-9**.

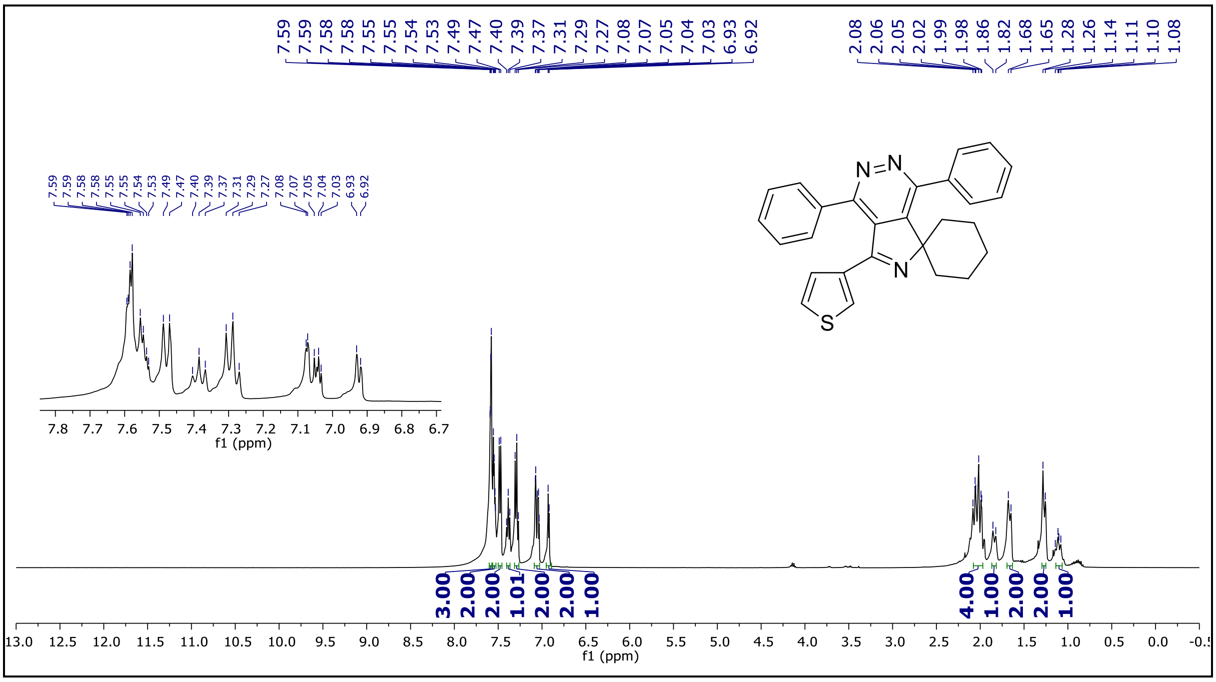

Figure S19. ^{1}H NMR spectrum of compound **SPP-10**.

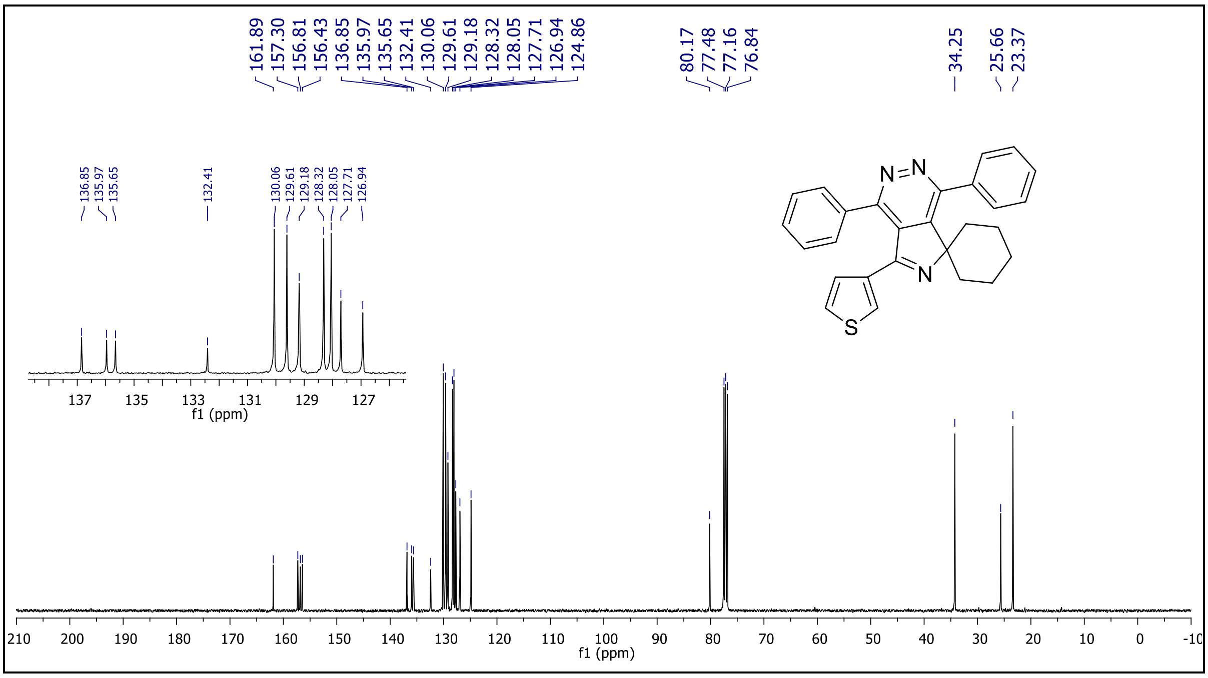

Figure S20. ^{13}C NMR spectrum of compound **SPP-10**.

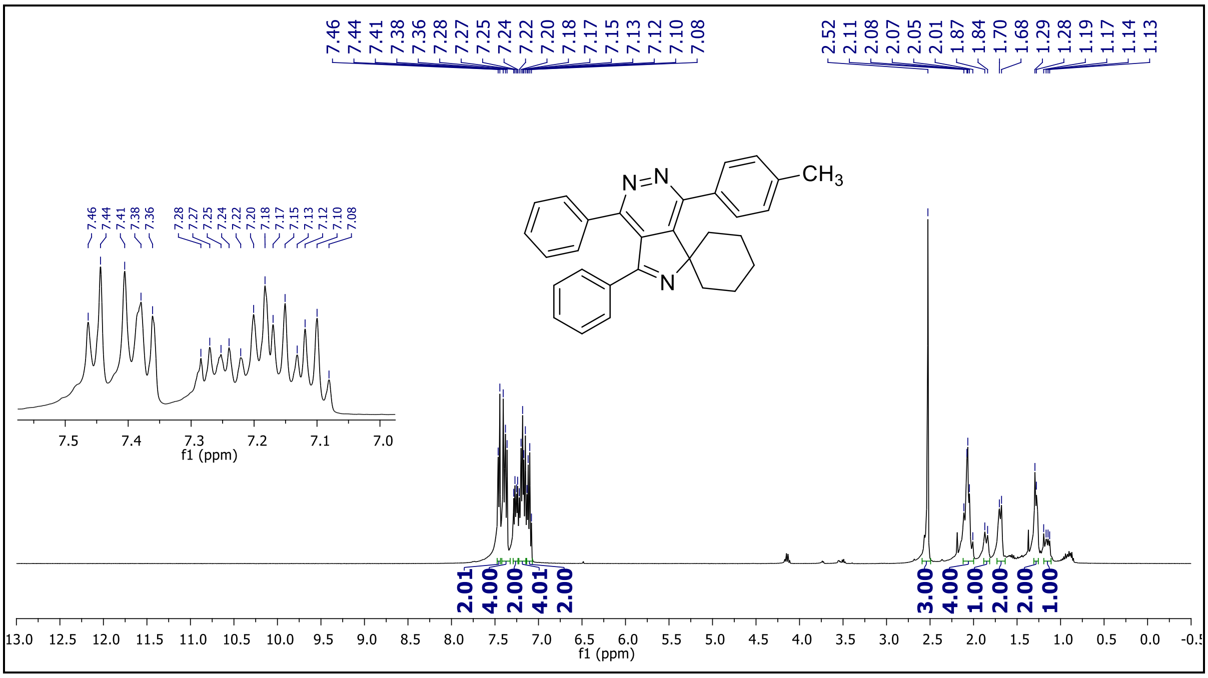

Figure S21. ^{1}H NMR spectrum of compound **SPP-11**.

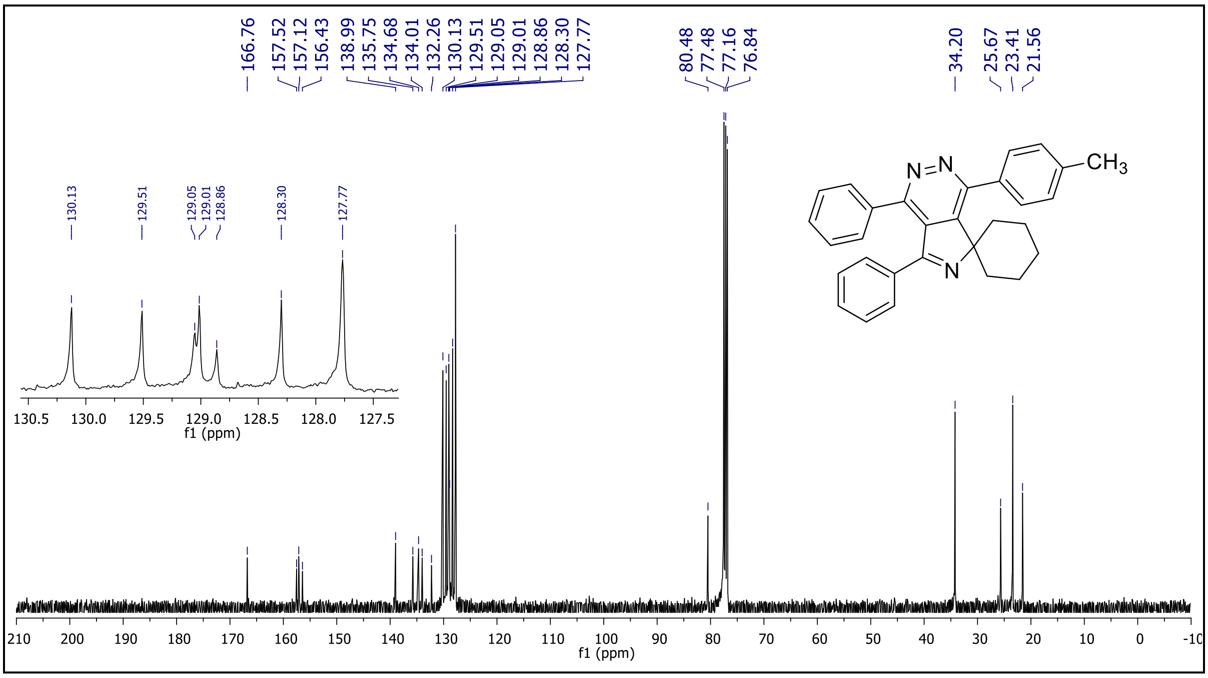

Figure S22. ^{13}C NMR spectrum of compound **SPP-11**.

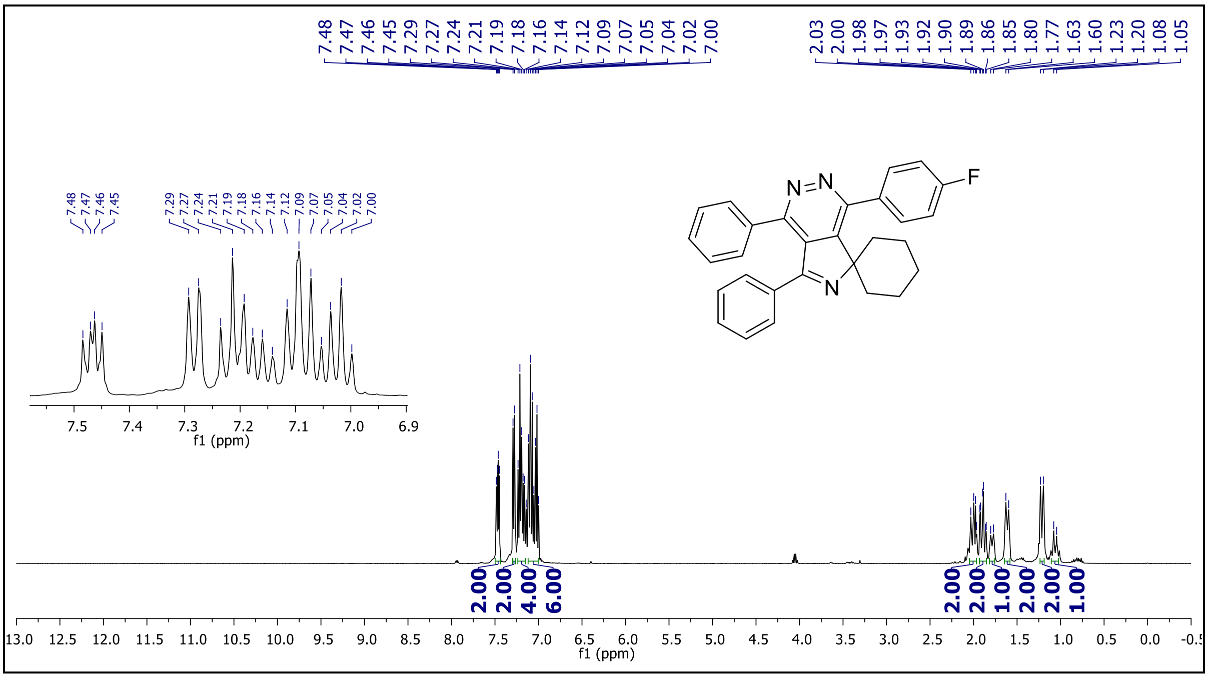

Figure S23. ^{1}H NMR spectrum of compound **SPP-12**.

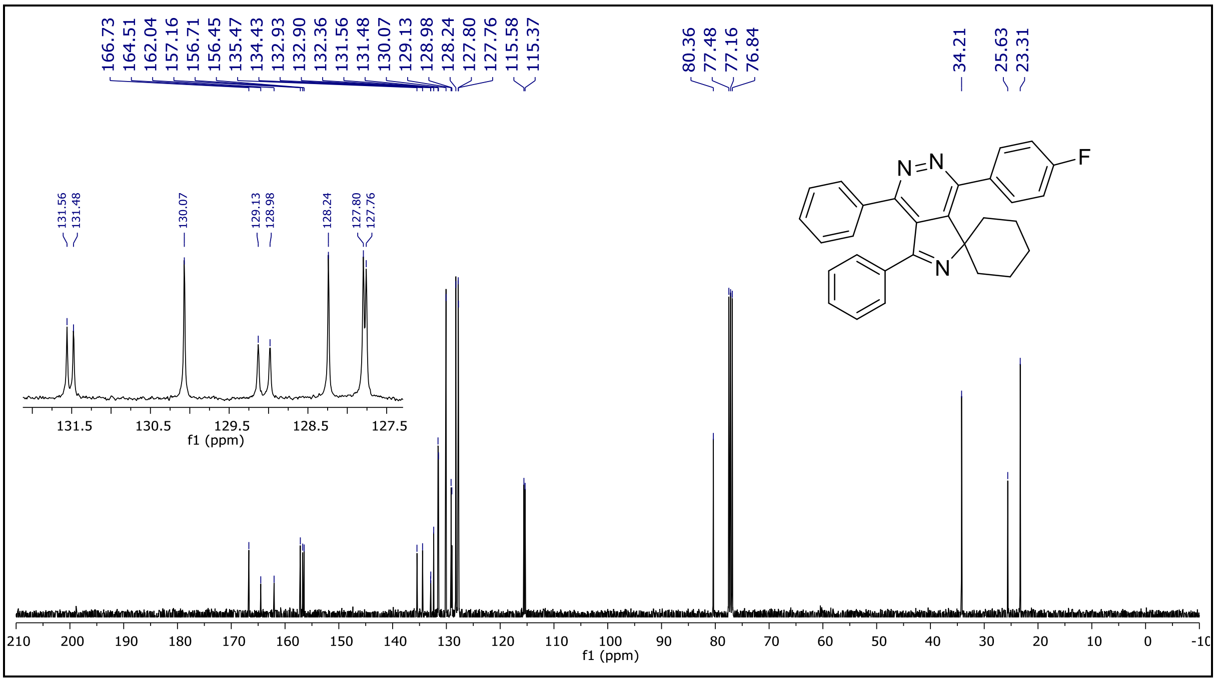

Figure S24. ^{13}C NMR spectrum of compound **SPP-12**.

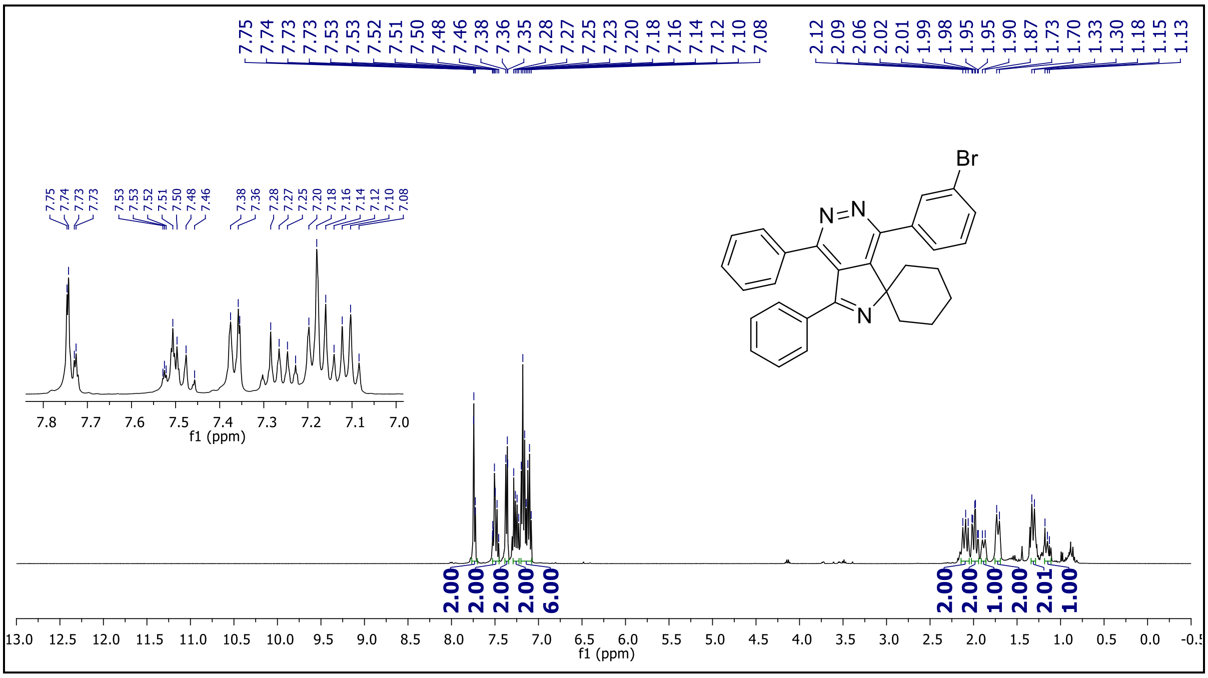

Figure S25. ^{1}H NMR spectrum of compound **SPP-13**.

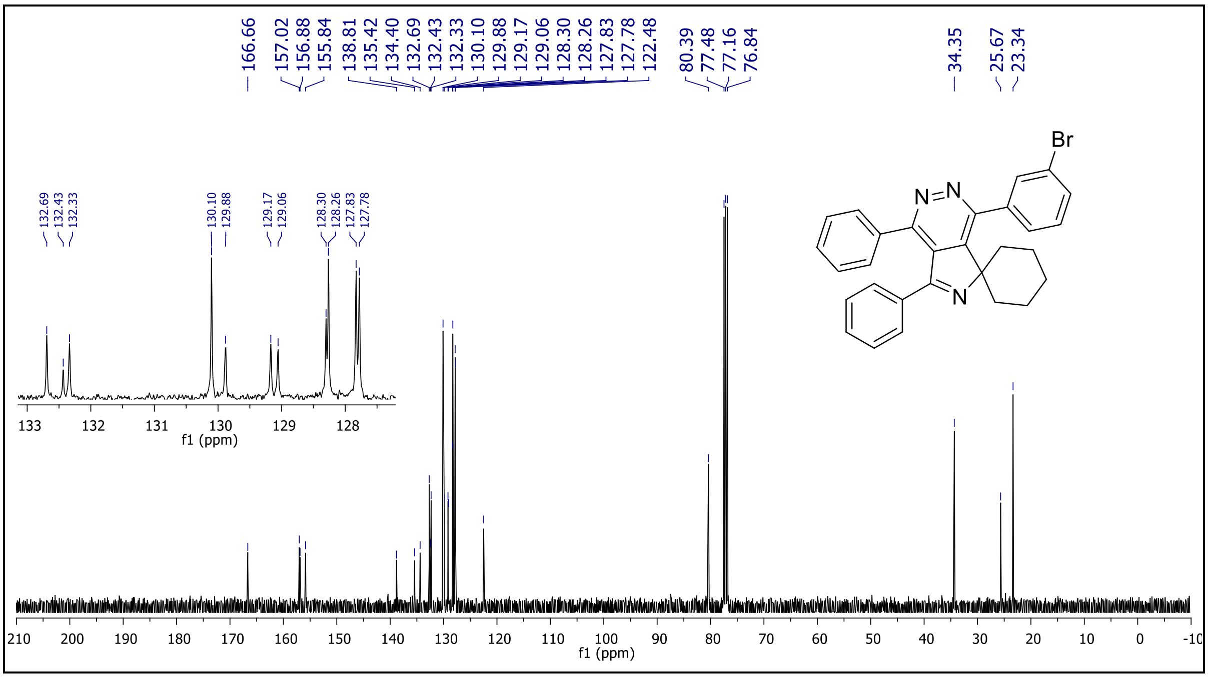

Figure S26. ^{13}C NMR spectrum of compound **SPP-13**.

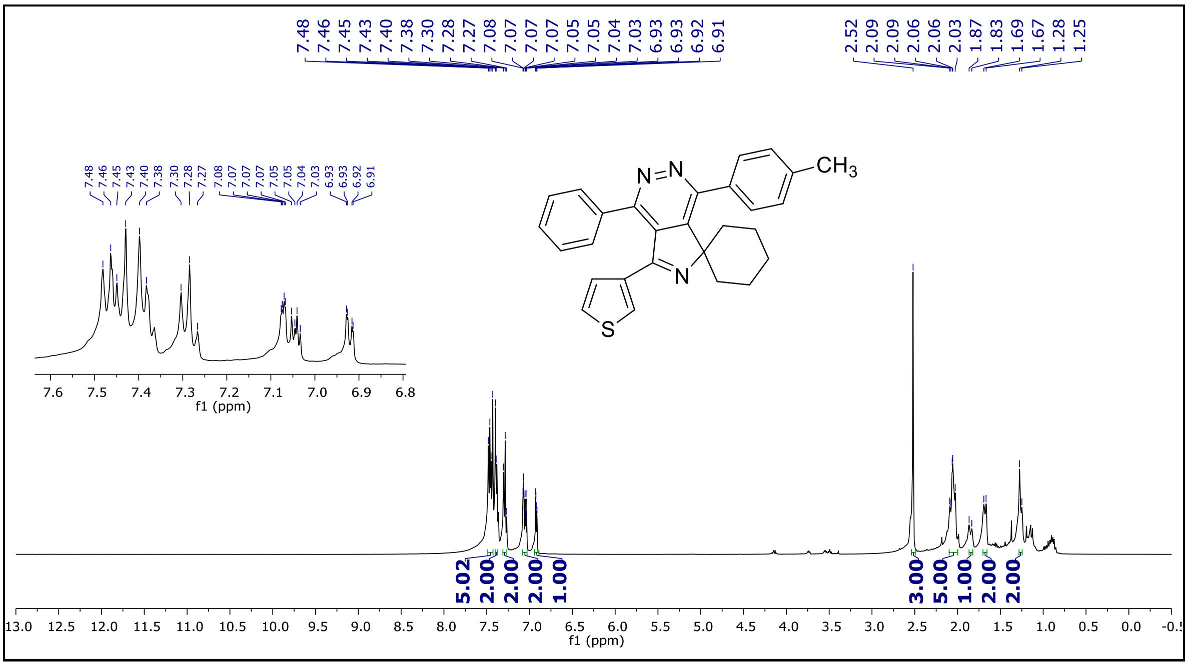

Figure S27. ^{1}H NMR spectrum of compound **SPP-14**.

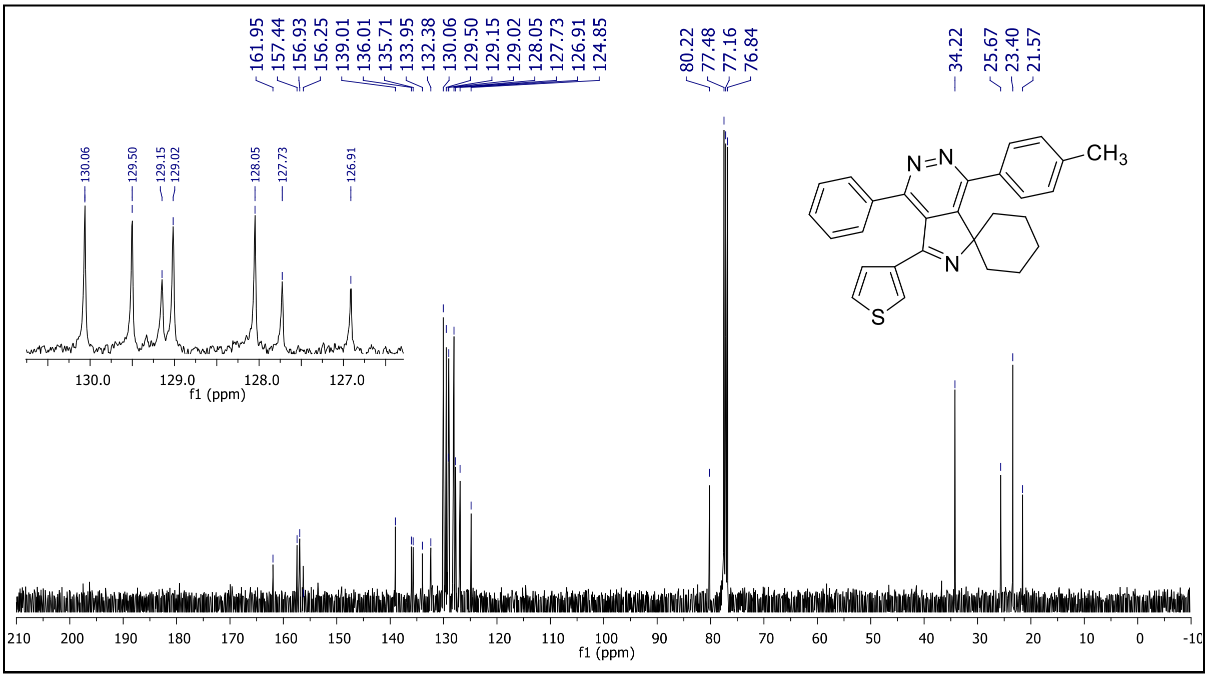

Figure S28. ^{13}C NMR spectrum of compound **SPP-14**.

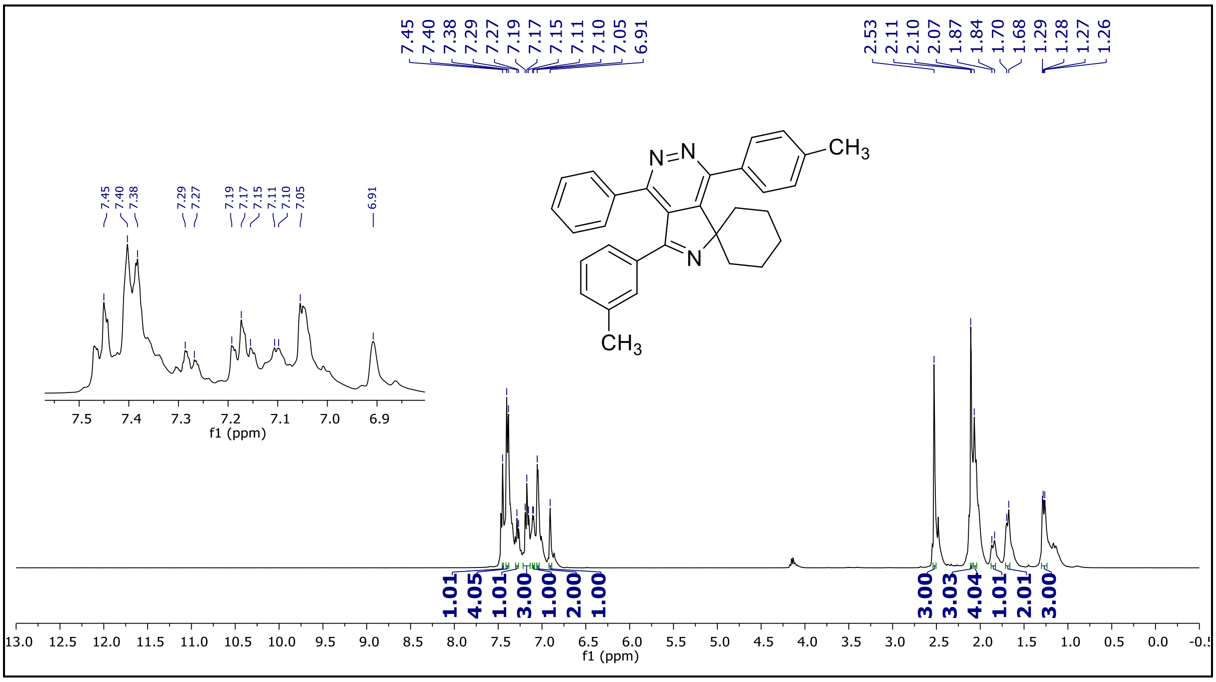

Figure S29. ^{1}H NMR spectrum of compound **SPP-15**.

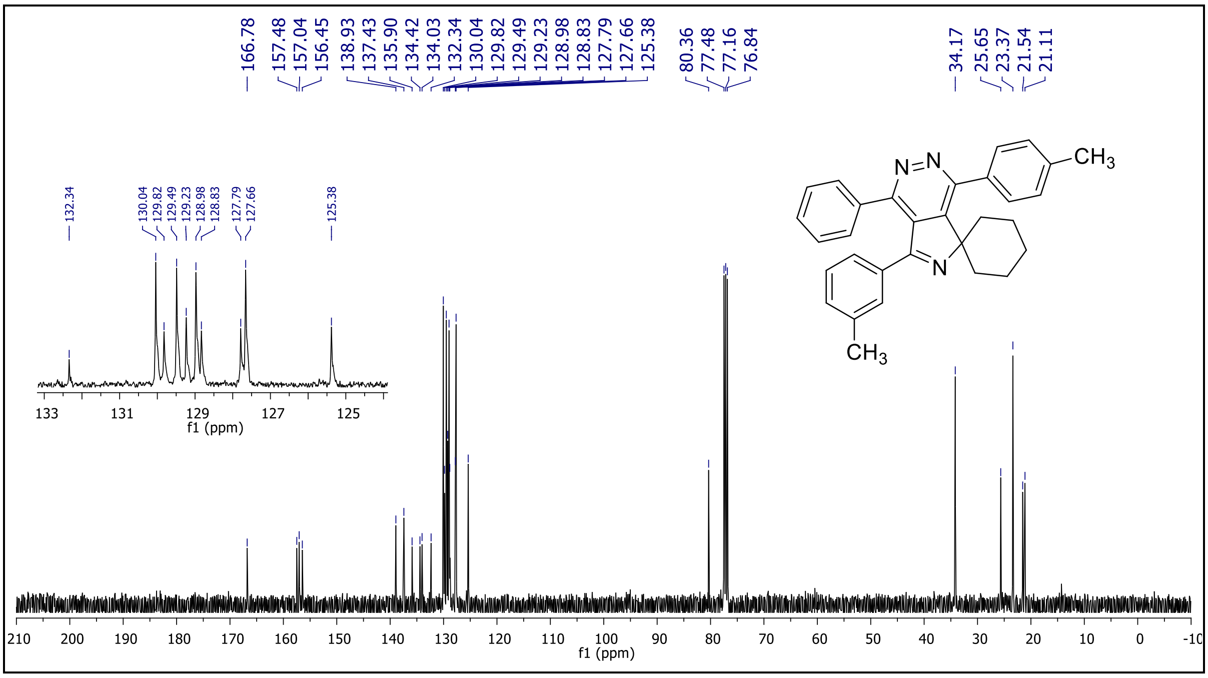

Figure S30. ^{13}C NMR spectrum of compound **SPP-15**.

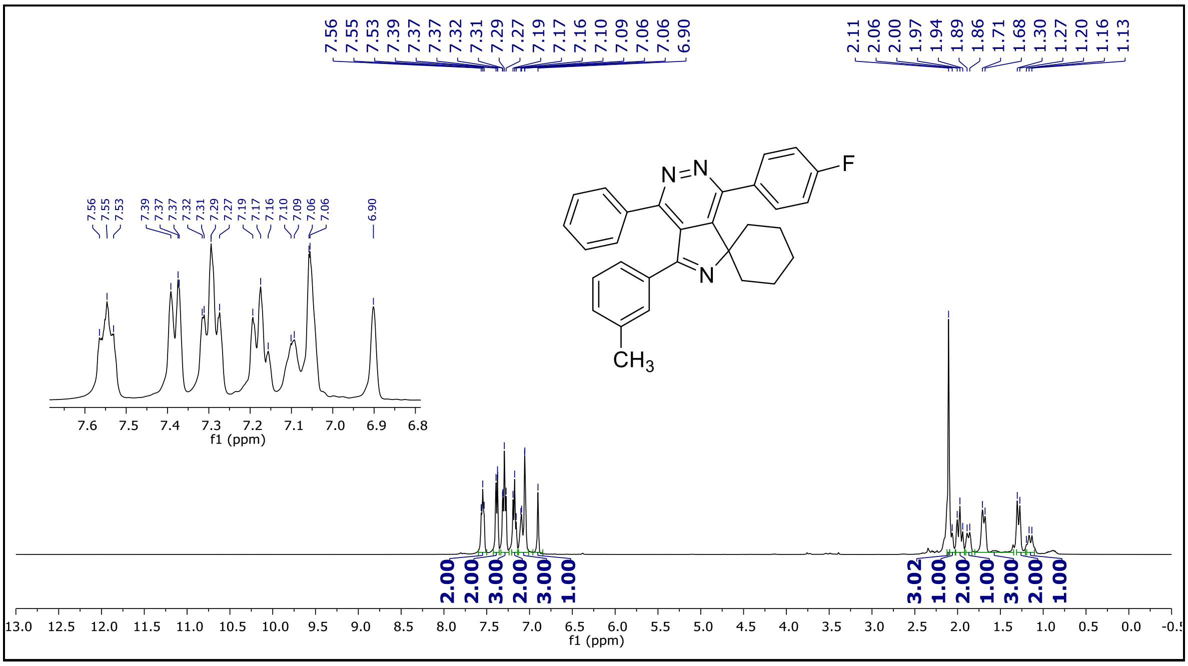

Figure S31. ^{1}H NMR spectrum of compound **SPP-16**.

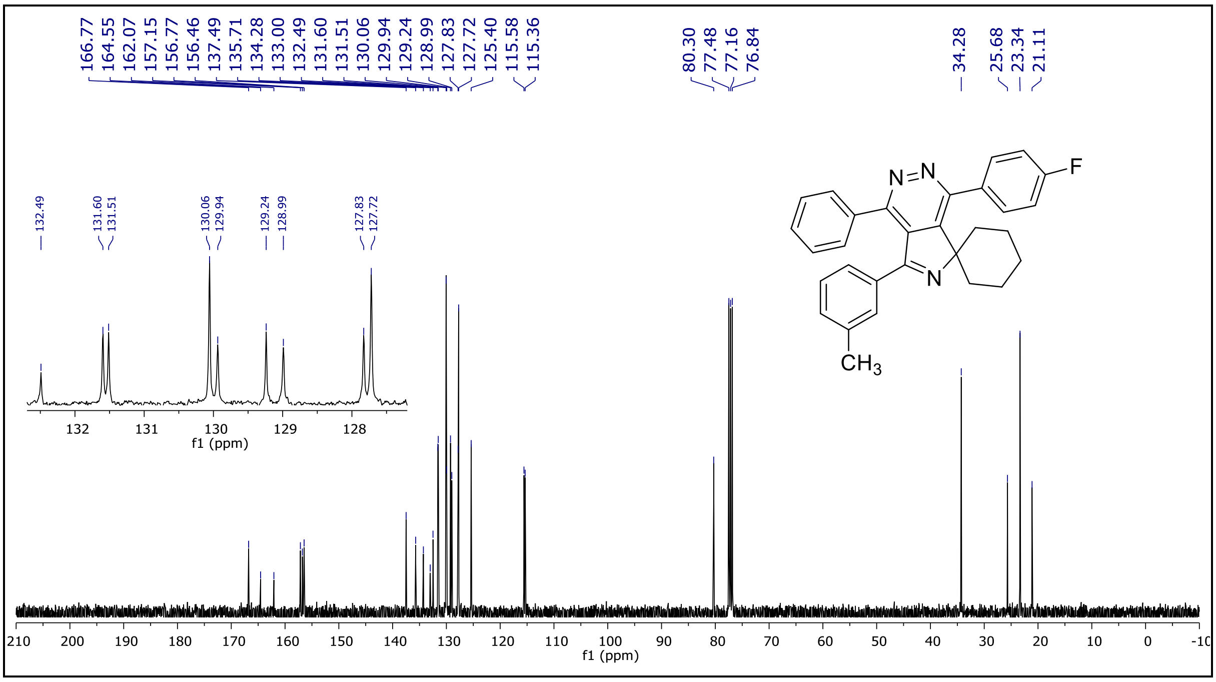

Figure S32. ^{13}C NMR spectrum of compound **SPP-16**.

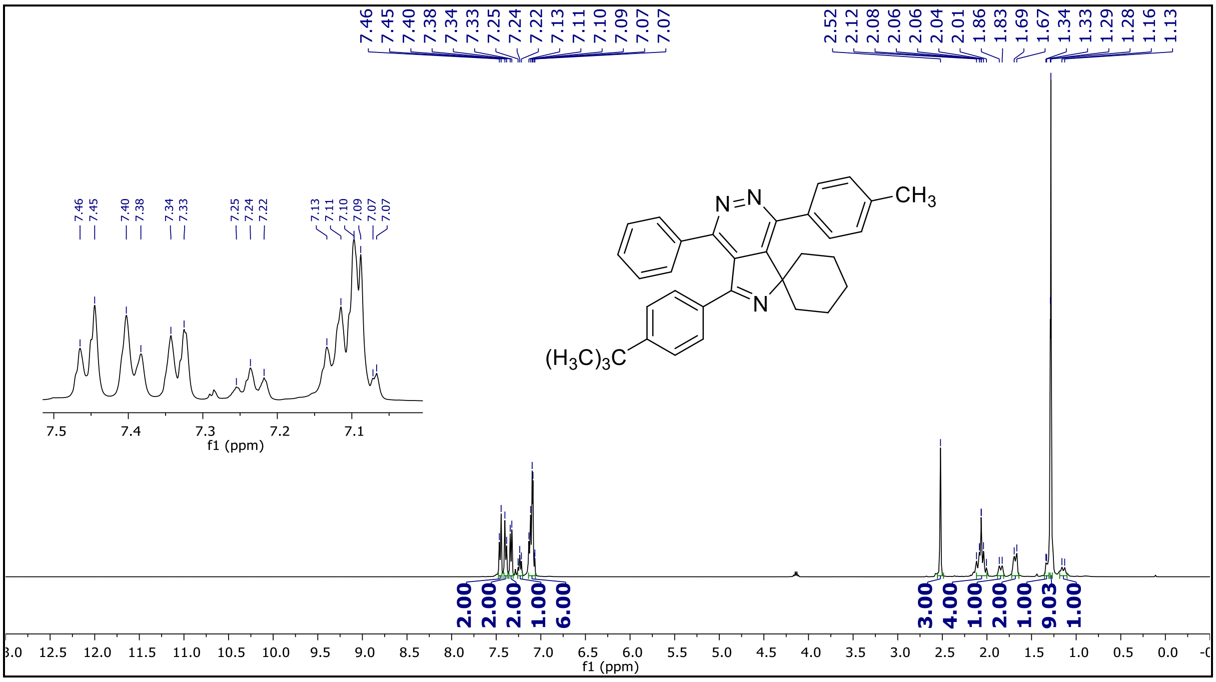

Figure S33. ^{1}H NMR spectrum of compound **SPP-17**.

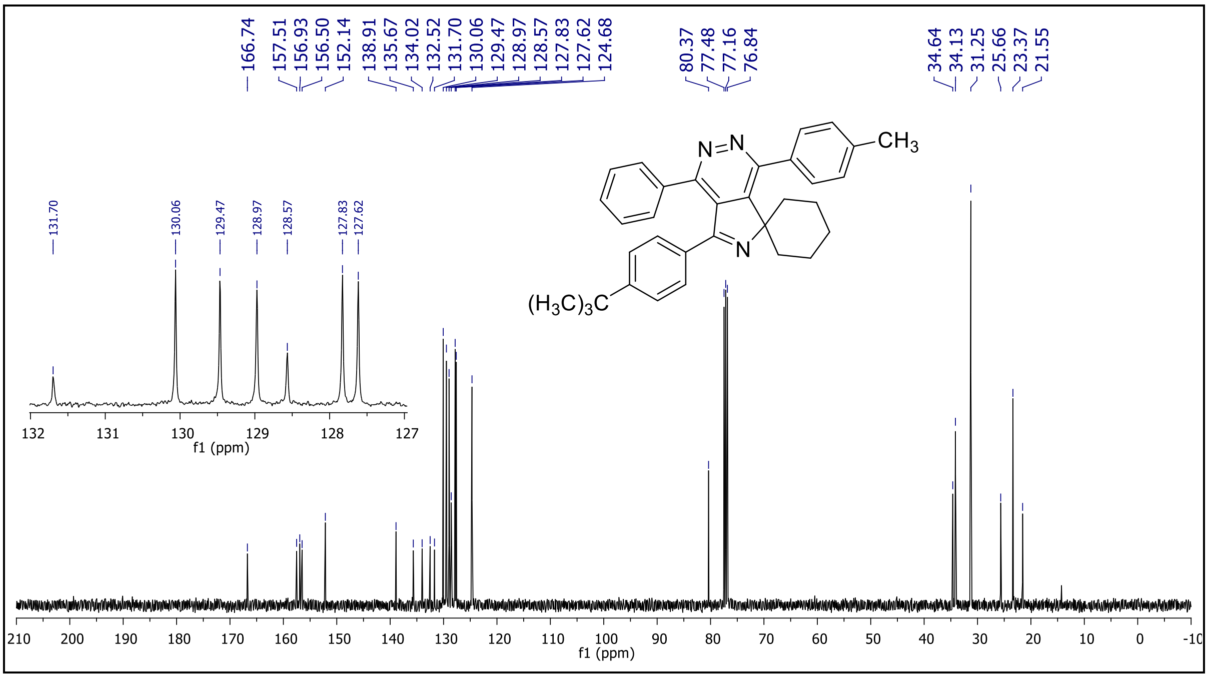

Figure S34. ^{13}C NMR spectrum of compound **SPP-17**.

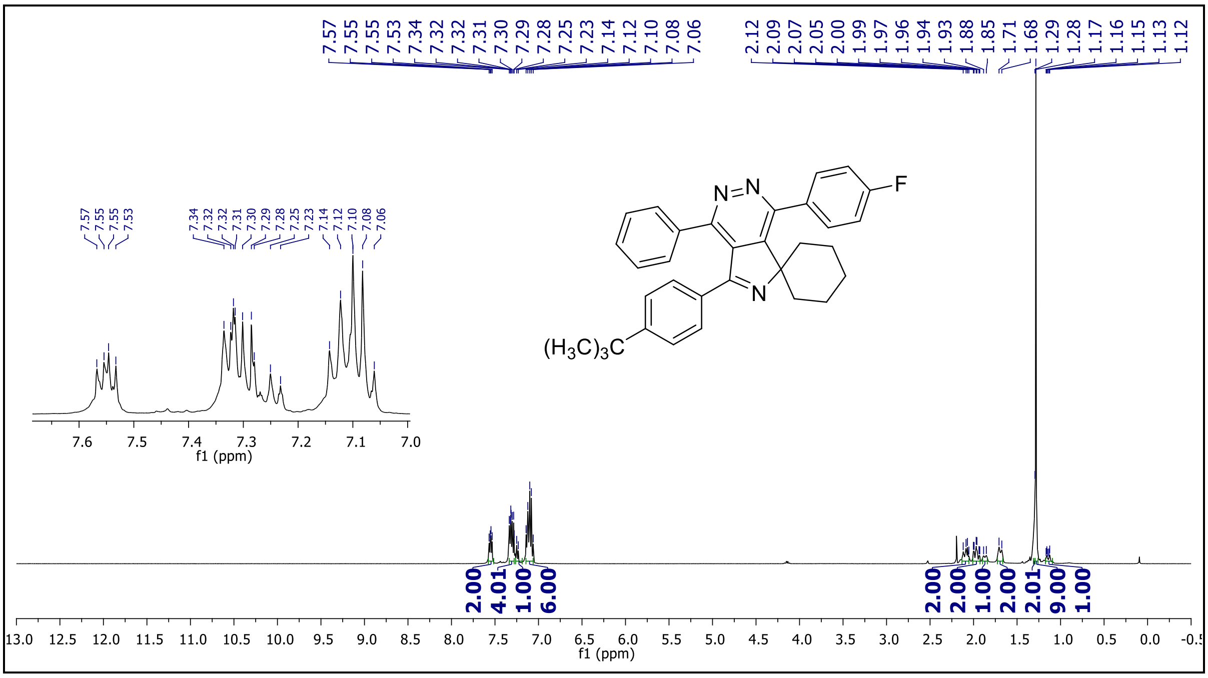

Figure S35. ^{1}H NMR spectrum of compound **SPP-18**.

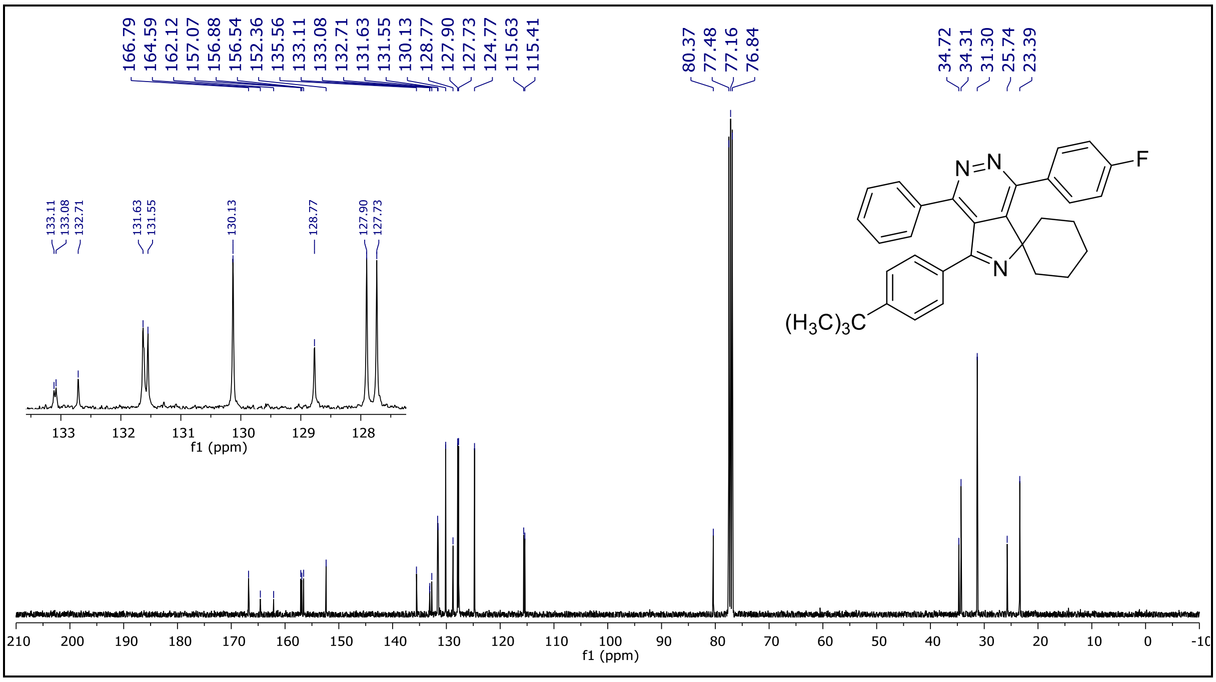

Figure S36. ^{13}C NMR spectrum of compound **SPP-18**.

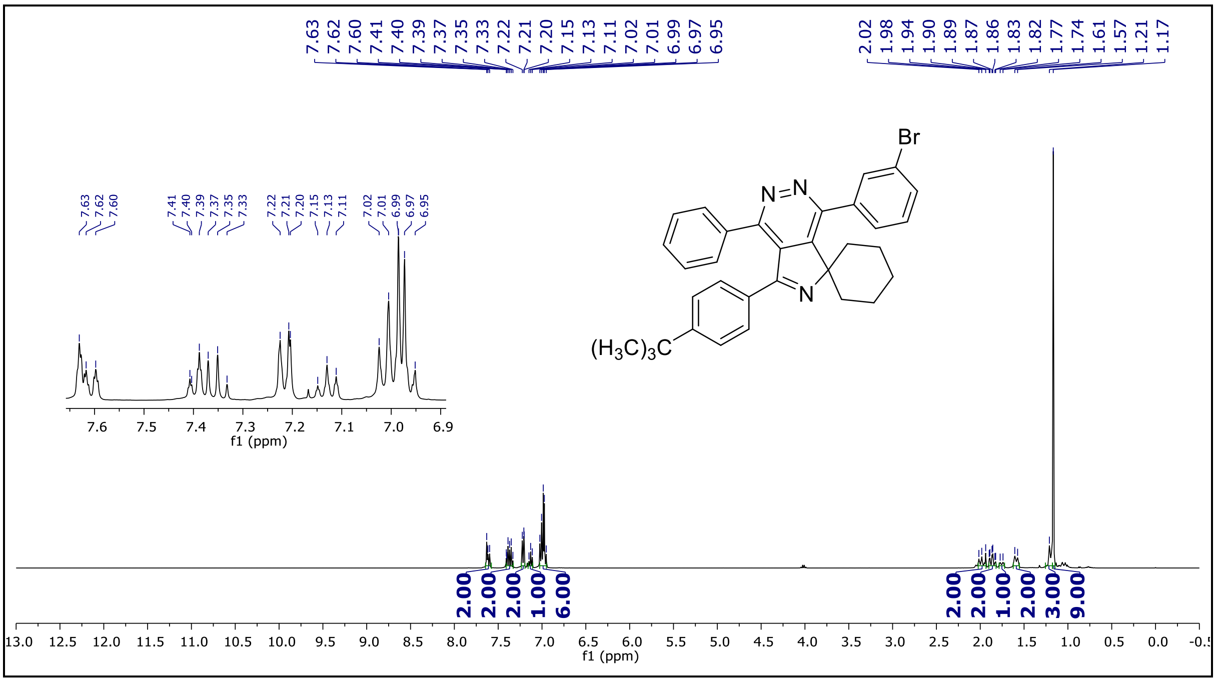

Figure S37. ^{1}H NMR spectrum of compound **SPP-19**.

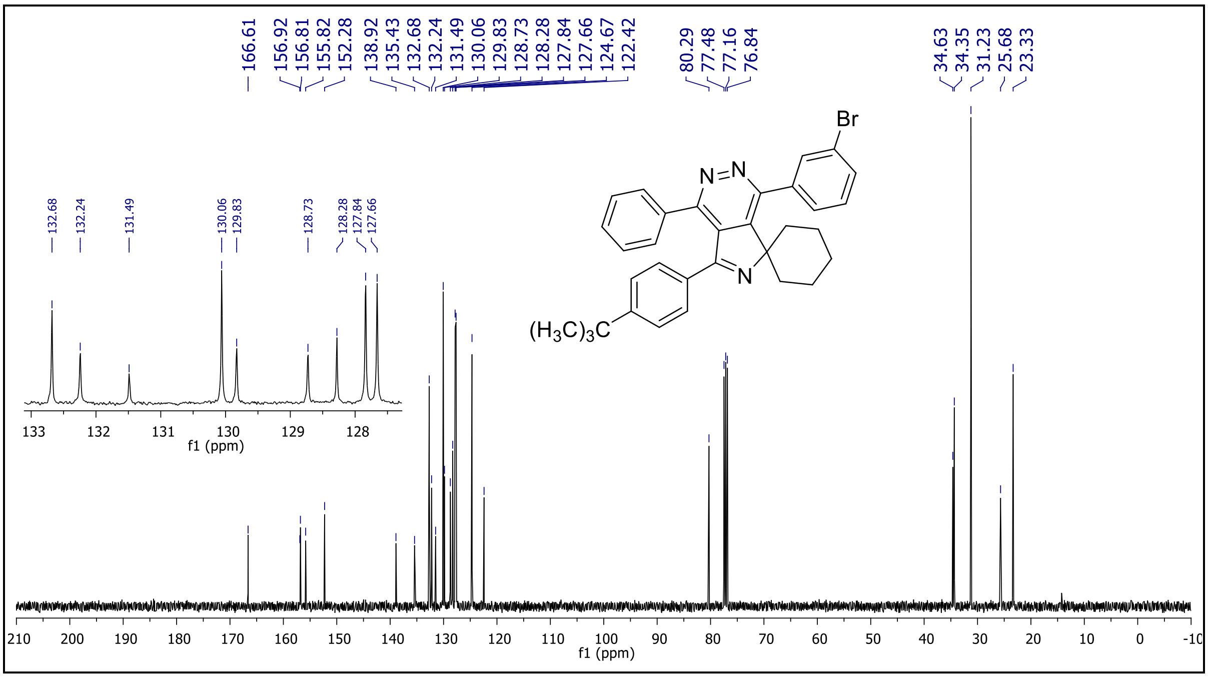

Figure S38. ^{13}C NMR spectrum of compound **SPP-19**.

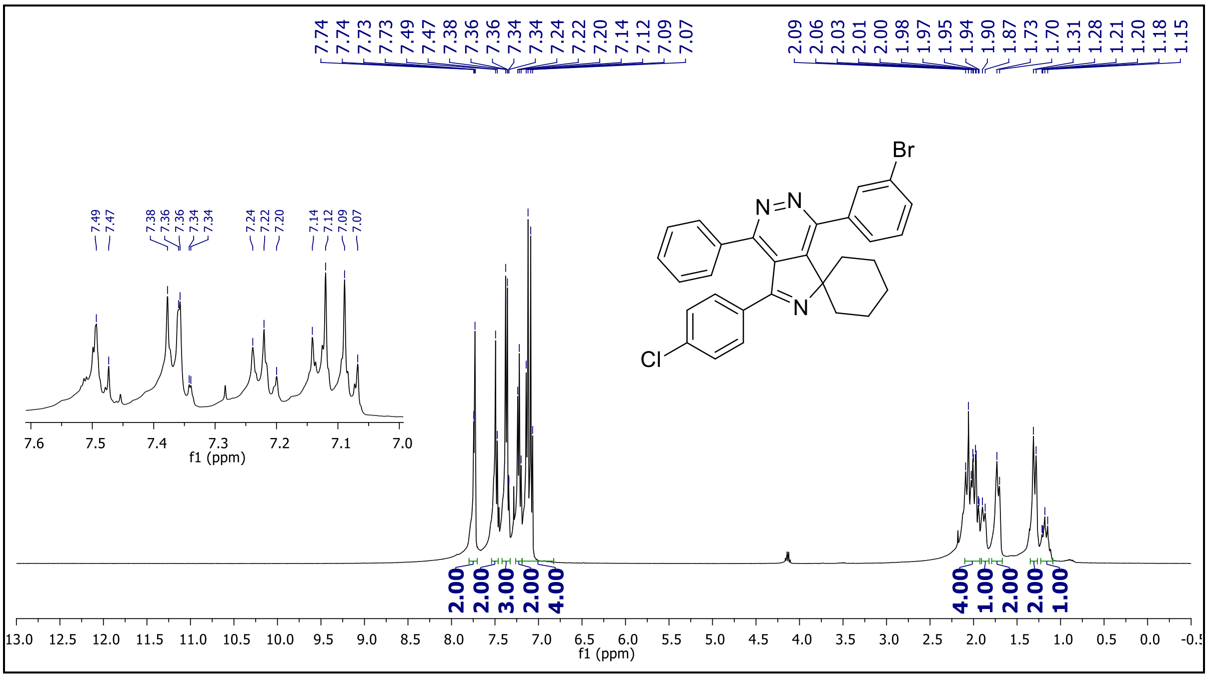

Figure S39. ^{1}H NMR spectrum of compound **SPP-20**.

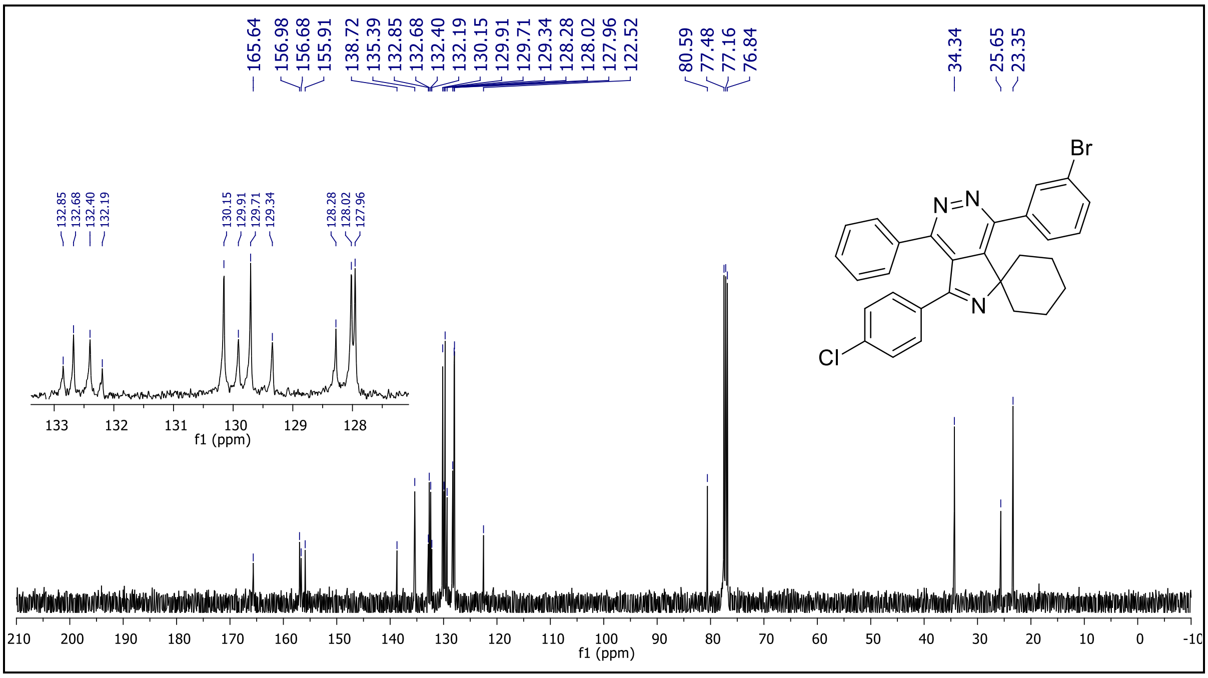

Figure S40. ^{13}C NMR spectrum of compound **SPP-20**.

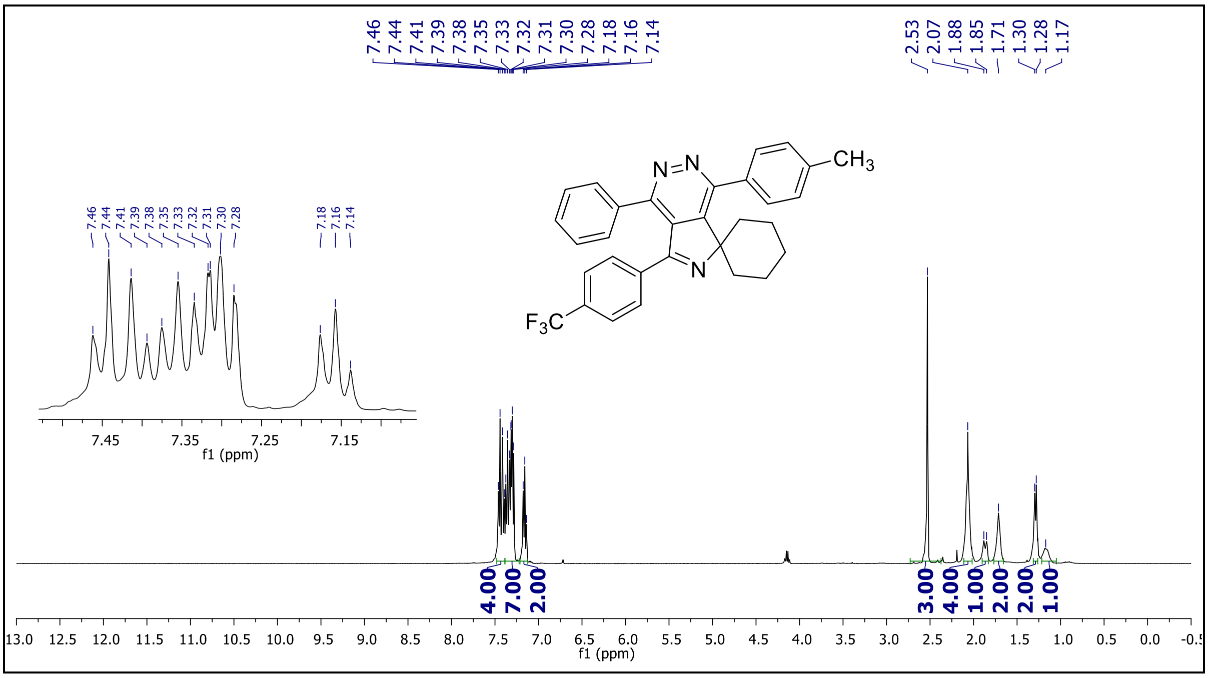

Figure S41. ^{1}H NMR spectrum of compound **SPP-21**.

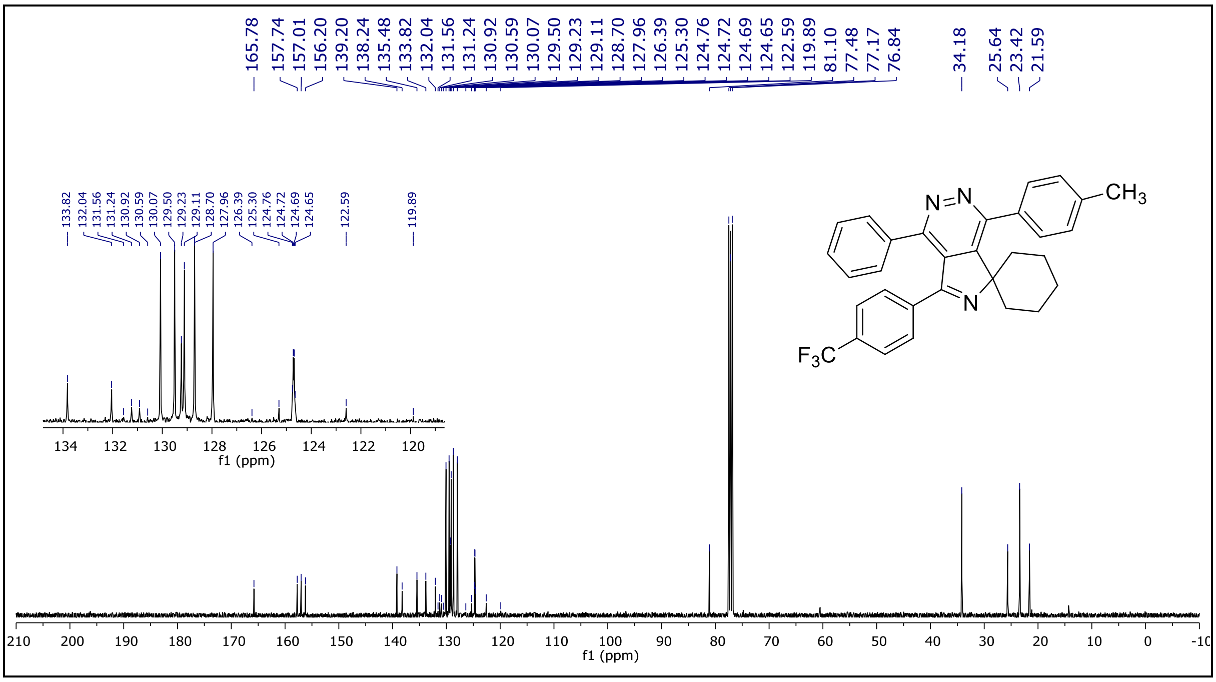

Figure S42. ^{13}C NMR spectrum of compound **SPP-21**.

References

1. Dundar, B. A.; Zora, M. A facile synthesis of a novel family of heterotricyclic hybrids: Spiro-pyrrolopyridazines. *Synth. Commun.* **2022**, *52*, 356-367. DOI: 10.1080/00397911.2021.2024575

2. For the synthesis of β-enaminones **2** and **3**, and spiro-2*H*-pyrroles (**SP**), see also: (a) Karadeniz, E.; Zora, M. Synthesis of 1-Azaspiro[4.5]deca-1,3-dienes from *N*-Propargylic β-Enaminones in Basic Medium. *Synthesis* **2019**, *51*, 2157-2170. DOI: 10.1055/s-0037-1611723 (b) Karadeniz, E.; Zora, M. One-Pot Synthesis of Spiro-2*H*-pyrroles from *N*-Propargylic β-Enaminones. *Synlett* **2019**, *30*, 1231-1236. DOI: 10.1055/s-0037-1611816

3. Chinchilla, R.; Najera, C. The Sonogashira Reaction: A Booming Methodology in Synthetic Organic Chemistry. *Chem*. *Rev*. **2007**, *107*, 874–922. DOI: 10.1021/cr050992x
